# Comparing experience intensity of AR and VR for contrasting phobia stimuli

**DOI:** 10.1007/s10055-026-01450-y

**Published:** 2026-08-11

**Authors:** Ruoqing Yang, Chris Greenhalgh

**Affiliations:** School of Computer Science, University of Nottingham, Nottingham, England

**Keywords:** Virtual Reality (VR), Augmented Reality (AR), Exposure-based therapy, Experience intensity, Technology choice

## Abstract

Exposure-based therapy is a common psychological intervention for phobias and anxiety disorders, and exposure-based therapy using virtual reality (VR) and augmented reality (AR) have been evaluated as practical extensions of this method, producing similar experiences through modelled stimuli. This study compares participants’ responses to VR and AR exposure under different types of stimuli. The study investigates two types of fear-based stimuli: spiders and contamination, for which both VR and AR prototypes were developed. A within-subjects experimental design was employed, involving 31 participants drawn from a healthy adult student population who reported their experience intensity and qualitative feedback following exposure to virtual stimuli across both modalities. The type of technology significantly affected the experience intensity: VR elicited a significantly higher experience intensity overall, although the strength of this effect varied depending on the stimulus type. Specifically, VR produced a much stronger experience in the contamination scenario, while AR performed comparably and showed ecological advantages in the spider scenario. We suggest that future mixed-reality exposure system designs could integrate both AR and VR components in a staged approach. AR may be more suitable for early-stage or animal-focused exposure scenarios, whereas VR is better suited for scenarios that demand complete environmental control.

**Supplementary Information:**

The online version contains supplementary material available at https://doi.org/10.1007/s10055-026-01450-y.

## Introduction

Exposure-based therapy, a form of cognitive behavioural therapy, is a psychological intervention for fears and phobias. It employs the principle of gradual exposure to systematically introduce aversive stimuli to individuals in order to reduce the intensity of their emotional response over time (Foa et al., 2008). Traditional exposure-based therapy predominantly involves in vivo exposure, where a therapist guides a patient to systematically confront sources of aversion through a habituation process. Although traditional exposure-based therapy has been shown to be effective, setting up such scenarios in a therapeutic context can be challenging and carries potential safety risks. Furthermore, as this method necessitates direct confrontation with aversive stimuli, it presents emotional challenges to patients, potentially impeding some patients’ continued participation due to intense anxiety responses (Gunter and Whittal 2010).

Given these drawbacks of traditional exposure-based therapy, immersive technologies, such as Virtual Reality (VR) and Augmented Reality (AR), are being explored for Exposure-based Therapy using Virtual stimuli (ET-V) to treat specific phobias (sometimes inaccurately referred to as Virtual Exposure Therapy). VR creates fully immersive environments that allow controlled and realistic scenarios without real-world risks, making it suitable for contexts that are difficult to replicate in traditional settings (Carvalho et al. 2010; North et al. 1998). AR integrates virtual objects into the real physical environment of the user (Li et al. 2014), and has been primarily explored for animal-focused exposure prototypes. Empirical evidence supports the efficacy of both technologies in exposure-based therapy, demonstrating reductions in anxiety responses over time (see Sect.  2.2), suggesting that exposure to virtual stimuli may serve as a feasible complement to traditional methods.

According to Emotional Processing Theory (Foa and Kozak 1986), effective exposure requires the activation of underlying aversive response structures. Whether such activation occurs depends not only on the stimulus itself but also in the case of virtual or modelled stimuli on the properties of the medium delivering it. Research on presence suggests that a sufficient sense of being there is needed before virtual stimuli can trigger emotional responses (Felnhofer et al., 2015), and that the perceived realism of the scenario further shapes the intensity of those responses (Chirico and Gaggioli 2019). We consider these properties together as a composite construct “experience intensity,” which comprises three dimensions: realism and immersion, behavioural and spatial presence, and emotional intensity (see Sect.  3.5).

VR and AR construct perceptual experiences through different mechanisms, and may therefore differ in the experience intensity they produce for a given stimulus. Therefore, before deploying these systems in clinical settings, it is worth understanding whether the two technologies differ in their capacity to elicit experience intensity across different stimulus types. This paper reports a comparative investigation of VR and AR with a non-clinical population across two fear-relevant stimulus conditions: spiders and contamination. These stimuli were selected because they have qualitatively distinct spatial (focal) and contextual properties. Contamination is highly dependent on the surrounding environmental context, whereas spider stimuli involve a dynamic object intruding into the immediate physical space of the user. Both stimuli have been effectively utilised in previous exposure-based research using virtual stimuli (see Sect.  2.4).

The study focuses on two main questions:

### Q1.

When used for virtual stimuli for exposure, is there a significant difference in the experience intensity elicited by AR and VR technologies?

### Q2.

If a difference exists, does the difference in experience intensity elicited by AR and VR also depend on the specific modelled stimulus (here spiders vs. contamination)?

By directly comparing AR and VR across two stimulus conditions, this study aims to determine whether one technology produces greater experience intensity overall, or whether it depends on the specific stimulus type. The findings may offer practical guidance for developers and clinicians in selecting appropriate tools based on the requirements of the targeted exposure scenario.

## Literature review

The literature relevant to this study focuses on exposure-based therapy and the application of immersive technologies in creating virtual stimuli. First, the limitations of traditional exposure-based therapy and the emergence of exposure approaches based on virtual reality and augmented reality are discussed. The review then explores mechanisms of emotion induction in virtual environments, providing a theoretical basis. It further compares the use of these two technologies in exposure-based therapy and identifies a gap in the existing literature.

### Exposure-based therapy and its limitations

Exposure-based Therapy is an effective method for treating phobias and has been widely applied in the field of psychotherapy. The therapy’s core mechanism involves repeated exposure experiences, where the brain learns that fear-inducing situations need not have negative outcomes, thereby reducing phobia symptoms (Wolitzky-Taylor et al. 2008). This therapy is grounded in classical behaviourist theories, specifically the concepts of conditioned reflexes and learned helplessness.

In traditional In-Vivo Exposure-based Therapy (IVET), therapists help patients face their feared objects or situations in a safe physical environment. The process of exposure-based therapy typically includes several steps. Initially, the therapist and patient create an exposure hierarchy, listing situations from the least to the most fear-inducing. The patient then gradually encounters these situations under the therapist’s guidance (Schumacher et al. 2015). For instance, a patient with a specific phobia might be gradually exposed to the source of their fear, progressing from images and videos to real-life situations, with increasing levels of exposure intensity. In each step, the patient remains in the situation until their fear gradually diminishes, a process known as “extinction” (Abramowitz 2013). The effectiveness of exposure-based therapy has been validated in numerous studies. Research has shown that exposure-based therapy significantly benefits specific phobias, such as social anxiety, agoraphobia, and spider phobia (Krzystanek et al. 2021).

However, traditional exposure-based therapy faces some challenges. First, the recreation of these situations during therapy can be constrained by time, location, and resources, potentially reducing the therapy’s efficacy (Foa et al., 2008). Additionally, the realism and intensity of fear-inducing situations can be limited in therapeutic settings, which may affect the therapy’s effectiveness (Meuret et al. 2012). Also, for some patients, direct exposure to fear-inducing situations can be too intense, making it difficult for them to persevere with the treatment, thus affecting the outcomes (Culver et al. 2012).

### Exposure-based therapy using virtual stimuli

To address the issues present in traditional exposure-based therapy, Exposure-based Therapy using Virtual stimuli (ET-V) has emerged as a practical complement. ET-V allows for more flexible control and reproduction of aversive situations, potentially providing a safer therapeutic environment (Kim et al. 2016). As there is no need for patients to be physically exposed to the objects or places they wish to avoid, potential safety risks are reduced. By offering a virtual alternative, patients may be able to engage in exposure-based therapy with lower psychological stress, which can improve adherence and treatment effectiveness (Powers and Emmelkamp 2008).

Exposure conducted using VR stimuli are referred to as Exposure-based Therapy using Virtual Reality (ET-VR), while those using AR are known as Exposure-based Therapy using Augmented Reality (ET-AR). VR creates fully immersive environments by replacing the user’s entire perception of their physical surroundings with a computer-generated scene, which in some cases can be difficult to distinguish from reality. This sense of immersion opens up a range of potential applications in psychological treatment (Diemer et al. 2015). VR experiences typically require dedicated headsets such as the Meta Quest or Apple Vision Pro. AR, by contrast, enriches the real world by overlaying virtual content onto the user’s actual environment, calculated by aligning a virtual camera with the user’s perspective. This allows virtual objects such as 3D models, images, and videos to appear alongside real-world surroundings. AR can be delivered through everyday devices such as smartphones, or through dedicated hardware including AR headsets such as the Microsoft HoloLens and mixed reality headsets such as the Meta Quest 3, which support both AR and VR modes. Both technologies can model aversive stimuli in a controlled and repeatable manner, and allow therapists to adjust variables such as background sounds, lighting, and the proximity of the aversive stimulus to meet individual patient needs (Bouchard et al. 2014). Patients can also conduct exposure exercises at home or in their daily environment, which is beneficial for those unable to regularly visit therapy centres due to time or location constraints.

From a technological perspective, ET-VR uses fully immersive virtual environments to gradually expose patients to aversive stimuli and has been applied to treat a range of conditions, including contamination fears, acrophobia, fear of flying, and animal-focused scenarios such as spiders (Fajnerová et al. 2023; Freeman et al. 2018; Garcia-Palacios et al. 2002; Rothbaum et al. 2006; Tortella-Feliu et al. 2011). Its immersive nature allows fear-inducing situations to be modelled in a safe and controlled environment, although the high level of immersion may also lead to discomfort or motion sickness and requires specialised equipment (Boeldt et al. 2019; Mevlevioğlu et al. 2022). In contrast, ET-AR combines virtual stimuli with real-world environments, allowing patients to confront fear-related objects within their actual surroundings. ET-AR has been applied to treat small animal scenarios such as spiders, cockroaches, and moths, as well as contamination scenarios (Botella et al. 2016; Juan et al. 2005; Jurcik et al. 2024; Lohse et al. 2023; Wrzesien et al. 2015). Because AR operates within the real environment, it may offer less environmental control and immersion than fully virtual environments (Baus and Bouchard 2014).

Empirical evidence supports the efficacy of both modalities across a range of anxiety-related conditions. Meta-analyses and systematic reviews indicate that ET-VR produces outcomes generally comparable to traditional in vivo exposure-based therapy, with evidence demonstrating reductions in anxiety symptoms and fear responses across conditions including specific phobias, social anxiety disorder, and PTSD (Carl et al. 2019; Horigome et al. 2020; Kothgassner et al. 2019; Krzystanek et al. 2021; Rothbaum et al. 2006; Suso-Ribera et al. 2019). Importantly, these effects have been observed even in participants who did not respond adequately to traditional methods, suggesting that exposure-based therapy using virtual stimuli may reach patient groups for whom in vivo therapy is not feasible (Powers and Emmelkamp 2008). ET-AR has similarly been found to achieve effectiveness comparable to in vivo methods while potentially enhancing ecological validity, as exposure takes place within the patient’s real-world environment (Dünser et al. 2011; Elphinston et al. 2023; Suso-Ribera et al. 2019). Taken together, these findings suggest that exposure-based therapy using virtual stimuli represents a viable complement to traditional exposure-based therapy, though further research is needed to optimise its application across different clinical contexts and patient populations.

### Mechanisms of emotion induction in virtual environments

To use VR and AR in exposure-based therapy, it is necessary to understand how emotional responses are triggered in these environments. Appraisal theory suggests that emotions in virtual settings arise from how users evaluate their virtual circumstances, specifically in terms of personal relevance and perceived threat (Somarathna et al. 2023). Emotional Processing Theory (Foa and Kozak 1986) further suggests that effective exposure requires the activation of fear structures. Immersive technologies support this by presenting multimodal sensory cues and limiting natural avoidance behaviours, which helps to induce initial emotional and physiological responses (Rizzo & Shilling, 2017). By mirroring the sensory aspects of real threats, virtual environments can provide an embodied experience in which users process both the physical and psychological effects of the stimuli (Spytska 2024).

Objective technological immersion, such as display resolution or stereoscopic 3D rendering, does not solely determine the intensity of emotional responses. While immersive 3D environments require the allocation of more sensory resources and can elicit higher emotional arousal than 2D displays (Tian et al. 2021), the influence of immersion on emotional arousal largely depends on the subjective sense of presence experienced by users. Recent studies suggest that spatial presence and social presence act as mediating factors that translate system immersion into subjective emotional arousal (Pavic et al. 2023). This mechanism is explained by the presence as a gateway hypothesis, which suggests that a specific degree of presence is necessary to provoke emotional responses in virtual environments (Felnhofer et al., 2015). This indicates that the feeling of *being there* functions as a psychological bridge between hardware characteristics and actual emotional experience. In addition, emotion elicitation and presence appear to interact with each other. Studies indicate an interaction where anxiety and presence mutually reinforce each other (Bouchard et al. 2008; Riva et al. 2007).

Experimental studies show that experiencing fear in a virtual environment directly enhances the feeling of being there for the user, while interpersonal variability in presence predicts the strength of subsequent fear responses (Gromer et al. 2019). When instinctive emotional states are integrated by the brain, they develop into consciously experienced fear. This generates physiological feedback that further strengthens the subjective sense of presence.

According to the affective congruence model, emotional responses that are consistent with the modelled situation enhance the perceived realism of the experience (Robillard et al. 2003). Chirico and Gaggioli (2019) found that emotional responses and presence in immersive virtual environments can be comparable to those in real-life scenarios, and that the affective design of the scenario matters more than its technical features. This is further supported by Rivu et al. (2021), who found that virtual environments produce emotional states and arousal levels comparable to those elicited by identical stimuli in physical environments. For example, moving stimuli such as spiders tend to elicit stronger emotional responses than static ones, and the nature of the stimulus is related to the level of interaction required (De Witte et al. 2020). Scenarios that include contextually appropriate and multimodal stimuli are therefore more likely to produce meaningful emotional engagement, which may explain why the effectiveness of immersive technologies varies depending on the type of stimulus presented.

Research suggests that non-clinical participants can produce genuine affective responses to virtual stimuli. Yeh et al. (2018) found that both AR and VR induced measurable anxiety in healthy participants across subjective scales and physiological indicators, while a related study by the same research group, Tsai et al. (2018) similarly observed comparable physiological arousal using heart rate variability. Robillard et al. (2003) further found that anxiety could be induced in virtual environments in non-phobic controls as well as phobic participants, and that presence and anxiety showed a synergistic relationship across both groups. These findings suggest that the mechanisms linking presence and emotional activation in virtual environments are not exclusive to clinical populations, and that non-clinical samples may offer a reasonable starting point for evaluating the affective properties of immersive technologies prior to clinical deployment, although the link between fear and sense of presence suggests that responses may be stronger in clinical populations.

### Comparison of VR and AR in exposure-based therapy

ET-VR and ET-AR show distinct technical characteristics that may affect their relative effectiveness in different exposure contexts. General comparative studies suggest that VR tends to produce higher levels of immersion and perceived realism, while AR conditions have been associated with greater physiological arousal and interactivity (Antoniou et al. 2020; Chicchi Giglioli et al. 2019; Guo et al. 2024). AR’s interactivity has also been found to have a stronger direct effect on presence than VR, and the presence of the real environment in AR may influence how virtual objects are perceived, for example making them feel physically softer than equivalent objects in VR (Gaffary et al. 2017; Guo et al. 2024).

When applied to exposure contexts, these characteristics suggest distinct strengths for each technology. VR’s high level of sensory immersion and complete environmental control may make it effective for scenarios that are extreme or highly specific. AR, by contrast, provides a more naturalistic exposure experience by allowing users to see their own bodies interacting with virtual elements within their current real surroundings, which may be better suited for scenarios requiring gradual integration of aversive stimuli into everyday contexts. These distinctions suggest that their relative effectiveness may vary depending on the specific nature of the stimulus and the goals of the exposure.

Although some comparative studies of AR and VR in exposure-based therapy exist, they remain limited in scope. Prior work has compared the two technologies within single stimulus conditions, including claustrophobia (Tsai et al. 2018; Yeh et al. 2018) and acrophobia (Juan and Pérez 2010), and found no significant difference in induced anxiety between the two modalities. However, these findings cannot be assumed to generalise across stimulus types. As discussed in Sect.  2.3, the affective congruence model and related work suggest that the effectiveness of a given medium depends on the fit between its technical properties and the characteristics of the stimulus presented, including its spatial, dynamic, and interactivity demands. A stimulus that places similar demands on both AR and VR, such as a spatially enclosed environment, may produce comparable responses regardless of modality. VR’s fully enclosed environment may be better suited to stimuli where surrounding context is central to the aversive response, such as contamination scenarios that rely on a coherent dirty environment to elicit discomfort (Fajnerová et al. 2023; Lohse et al. 2023). In contrast, AR’s integration of virtual elements into real space may offer more ecological validity for stimuli experienced as intrusions into the immediate environment, such as spider stimuli, where the real-world backdrop and the visibility of the user’s own body contribute to perceived threat (Juan and Pérez 2010; Jurcik et al. 2024). These distinctions suggest that the relative effectiveness of VR and AR is unlikely to be uniform across stimulus types, and that conclusions drawn from studies conducted within a single exposure context cannot be assumed to generalise to scenarios with different spatial, dynamic, or contextual properties.

A theoretically motivated research gap therefore exists: it remains unknown whether VR and AR differ in their capacity to elicit emotional responses when the nature of the modelled stimulus changes, and whether one technology may be more effective than the other for specific exposure scenarios. To address this, the study directly compares VR and AR across two qualitatively distinct stimuli, spiders and contamination, which differ in their spatial, dynamic, and contextual properties, and for which both technologies have been independently applied in prior exposure research.

## Methodology

The research focused on two key questions: whether one technology (AR or VR) generally induces a stronger experience intensity (Q1), and whether technology-related differences in experience intensity depend on the characteristics of the modelled stimulus (Q2). Hypotheses were formulated for these questions, and to test them, a within-subjects experiment was designed. Four integrated prototypes were developed, allowing participants to experience virtual spider and contamination scenarios in both AR and VR environments. Participants engaged in a series of tasks, and their self-reported emotional and experiential responses were measured using both quantitative (questionnaires) and qualitative (think-aloud protocols and follow-up interview) methods for subsequent hypothesis testing and qualitative analysis.

### Hypotheses

Based on the above questions, the following hypotheses were proposed:

#### H1. Main Effect of Technology Type (AR vs. VR)

It is hypothesised that there will be a significant difference in the experience intensity induced by AR and VR technologies.

#### H2. Interaction Effect Between Technology Type and Stimulus Type

It is hypothesised that the experience intensity of AR versus VR will vary depending on the specific stimulus type (spider vs. contamination). This hypothesis addresses research question Q2. A significant interaction effect would suggest that the relative advantages of AR and VR may depend on the specific stimulus type.

### Study design

To address the research questions, this study employed a within-subjects experimental design within an explanatory mixed-methods framework, in which qualitative data were used to interpret and contextualise the quantitative findings. Each participant experienced both AR and VR environments for both stimulus types.

The experimental design included two independent variables (IVs) and one main dependent variable (DV) as follows:

- **Factor 1 (A)**: independent variable 1 (IV1) pertains to technology, with two levels: AR and VR.
- **Factor 2 (B)**: independent variable 2 (IV2) concerns the type of stimulus, also with two levels: spider and contamination.
- **Dependent Variable (DV)**: experience intensity, evaluated quantitatively through questionnaire scales.

All hypotheses were tested quantitatively. Qualitative data from think-aloud protocols and follow-up interviews were used in an explanatory and complementary role to interpret the primary outcomes and explain why the contamination and spider scenarios produced different patterns across AR and VR.

Participants engaged in a series of tasks in both AR and VR environments for each stimulus type (spider and contamination), experiencing in total four experimental conditions and reporting the experience intensity for each. The order of conditions was balanced across participants to compensate for learning and fatigue effects. The two stimulus conditions were matched in terms of task structure, interaction modality, task duration, and questionnaire format. Differences in the spatial and contextual properties of the two stimuli were intentional, reflecting the study’s aim to examine whether stimulus type moderates the experience intensity of AR and VR.

### Prototype design

A combined prototype was developed for AR and VR spider and contamination scenarios. These prototype scenarios were designed based on previous studies and designs, and are described below (see Online Resource 1 for example video).

#### Hardware and development engine

The Meta Quest 3 was employed as the primary hardware device for the study. Meta Quest 3 is a VR headset, released in 2023, that offers high-resolution (2064 × 2208) displays with approximately 110° field of view and hand tracking. It also includes a colour video passthrough function, enabling simple switching between AR and VR modes. This feature allows for the effective integration of both environments within the prototype.

The VR and AR scenarios were developed using the Unity^®^ Pro game engine^1^. The Unity engine is a widely adopted platform, compatible with the Meta Quest 3, including support for hand tracking and other relevant features, making it well-suited for the development of this study. Additionally, Unity’s extensive asset store and flexible scripting environment facilitated the prototyping and iteration necessary to deliver the study.

#### Hand tracking technology

The Quest 3 allows users to interact with the virtual or augmented environment either using hand-held controllers or by directly tracking the user’s hands in space. In the prototype hand tracking was used in preference to handheld controllers for the following reasons. Firstly, hand tracking may increase the sense of direct contact and everyday interactions between participants and fear-inducing stimuli (such as spiders or contaminated objects). In fear treatment, realistically simulating everyday scenarios is a strategy for helping participants overcome their fears. Hand tracking may more effectively evoke emotional responses in participants, thereby enhancing the impact of exposure-based therapy. Secondly, hand tracking technology can reduce the sense of artificiality and game-like feel associated with handheld controllers. In virtual environments, the use of controllers can reinforce a ‘gaming mindset’ in participants. Even if the virtual scenario is designed to be highly realistic, the presence of controllers may remind participants that they are in a virtual world, thus diminishing the sense of immersion. By using hand tracking, participants can interact directly using their own hands. This not only enhances the naturalness of interactions but may also increase the immersive experience of the scenario, blurring the boundary between virtual and real, and making it easier for participants to focus on the current therapeutic task.

#### User interface and scenarios design

The interactive prototype was designed and developed comprising four distinct scenarios: an AR virtual spider, a VR virtual spider, an AR contamination scenario, and a VR contamination scenario. These four scenarios were integrated within a single Unity project ‘VR/AR-ET Comparison Trial.’ Users were guided through different scenarios within the prototype and could interact with virtual models in each scenario.

**Fig. 1:**
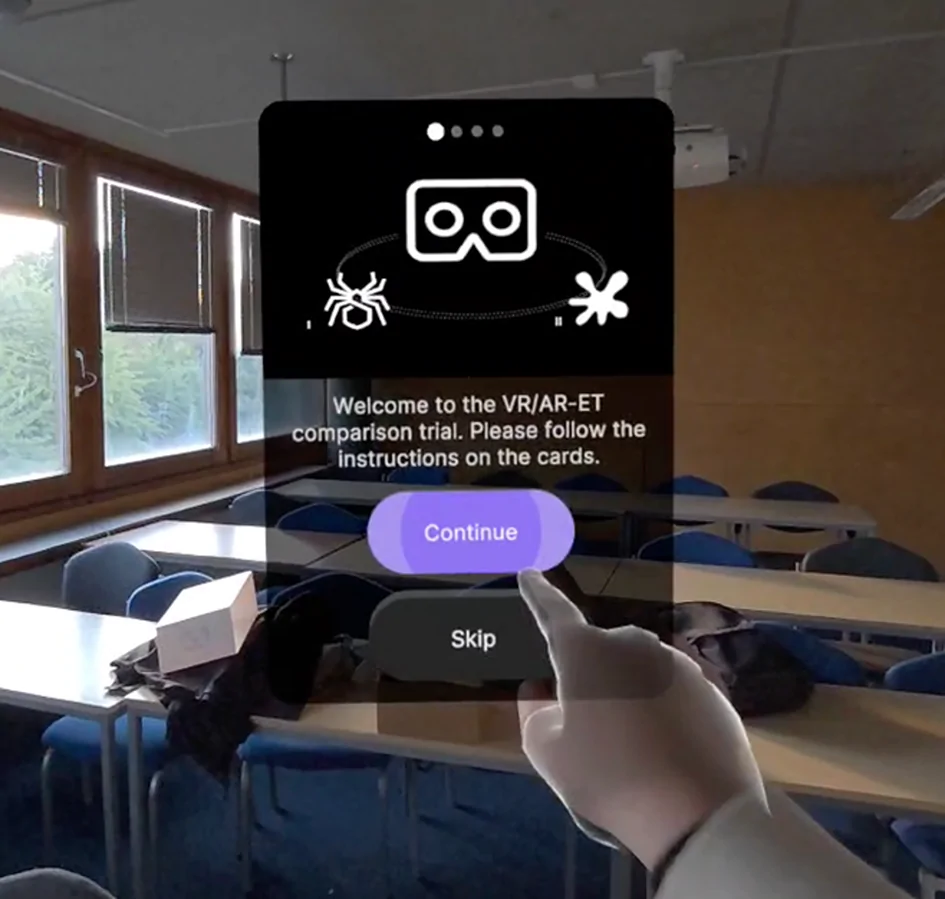
Instructions Start Page (example headset view)

**User Interface (UI)**:

Instruction cards were provided to introduce and initiate the prototype (see Fig. 1).

**Fig. 2:**
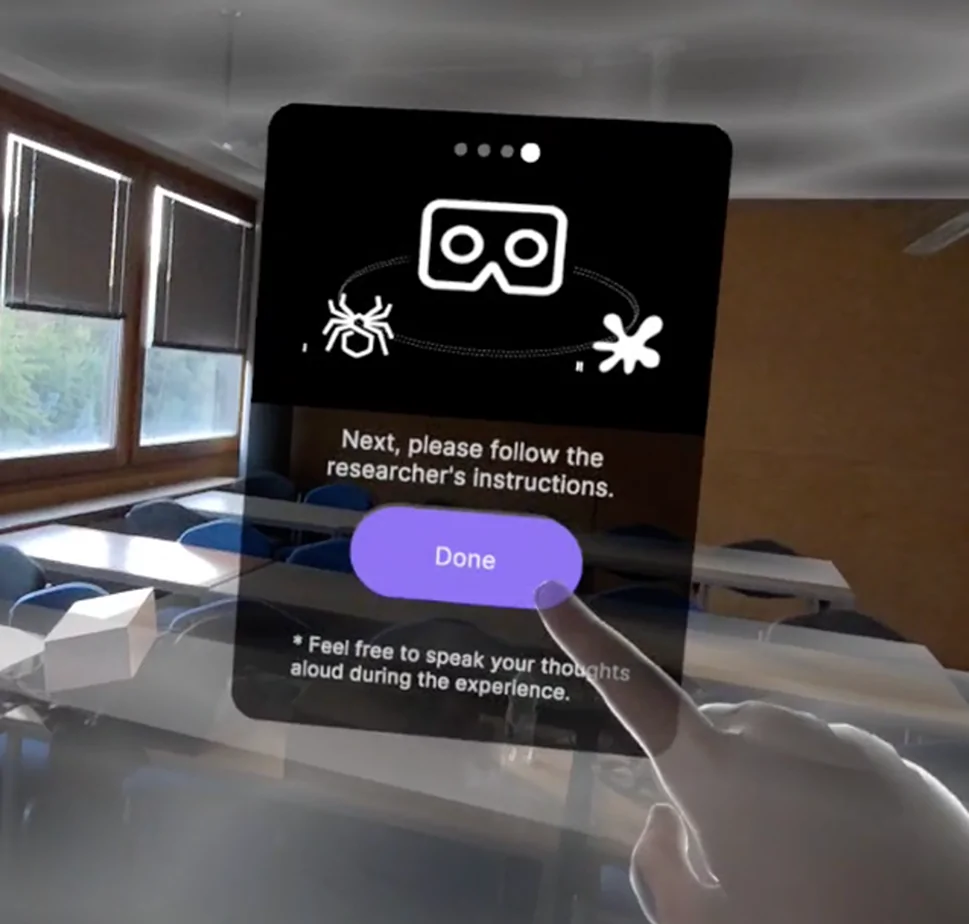
Instructions End Page (example headset view)

The design also integrated verbal cues from the researcher, as would be encountered in real-world applications with a therapist (see Fig. 2). Participants were encouraged to use a think-aloud method during the experimental process, i.e. to describe to the researcher what they were doing and thinking.

**Fig. 3:**
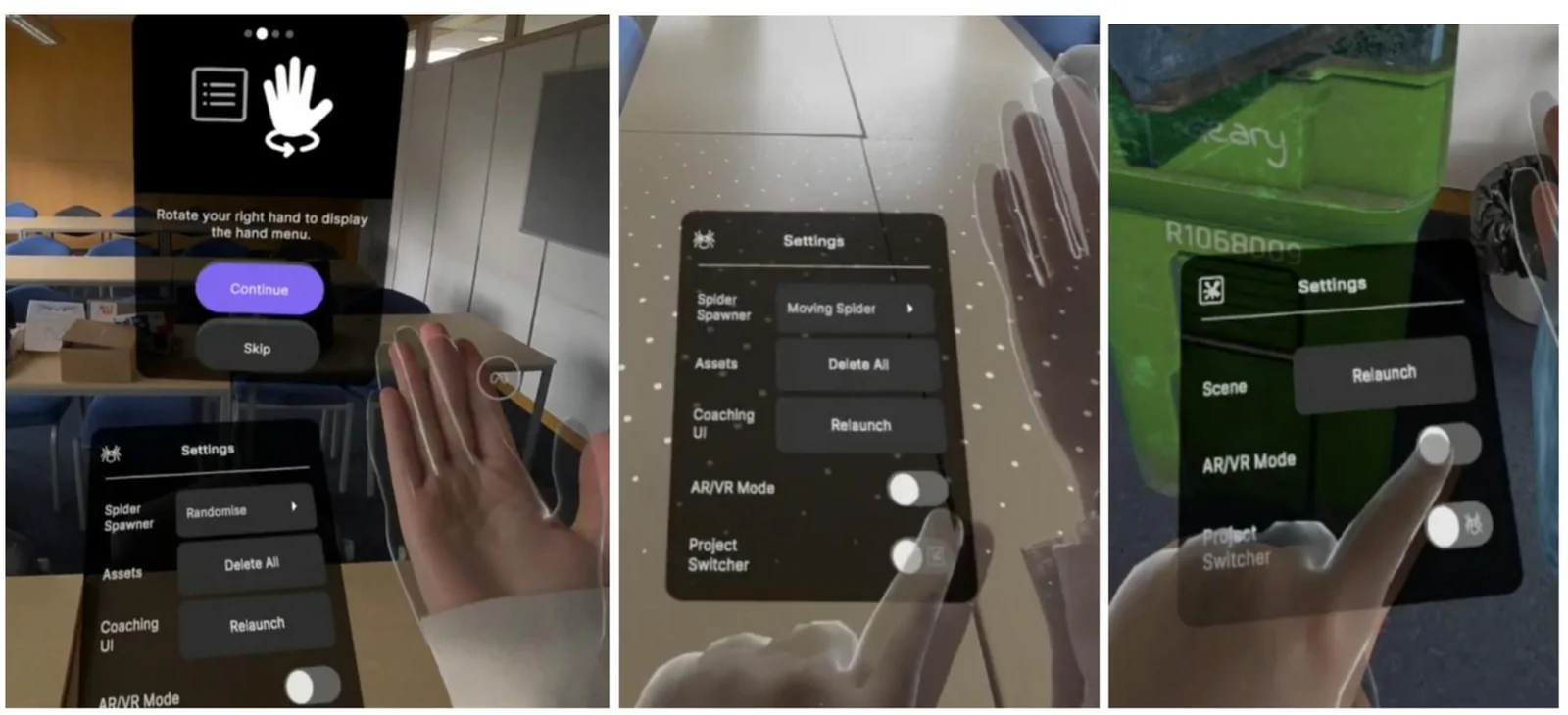
Setting Menu Interfaces (example headset views) (**a**) accessing settings (**b**) changing scenario (**c**) changing mode

Users could navigate and control various scenes and settings through a menu that was activated by rotating their right hand (see Fig. 3).

**Stimulus Scenarios**:

Two distinct stimulus scenarios were developed.

**Scenario 1** focused on exposure to spiders, involving interactive virtual spiders.

According to a study on individuals with spider phobia, moving animals increase the sense of fear and the effectiveness of exposure-based therapy compared to static ones (Lindner et al. 2019). Therefore, the spider model used in this scenario was designed to have both static and moving states. While static, the spider exhibited small ‘idle’ animations. In its moving state, the spider rapidly crawled forward, with leg animations. The moving spider could be activated by interacting with the static spider. Specifically, when the user touched or picked up the static spider it triggered the moving state, simulating the typical behaviour of spiders in real life.

**Fig. 4:**
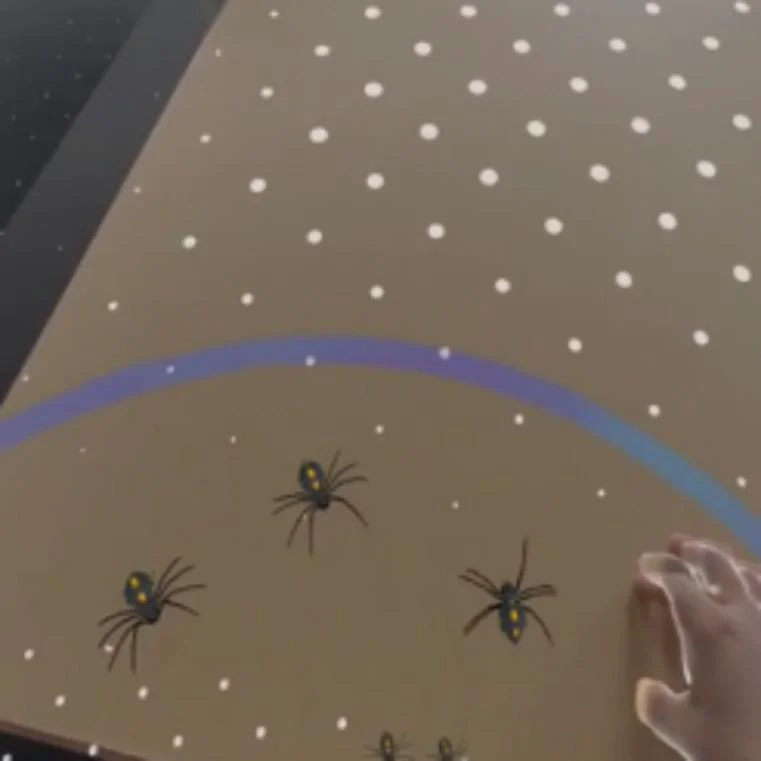
Virtual Spiders in AR (example headset view)

In the **AR** mode, the virtual spider was superimposed onto the real-world environment, such as a table (see Fig. 4). Users could select a suitable flat surface within their physical surroundings to serve as the area for AR spiders to appear. The spider model could be generated at any location on the identified surface, allowing users to set up the AR exposure scenario according to their specific real-world context.

**Fig. 5:**
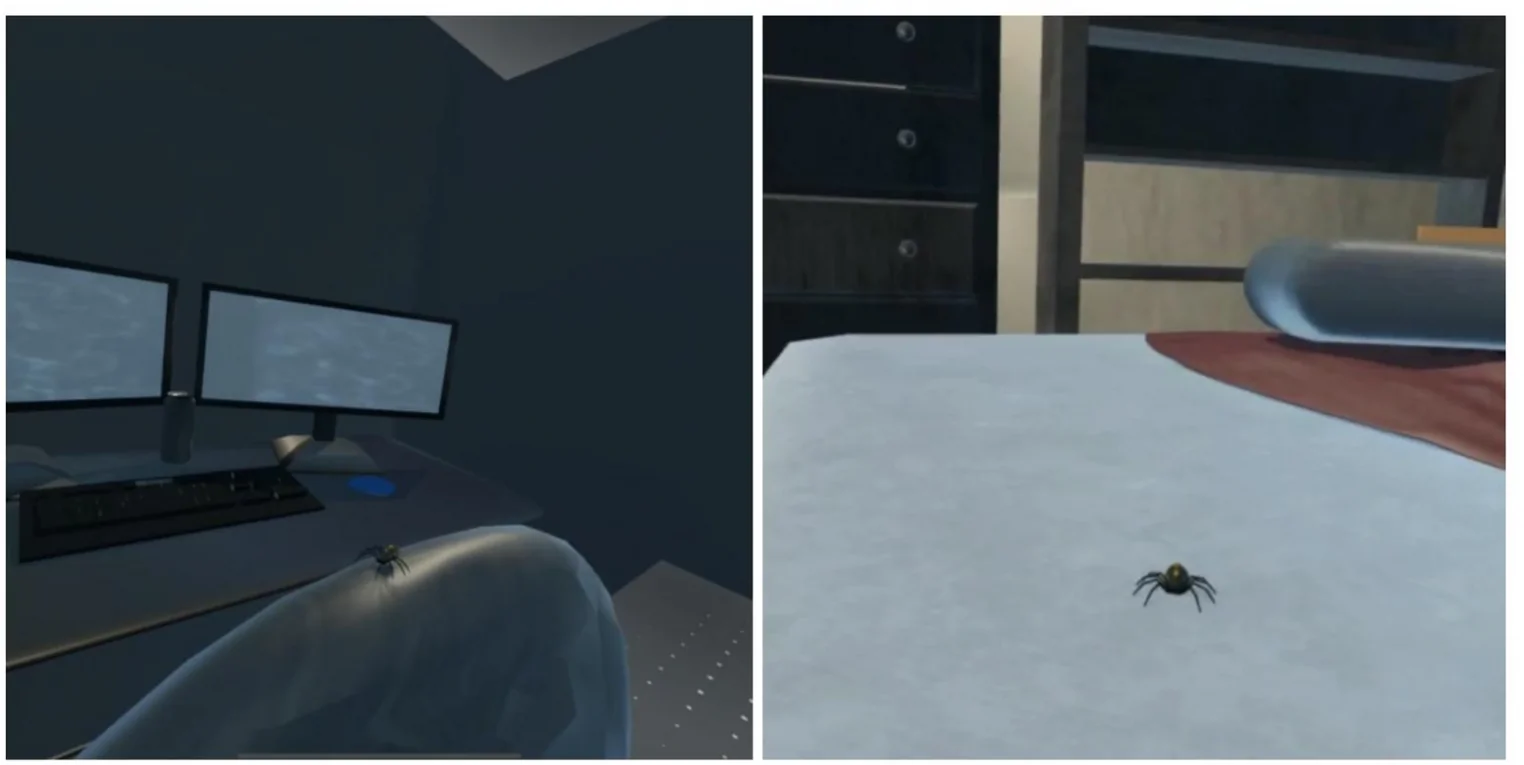
Virtual Spiders in VR (example headset view)

In the **VR** mode, a bedroom environment was modelled, featuring a bed, a computer desk, and a chair (see Fig. 5). Two initially static spiders were pre-placed within this setting to model the experience of encountering spiders in everyday environments.

**Scenario 2** focused on exposure to contamination.

This scenario was designed to depict scattered rubbish and a dirty rubbish bin. Various types of 3D models of rubbish were used, including decaying fruit, cans, and plastic bottles, with a vomit model placed beneath the rubbish. The rubbish could be picked up and moved, and the lid of the rubbish bin and the rubbish bag inside could also be manipulated.

**Fig. 6:**
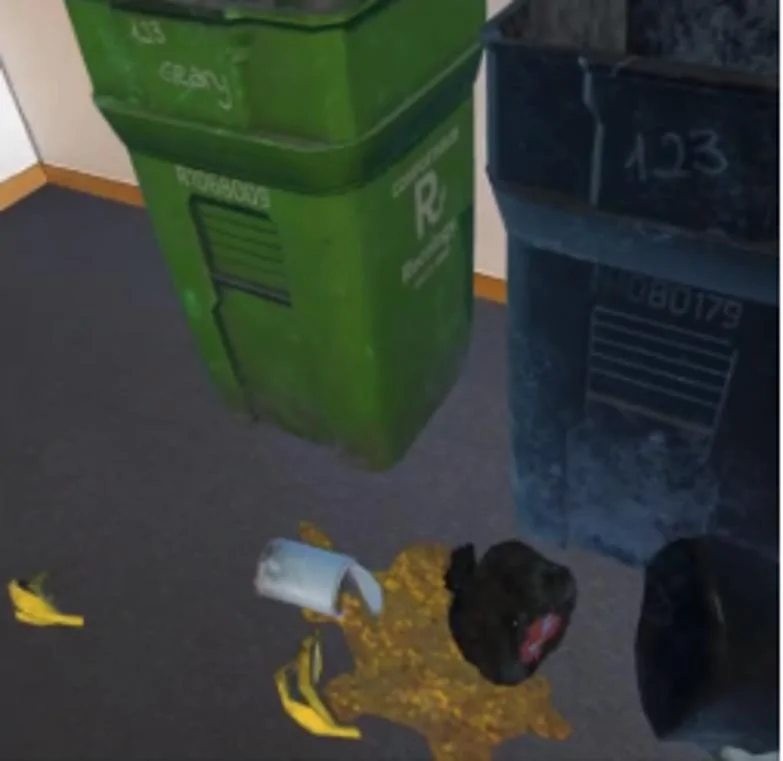
Contamination Environment in AR (example headset view)

In the AR mode, the modelled rubbish and rubbish bin were superimposed onto the real-world environment, within a customisable generation area (see Fig. 6).

**Fig. 7:**
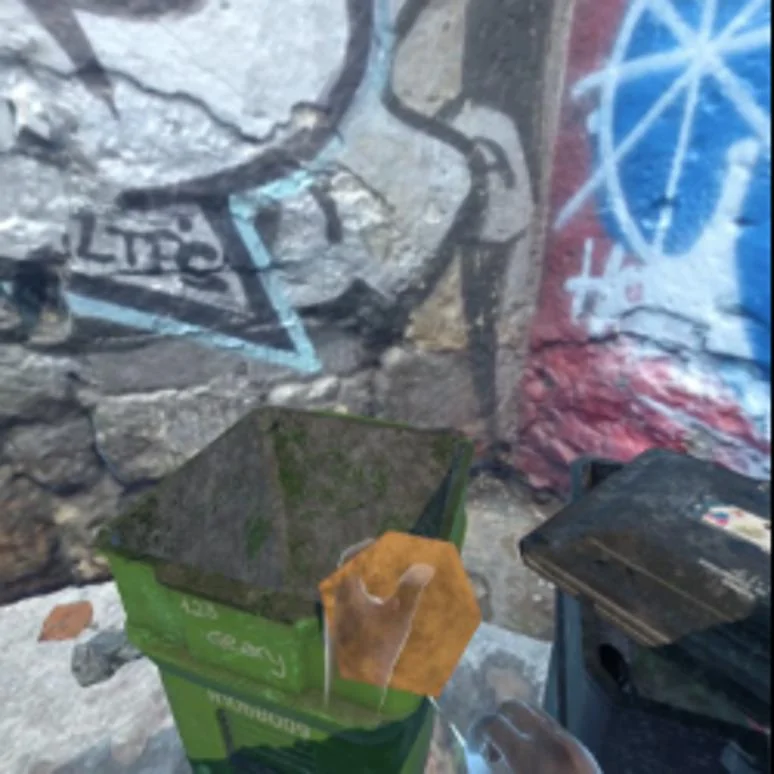
Contamination Environment in VR (example headset view)

The VR mode added a surrounding virtual environment that modelled a cluttered street corner, featuring graffiti-covered walls and a dirty ground (see Fig. 7). The rubbish and rubbish bin were presented within this environment, simulating a consistent setting.

### Experimental procedure

**Fig. 8:**
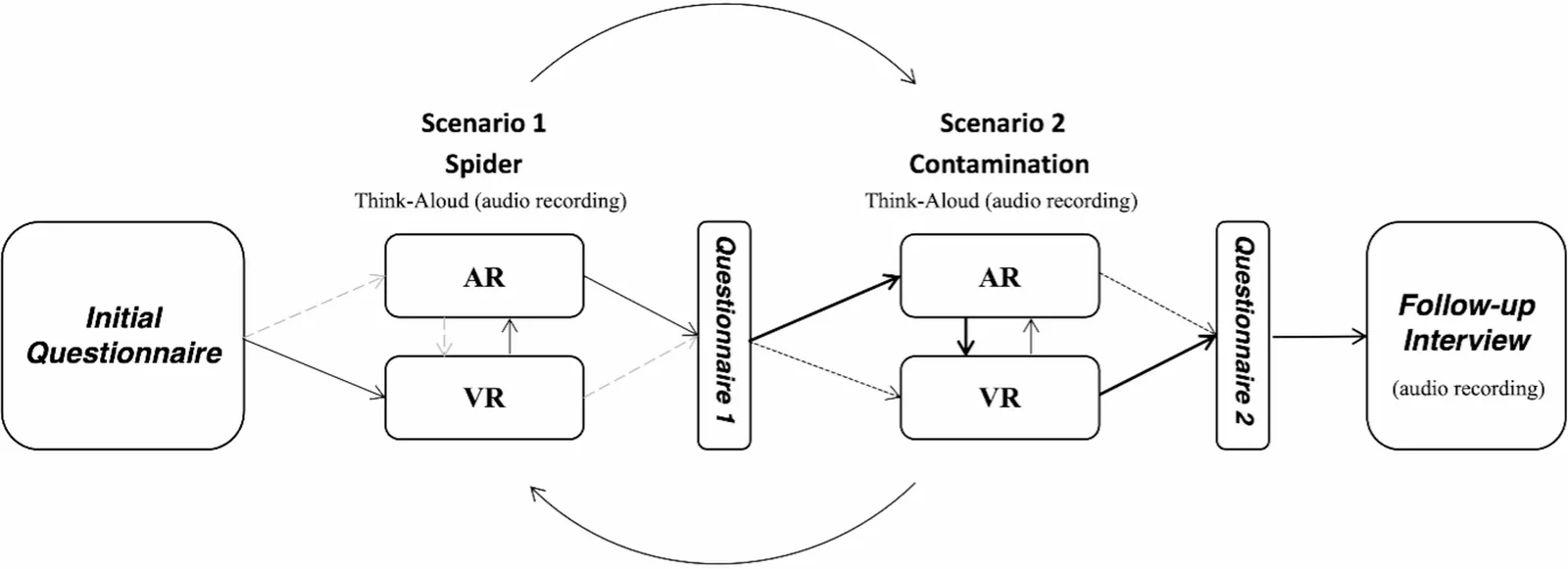
Diagrammatic overview of the experimental procedure (left-to-right)

The experiment procedure (see Fig. 8) was divided into two sections, each corresponding to a different exposure theme: spider and contamination. Each experiment started with an initial questionnaire and was conducted in both AR and VR environments. After completing a section, participants filled out the corresponding part of the experience intensity questionnaire. Once everything was concluded, a follow-up interview was conducted to gather deeper insights into participants’ experiences with VR and AR. A think-aloud protocol was also employed to gather qualitative insights: as participants engaged with the various scenarios, they were encouraged to verbalise their thoughts, feelings, and reactions in real-time. Participant feedback from interviews and think-aloud protocol were recorded during the sessions, then transcribed for analysis to provide context to the quantitative results.

To mitigate the potential influence of the order in which technologies or tasks were experienced on participants’ perceptions and experiences, the sequence of tasks and technology interactions was randomised. The experiment took around 40 min per participant.

The following is a representative example of one such randomised sequence of procedures and includes all of the conditions and tasks:

Step 1: Initial Questionnaire.

- At the start of the study, participants complete an initial questionnaire that includes basic demographic information such as age and gender, questions about their experiences with AR and VR technologies, and items that assess their levels of fear towards spiders and contaminants (see appendix A).

#### Scenario 1

Spider.

**Fig. 9:**
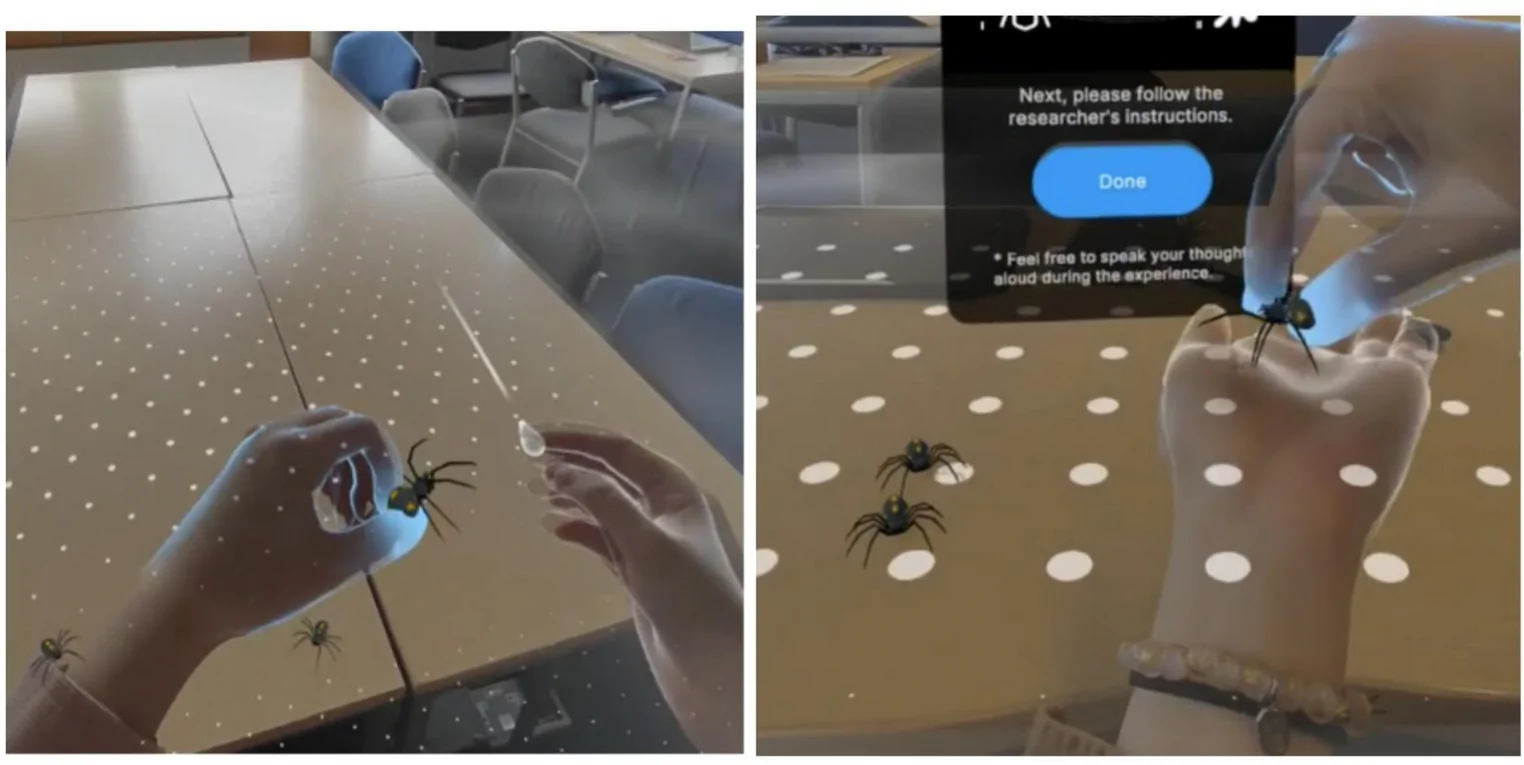
Tasks with Virtual Spiders in AR (example headset views) (**a**) picking up spider (**b**) putting it on your hand

Step 2: AR Tasks (see Fig. 9).

- **Task 1**: **Observing a static virtual spider.** The participant wears a headset to see a static virtual spider appear in their actual surroundings and is guided to carefully observe the spider’s appearance and behaviour.
- **Task 2: Picking up a virtual spider**. The participant reaches out and “grasps” the static virtual spider by pinching their finger and thumb together, activating an animation that models the spider’s movement.
- **Task 3**: **Observing a moving virtual spider.** The participant watches the spider move within the AR environment.
- **Task 4**: **Increased interaction with the spider.** The participant engages in further interactive actions with the moving virtual spider, such as placing the spider on their hand or moving its position.

**Fig. 10:**
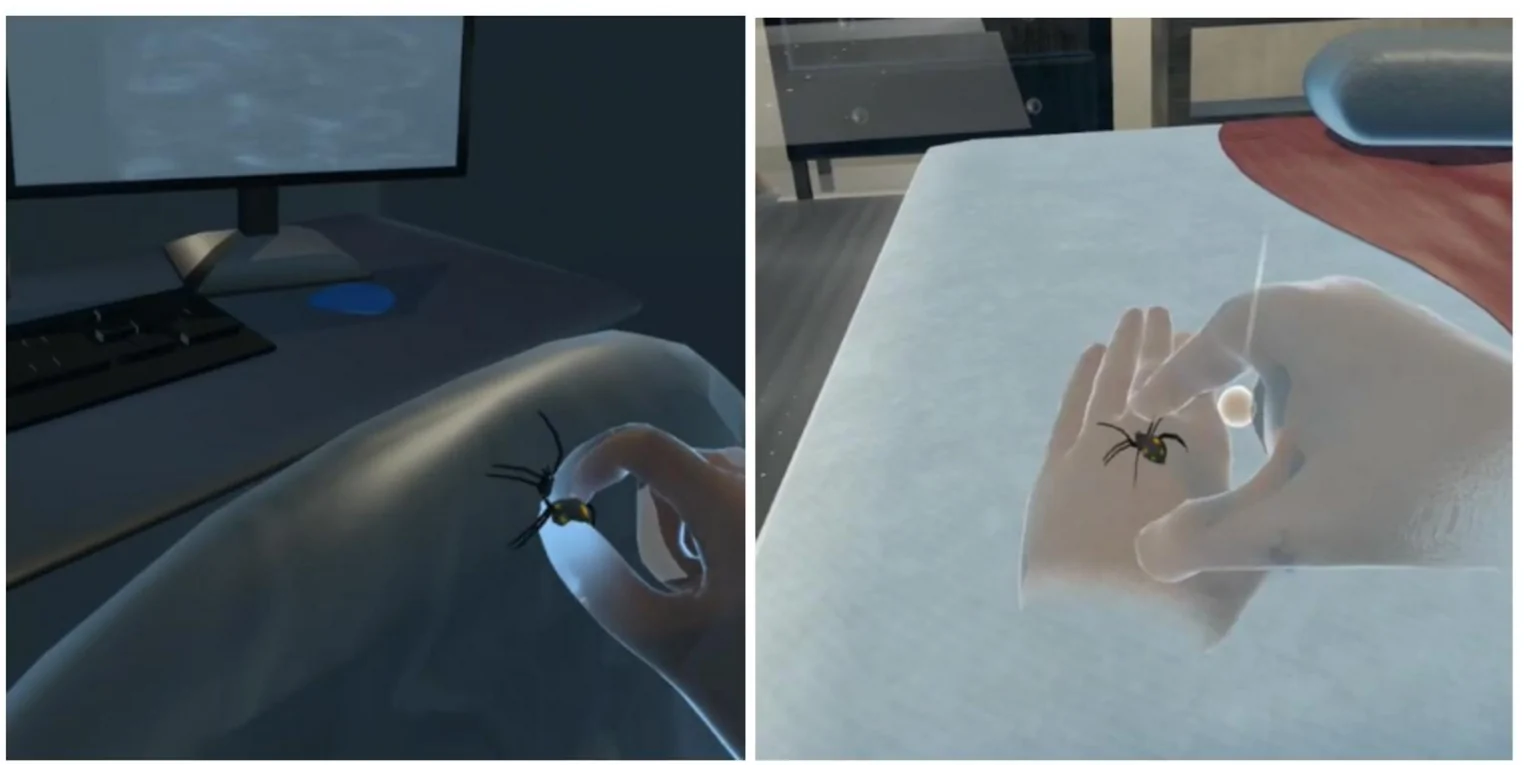
Tasks with Virtual Spiders in VR (example headset view) (**a**) picking up spider (**b**) putting it on our hands

Step 3: VR Tasks (see Fig. 10).

- The participant switches to a VR mode, repeating the tasks of observing, grabbing the static spider, observing the moving spider, and interacting more with the spider.

Step 4: Scenario 1 Questionnaire.

- After completing all of the tasks in the AR spider and VR spider conditions, the participant completes a questionnaire to assess their emotional responses and their perceptions of the AR and VR environments (see Sect.  3.5 and appendix A).

#### Scenario 2

Contamination.

**Fig. 11:**
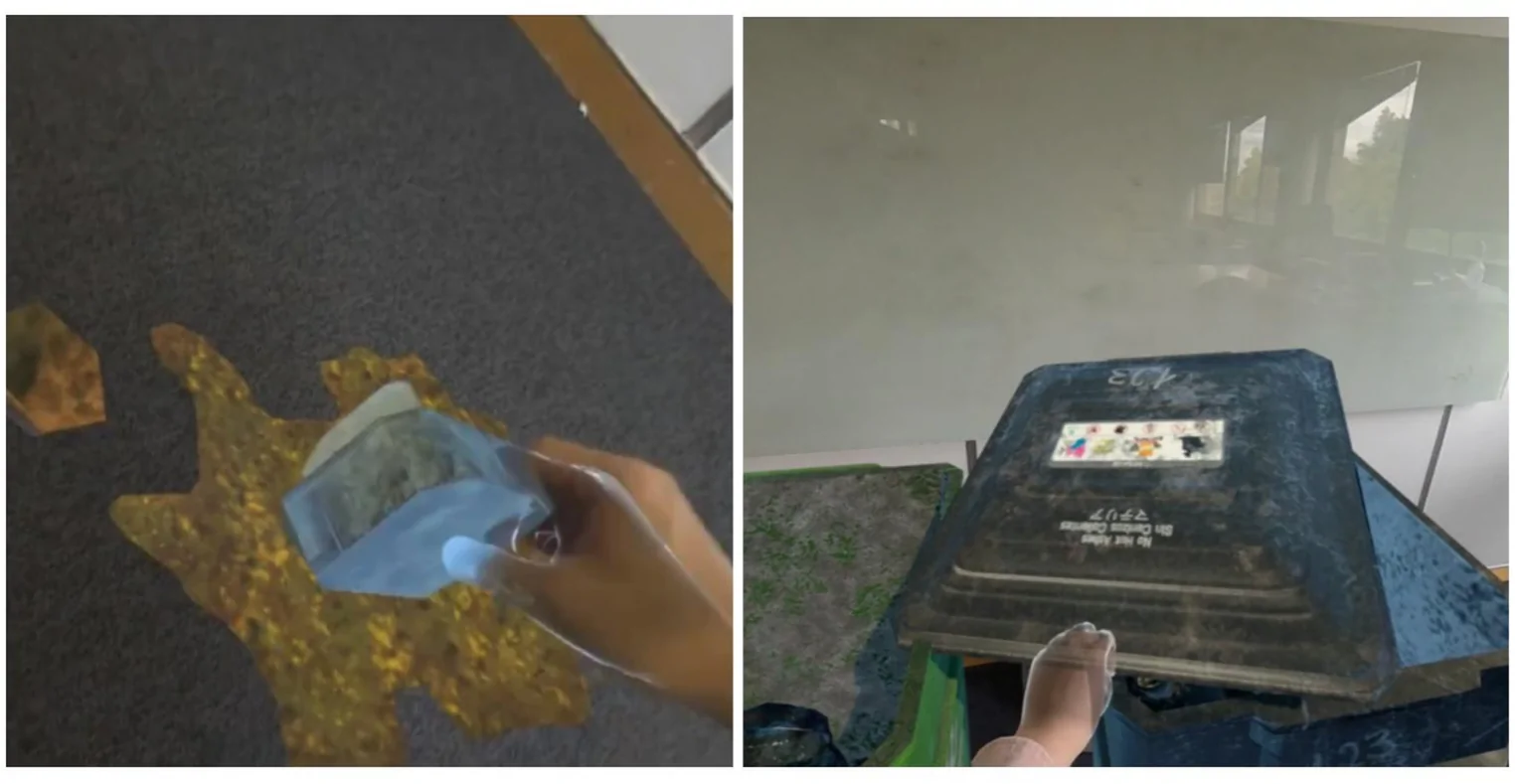
Tasks with Contamination Environment in AR (example headset view) (**a**) picking up rubbish (**b**) lifting bin lid

Step 5: AR Tasks (see Fig. 11).

- **Task 1**: **Observing rubbish on the ground.** In the AR environment, the participant sees a rubbish bin and litter scattered around it and is guided to observe these items.
- **Task 2**: **Picking up rubbish.** The participant “picks up” virtual rubbish by reaching out to it and pinching their finger and thumb together.
- **Task 3**: **Throwing rubbish into the bin.** The participant “places” the picked-up rubbish into an open green bin on their left, simulating the act of disposing of rubbish in real life.
- **Task 4**: **Lifting the bin lid and observing.** The participant lifts the lid of a black bin on their right, observes the interior of the bin, and repeats the act of disposing of ground rubbish, simulating a deeper exposure to contamination.

**Fig. 12:**
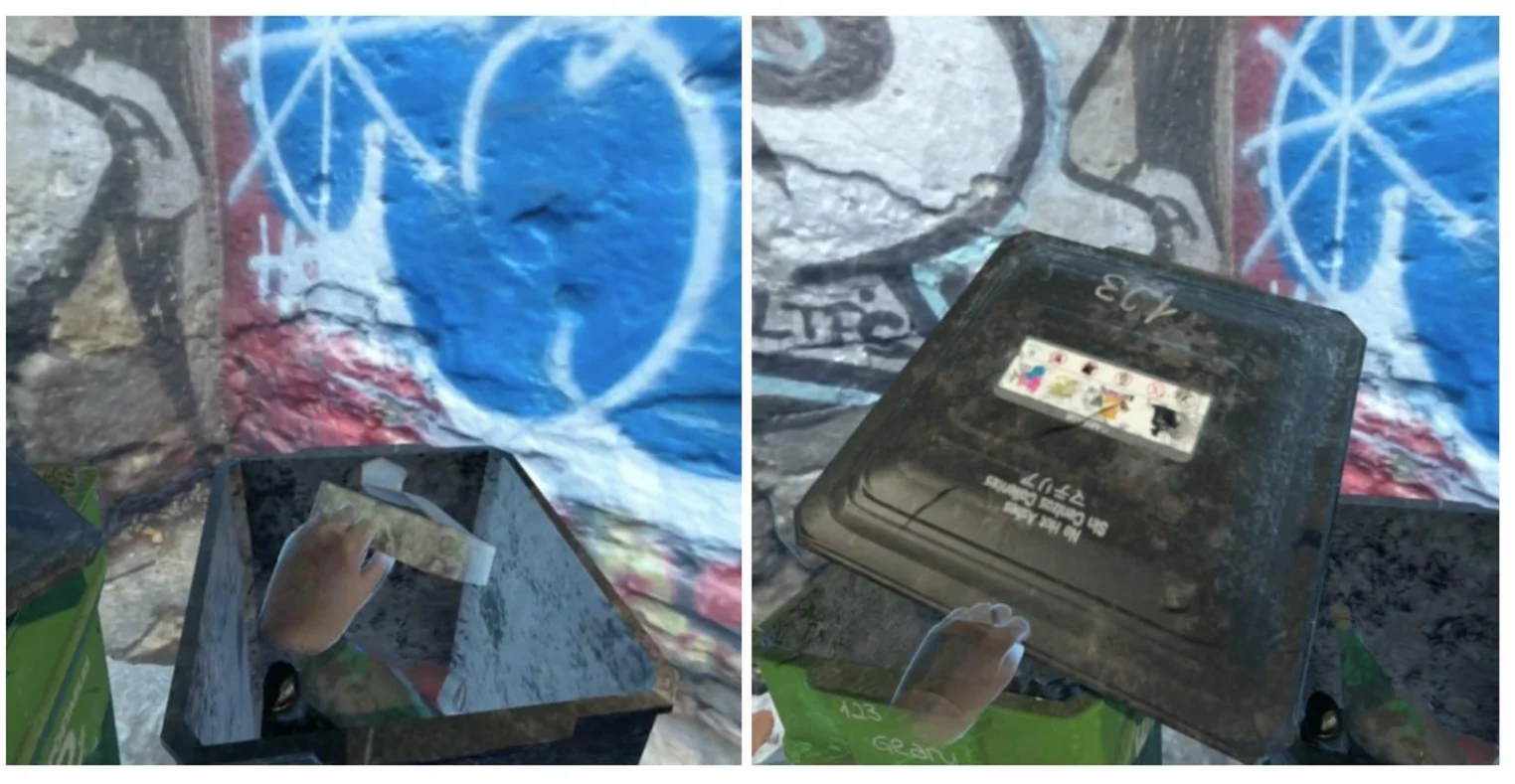
Tasks with Contamination Environment in VR (example headset view) (**a**) holding rubbish (**b**) lifting bin lid

Step 6: VR Tasks (see Fig. 12).

- The tasks are the same as those in the AR setting. Participants switch to a fully virtual setting and repeat the corresponding tasks, performing the same tasks in a VR environment.

Step 7: Scenario 2 Questionnaire.

- After finishing the contamination tasks in both AR and VR settings, the participant fills out a questionnaire to assess their emotional reactions to contamination exposure and their perceptions of the VR and AR environments (see Sect.  3.5 and appendix A).

Step 8: Follow-up Interview.

- After the scenarios, a semi-structured interview is conducted to further explore the participant’s subjective experiences in AR and VR settings. The interview aims to supplement the quantitative data, providing deeper insights into their emotional responses, levels of fear, and personal judgements on both forms of exposure.

### Experience intensity questionnaire

The questionnaires completed after each scenario were designed to assess an overall measure of **experience intensity**. This construct was operationalised through three measurement dimensions: (1) **realism and immersion**, including perceived realism of virtual objects, sensory feedback, and psychological resistance; (2) **behavioural and spatial presence**, reflected in avoidance behaviours when interacting with the virtual stimuli; and (3) **emotional intensity**, capturing the affective response to the virtual stimuli, including fear, anxiety and discomfort – see appendix A.

The questionnaire design integrates elements from several established scales while adapting them to fit the specific needs of this study. It incorporates items from the Immersive Tendency Questionnaire (ITQ) (Witmer and Singer 1998) and MEC Spatial Presence Questionnaire (MEC-SPQ) (Vorderer et al. 2004) to measure experience intensity, along with the Fear of Spiders Questionnaire (FSQ) (Szymanski and O’Donohue 1995) as a reference for emotional and behavioural responses. The final measure comprises 7 items for each phobia scenario, to evaluate participants’ subjective experiences while limiting assessment time, to avoid excessively diverting their attention or disrupting immersion (Bouchard et al. 2008).

The realism and immersion questions are primarily based on the ITQ. Given the experimental prototype design, it tests only visual and haptic sensory modalities. Among the five main factor categories in ITQ, it focuses on sensory and realism factors to align with the research needs regarding experience intensity. The behavioural questions draw from the Spatial Presence questionnaire, examining whether participants feel they “are in the middle of the action rather than merely observing.” This section also incorporates concepts from the FSQ, exploring participants’ aversion reactions, such as their propensity to avoid a spider. Questions concerning emotional response (fear, anxiety) were also adapted from the FSQ, simplified into a single emotional response scale item, and appropriately modified for questions related to emotional responses to contamination items.

The questions were tailored for each scenario but intended to measure the same types of response. The questions use a 7-point Likert scale (1 = Not at all to 7 = Extremely), and the participant provides two responses for each question: a rating for the VR environment and a rating for the AR environment for that scenario. The overall experience intensity is calculated as the sum of the responses to the 7 items for that technology, with a range 7–49.

In addition to rating each technology, after each scenario participants were also asked which technology they would prefer to use for that scenario and which they considered to be more realistic.

### Qualitative data handling

Interviews were conducted by the first author following a semi-structured guide (see appendix B). Recordings were transcribed and anonymised. Reflexive thematic analysis (Braun and Clarke 2006) was applied to the interview transcripts. To minimise researcher bias, the analysis followed an inductive approach in which codes were generated directly from the data without imposing a predetermined framework. Themes were developed iteratively through repeated engagement with the full dataset.

### Participant recruitment

This study recruited participants through voluntary registration. Recruitment was primarily conducted via social media, email, and through distributing posters within student communities at a UK University, targeting eligible individuals. Eligibility criteria were as follows: (1) over the age of 18; (2) no serious psychological disorders or other significant health conditions; (3) normal or corrected vision, enabling them to use AR and VR devices without difficulty.

The study focused on a non-clinical population for two reasons. First, establishing the affective properties of virtual environments in healthy participants represents a standard foundational step before clinical deployment. Second, as the prototype has not yet undergone formal clinical validation, recruiting individuals with clinical anxiety at this stage would raise additional ethical and safety concerns.

### Compliance with ethical standards

The authors have no relevant financial or non-financial interests to disclose. The study was reviewed and received a favourable ethical opinion from the School’s Research Ethics Committee (CS-2023-M14). All participants were briefed (including their right to withdraw), able to ask questions and gave informed consent (in writing) prior to commencing the study.

Exposure to virtual spiders or contamination scenes may cause anxiety, and VR/AR devices may cause physical discomfort such as nausea. To manage these risks, individuals who self-reported a clinical diagnosis of arachnophobia or contamination-related OCD were excluded. All participants provided informed consent and were told before each condition that they could pause or withdraw at any time. The researcher monitored verbal and behavioural cues throughout and was ready to stop the study if participants demonstrated significant distress. A debriefing session was held after the experiment to address any residual distress.

## Results

The results of the experimental study comprise both quantitative self-report measures and qualitative feedback, with the qualitative insights from participants serving as a complement to statistical hypothesis testing. See Online Resource 2 for raw questionnaire responses, Online Resource 3 for anonymised interview transcripts and Online Resources 4 and 5 for the output from statistical tests from SPSS. By analysing the reported experience intensity from 31 participants across different technologies and scenario settings, the findings address the study questions by highlighting the relative experience intensity of VR and AR in different types of exposure scenario.

### Demographic and preliminary analyses

A total of 31 participants took part in the study (20 females, 11 males), aged between 22 and 33 years (*M* = 25.41, *SD* = 2.54).

At the start of the study, participants rated their comfort level with spiders on a 7-point scale (1 = Completely comfortable, 7 = Very scared), with scores ranging from 1 to 7 (*M* = 4.16, *SD* = 1.46). They also rated their level of concern about contaminants in their daily environment on a 7-point scale (1 = Not at all concerned, 7 = Very concerned), with scores ranging from 2 to 7 (*M* = 4.71, *SD* = 1.19).

Familiarity with AR and VR technology was assessed using a three-point scale (never used, limited experience, familiar). Among the participants, 48% (*n* = 15) were familiar with VR and 42% (*n* = 13) with AR, with 35.5% (*n* = 11) reporting familiarity with both. No participants reported never having used VR, whilst three reported never having used AR. The remainder had limited experience.

### Experience intensity

This study used a two-factor analysis of variance (ANOVA) to explore the impact of AR and VR technologies on the experience intensity related to two specific exposure scenarios - spider and contamination. The analysis focused on the main effect of technology type and its interaction with stimulus type. The scenario-specific experience intensity questionnaire comprised seven items. For each condition these have Cronbach’s Alpha values between 0.9 and 0.95 (Table 1), which are acceptable, indicating reliable internal consistency for these scales (Bland and Altman 1997).

**Table 1:** Cronbach’s Alpha Values

Questionnaire	Cronbach’s Alpha
Spider_VR	0.921
Spider_AR	0.904
Contamination_VR	0.930
Contamination_AR	0.942

#### Descriptive statistics

Descriptive statistics for the experience intensity (scale range 7–49) with different technologies and exposure scenarios are shown in Table 2.

**Table 2:** Descriptive Statistics Results

*N* = 31	Condition	Mean	SD
*N* = 31	Spider _ AR	29.68	9.758
	Spider _ VR	30.71	10.143
	Contamination _ AR	26.13	10.941
	Contamination _ VR	33.13	11.215

#### Statistical assumptions

Prior to conducting a two-way repeated measures ANOVA, statistical assumptions were verified. Shapiro-Wilk tests indicated that the data for the Contamination AR condition significantly deviated from normality (*W* = 0.927, *p* = .037), while all other conditions were normally distributed (all *p*s > 0.05). Given the robustness of ANOVA to moderate violations of normality in balanced designs, the analysis proceeded. Mauchly’s test of sphericity was not applicable or met for the 2 × 2 design (df = 1), so no correction to degrees of freedom was necessary.

#### Hypothesis testing

A two-way repeated measures ANOVA was conducted to assess the effects of technology type (AR vs. VR) and stimulus type (spider vs. contamination) on experience intensity.

The results revealed a significant main effect of technology type on experience intensity, *F*_(1, 30)_ = 14.358, *p* < .001, *η*² = 0.324, supporting H1. Overall, VR elicited a significantly higher experience intensity than AR.

A significant interaction effect was observed between technology type and stimulus type, *F*_(1, 30)_ = 10.251, *p* = .003, *η*² = 0.255. This indicates that the impact of the technology type on experience intensity varied depending on the specific stimulus presented, supporting H2.

The stimulus type had no significant effect on experience intensity, F (1, 30) = 0.09, *p* = .766, η² = 0.003, indicating that the spider and contamination conditions produced similar *overall* levels of experience intensity in this study population when averaged across both technology conditions.

#### Post-Hoc analysis of the interaction effect

To further explore the interaction effect, Bonferroni-adjusted paired samples t-tests (*α* = 0.0125) were conducted to compare AR and VR within each stimulus type.

**Fig. 13:**
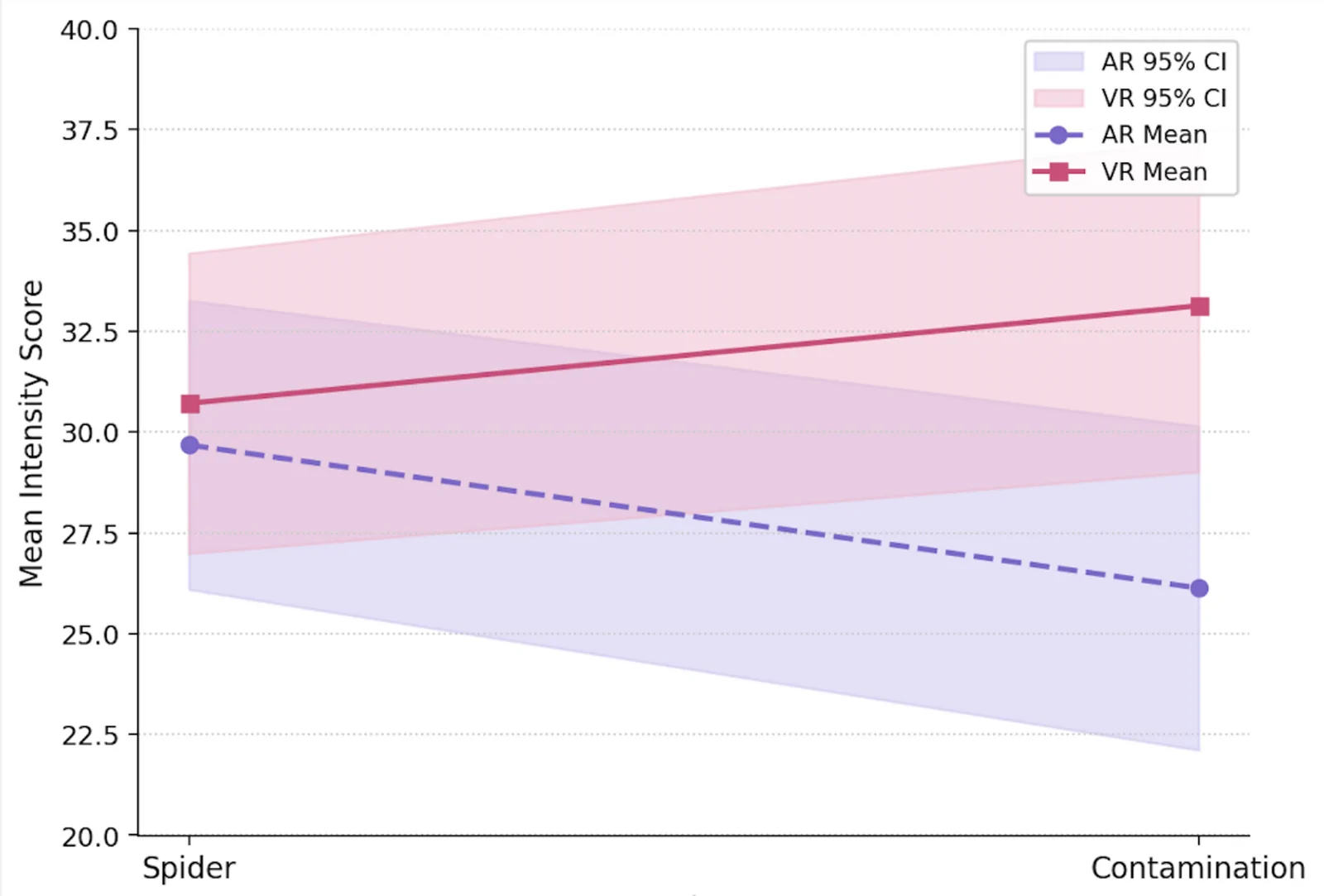
Interaction Effect of Technology and Stimulus Type on Experience Intensity

In the contamination scenario, VR produced a significantly higher experience intensity (*M* = 33.13, *SD* = 11.21) compared to AR (*M* = 26.13, *SD* = 10.94), *t*(30) = − 4.97, *p*<.001. In contrast, for the spider scenario, there was no significant difference in experience intensity between VR (*M* = 30.71, *SD* = 10.14) and AR (*M* = 29.68, *SD* = 9.76), *t*(30) = − 0.73, *p*=.471 (see Fig. 13).

The 95% confidence intervals were as follows: Spider_AR [26.10, 33.26], Spider_VR [26.99, 34.43], Contamination_AR [22.12, 30.14], and Contamination_VR [29.02, 37.24].

#### Individual factors

Spearman rank correlations were conducted to examine whether self-reported aversive sensitivity and prior technology familiarity were associated with the four outcome measures. No significant associations emerged between familiarity and any of the four outcome measures (all |*r*_*s*_| < 0.27, all *p*s > 0.15), indicating that prior experience with AR or VR did not systematically influence experience intensity.

For self-reported aversive sensitivity, spider discomfort was significantly associated with Spider_AR scores (*rs* = 0.559, *p* = .001) but not with Spider_VR scores (*rs* = 0.125, *p* = .502). Contamination concern showed no significant association with either contamination outcome (Contamination_AR: *rs* = 0.238, *p* = .196; Contamination_VR: *rs* = 0.028, *p* = .883). Cross-domain correlations were similarly non-significant (all *rs* < 0.17, all *p*s > 0.36).

### Explicit technology preference

Participants were also asked to rate perceived realism and technology preference, comparing VR and AR.

**Fig. 14:**
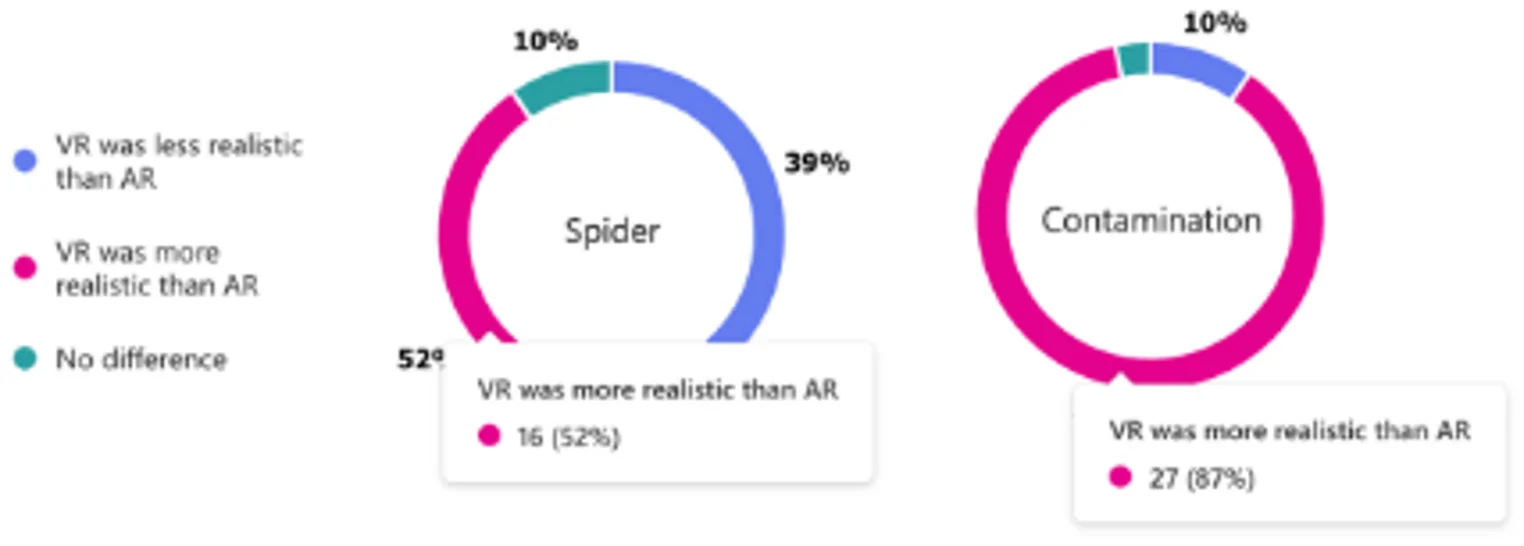
Perceived Realism Results

For perceived realism, a majority of participants identified VR as the more realistic modality across both conditions (52% for the spider scenario and 87% for contamination; see Fig. 14).

**Fig. 15:**
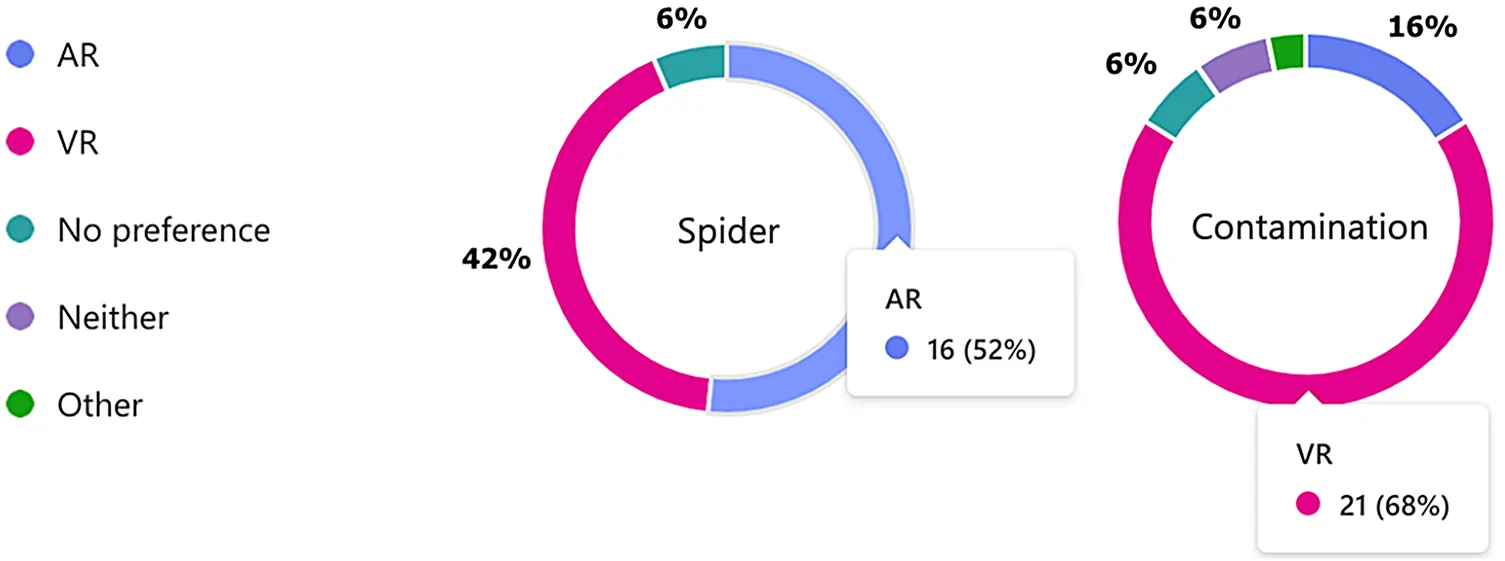
Technology Preference Results

When asked to select a preferred technology for therapeutic purposes, participants showed diverging preferences based on the stimulus type. Specifically, 52% of participants preferred AR for spider exposure, whereas 68% chose VR for contamination exposure (see Fig. 15).

### Qualitative findings

As noted above, reflexive thematic analysis was applied to the interview transcripts from all 31 participants (P1–P31) following their completion of the study sessions. Five themes were identified, illuminating the specific dimensions through which AR and VR produced different experiential effects: environmental coherence and perceived control were more salient in the contamination scenario, while embodiment and bodily presence were more prominent in the spider scenario.

#### Environmental Coherence

Participants’ descriptions of immersion and emotional engagement varied depending on whether the virtual stimulus appeared to belong to the surrounding environment. The qualitative data suggest that immersion and emotional engagement arose not only from the technology type, but also from the degree of fit between the virtual stimulus and the environment. When virtual objects matched the implied context of their setting, presence was strong. When they did not, even technically capable platforms failed to produce such strong emotional responses.

This was observed in the contamination scenario. When dirty bins and rubbish were placed over a clean room in AR, participants reported a sense of cognitive and emotional rejection. P2 noted that they subconsciously felt that *“dirty things like that shouldn’t exist in a classroom.”* P12 described the experimental environment as making the rubbish feel *“wrong*,*”* noting that *“my subconscious rejected it because it did not belong in that setting.”* P13 observed that *“if virtual content doesn’t match the physical space*,* like placing trash in a classroom*,* it just feels fake.”* P22 drew a clear distinction between AR’s higher realism (the real environment) and VR’s higher immersion (a contextually appropriate scene), concluding that for contamination exposure, the VR environment was simply more believable.

VR, by contrast, performed well in this scenario by constructing a complete and thematically consistent dirty environment. This coherence appeared sufficient to trigger associated psychological responses even when other sensory channels, such as smell and touch, were not directly stimulated. P9 reported that *“the visuals in the VR trash task were so realistic that I could almost smell the garbage. While the smell was only in my mind*,* it heightened my discomfort.”* P30 described a similar response: *“when the contaminated liquid was under my feet*,* it felt incredibly real*,* as if I were stepping on something sticky. I became much more conscious of where I was stepping.”* These accounts suggest that a sufficiently coherent visual environment may prompt the brain to generate sensory expectations beyond what is physically present, a process that may amplify emotional responses and heighten attentiveness to the surrounding space. This kind of cross-modal perceptual filling was reported more frequently in VR, where the fully enclosed environment reduces competing sensory input from the real world.

A further observation was that VR environments could activate personal memories in ways that amplified emotional responses beyond the virtual content alone. P8 noted that the VR rubbish scene brought to mind *“the corner of the narrow space beneath my accommodation*,* where there was a huge*,* dirty*,* and cluttered rubbish bin.”* P24 similarly described associating the VR environment with past experiences, noting that *“VR felt closer to real life. I could associate it with past experiences*,* like being at home or next to a real trash bin. Visually*,* it triggered memories and imagination.”* These accounts suggest that when a virtual environment is sufficiently detailed and spatially coherent, it may connect with participants’ existing emotional associations.

The opposite pattern was observed for spider stimuli. AR’s real-world backdrop, including desks, tables, and the participant’s own hands, provided precisely the kind of ordinary, familiar context in which spiders plausibly appear, which in turn amplified perceived threat. P3 noted that *“AR blurred the boundary between reality and virtual. There was always this subtle feeling that the spider might actually be real.”* P25 observed that *“seeing the spider respawn on the table and fall off made it feel very realistic and immersive*,*”* as though the spider was genuinely interacting with the physical space rather than being overlaid on it.

#### Embodiment and bodily presence

This theme concerns how the relationship between a participant’s body and the virtual stimulus modulated emotional intensity. AR and VR differed in the degree to which participants felt the virtual stimulus was acting upon their actual body.

In AR, participants could see their own hands, arms, and, when looking down, their feet and clothing in the same space as the virtual stimulus. This grounded self-image produced a strong sense of personal threat for some participants. P23 articulated the mechanism clearly: *“In AR*,* I can see my own hands*,* which makes it easier to believe that I am actually holding a spider.”* P31 reported that picking up the spider in AR and watching it move against real furniture gave them a stronger sense of presence than VR. P4 identified this as a difference in bodily agency: *“when a spider crawls on me in AR*,* it feels like it’s crawling on my actual body. In VR*,* it’s just a virtual hand*,* and the effect isn’t as strong.”*

P14 extended this to interaction with the physical environment: *“In AR*,* I could touch the table*,* but in VR*,* when I tried to touch a chair*,* there wasn’t actually a chair there. I feel like if the chair had been real*,* it would have felt more convincing. Otherwise*,* you know it’s not real.”* In VR, cross-modal incongruence, that is, seeing a chair, reaching for it, and touching nothing, undermines immersion more than the absence of furniture would, because it actively contradicts the environmental premise. In AR, the use of real physical surfaces as interaction substrates means that when a participant touches a table and the spider is on that table, the tactile reality of the surface reinforces the plausibility of the stimulus resting on it. This effect extended beyond surface contact. P3 reported that when picking up the spider, its antennae seemed to tickle their fingers despite no haptic feedback being present, and P14 described *“feeling something in their hand”* during AR interaction. These phantom tactile sensations suggest that visual and proprioceptive congruence in AR may be sufficient to generate basic tactile expectations even in the absence of physical contact.

A further dimension of AR embodiment concerns the perceptual properties of AR rendering, specifically how real-world lighting and optical effects apply to virtual objects during movement. P4 observed that AR re-renders virtual models under an approximation of real-world lighting conditions, which causes a moving spider to appear with a degree of naturalistic blur: *“it seems like things*,* like a virtual spider*,* can get a bit blurry*,* which actually makes them look more real.”* P20 described the same effect as reducing the spider’s artificial quality during movement, making it *“seem more threatening.”* This finding is counterintuitive from a rendering fidelity standpoint, but it is psychologically coherent. In everyday life, small creatures such as spiders are rarely observed with sustained, focused attention. They are more often noticed through peripheral vision or hurried glances. AR’s motion blur may mirror this perceptual quality, presenting the spider as it might appear in an authentic encounter: in motion, imperfectly seen, and less easily dismissed as a static model. VR, by rendering the spider at consistent resolution against a uniformly lit virtual background, removes this ecologically grounded threat cue.

#### Missing sensory cues

Although the previous two themes described conditions under which environmental coherence and strong bodily presence could produce incidental olfactory and tactile sensations, the absence of non-visual sensory channels remained a consistently reported limitation on the intensity and emotional effectiveness of the experience. Participants’ feedback centred on three sensory modalities.

**Haptic feedback** was the most frequently cited absence. The emotional impact of spider interaction was reduced by the inability to feel the spider on the skin. P11 made the comparison with everyday experience explicit: in real life, a crawling insect is detected through touch first, and without that signal the virtual spider registers as a visual effect rather than a bodily threat. P12 suggested controller vibration as a practical minimum intervention, noting that *“if the controller vibrated*,* like in a gamepad*,* that would feel more convincing.”* P28 described the absence of tactile crawling sensation as a gap in the experience.

**Olfactory cues** were identified as important for contamination perception. P12 stated that *“without smell*,* it just felt like another object.”* P18 observed that the banana peel *“looked too perfect - it should be rotting.”* P23 noted that a genuinely dirty environment involves *“a funky smell*,* a tactile feeling that makes you not want to touch things*,*”* both of which were absent from the virtual scenario. P28 identified smell as their primary trigger for contamination in real life, meaning that without it the virtual scenario did not reach their fear threshold.

**Spatial audio** was widely identified as an achievable improvement, specifically for contamination simulation. P4 provided the most specific design suggestions, noting that a plastic bottle hitting a bin should produce a clunk, and that a plastic bag should crinkle when handled. P5 called for sounds when closing the bin lid and rustling sounds during pick-up interactions. P1 suggested that sound effects more broadly could improve the sense of realism in the contamination scenario. P5 and P16 noted that the absence of footstep sounds or ambient environmental audio, such as flies buzzing, reduced the overall atmosphere of dirtiness.

#### Perceived control and threat unpredictability

A recurring pattern across both scenarios concerned the degree to which participants felt they had agency over their encounter with the stimulus, both in terms of whether they could avoid it spatially and whether they could anticipate when or how it would appear. These two dimensions, spatial avoidance and temporal predictability, shaped threat perception in ways that were largely independent of visual fidelity or environmental coherence.

In terms of spatial avoidance, participants in both platforms could choose not to interact directly with individual contaminated objects, and several noted that this option reduced the intensity of their emotional response. However, the VR environment itself could not be avoided in the same way. Participants remained enclosed within the dirty space regardless of whether they engaged with specific objects, meaning the environmental stimulus was continuously present. P14 described the VR contamination scene as a closed space with no room to move, and P9 reported feeling *“an urgent need to exit the environment.”* P16 described the atmosphere as *“heavy*,* like I was sealed inside a dirty place*,*”* and P17 noted feeling *“overwhelmed*,* like I couldn’t clean everything. It felt like being trapped in a place I couldn’t fix.”* P6 observed that even without physically touching anything in the environment, the surrounding atmosphere alone produced a sense of personal contamination: *“I even felt like I was dirty myself. Even though I wasn’t physically dirtied*,* and I didn’t touch other parts of the environment*,* it still felt like I was surrounded by filth.”* P11 and P21 described feeling compelled to confront the contamination context simply by being within it. AR, by contrast, retained the participant’s real-world surroundings, so unpleasant virtual elements occupied only a portion of their visual field and could be partially disregarded.

The temporal dimension of control was equally significant. Participants reported that uncertainty about when and how the stimulus would act was itself a source of threat, independent of whether the stimulus actually moved or approached them. P3 described remaining in a state of unease even when the spider was static: *“The spider was completely static… I still sensed a certain level of threat from it*,* as if it could start moving at any moment.”* Expecting unpredictable actions keeps people in a constant state of alertness. P14 noted that not knowing where the spiders had come from contributed to perceived threat, observing that *“I didn’t know where they came from*,* and that uncertainty added to the realism.”* P27 extended this further, drawing on horror game conventions: *“it’s the unknown that makes things scary - like darkness*,* or subtle signs that something bad is coming.”*

#### Combining AR and VR

In discussions of technology application preferences, AR and VR were seen less as competing alternatives and more as complementary tools that can be used at different stages of a graded exposure process. This perspective emerged consistently across participants with varying levels of phobia severity and technological familiarity.

Multiple participants endorsed AR as a more accessible starting point, primarily because its partial reality produces a less overwhelming encounter with the fear stimulus while retaining the grounding effect of the real environment. P19 stated that *“it’s better to start with AR. Jumping straight into VR might be too intense.”* P21 elaborated on their reasoning, noting that beginning with a single small spider on a real table produces a manageable level of emotional response, whereas encountering the same stimulus immediately in a fully immersive VR room surrounded by multiple spiders risks producing an intensity of reaction that is too high to engage with meaningfully. P26 described the AR to VR pathway as appropriate to their own situation: *“start with AR*,* then once I get used to it*,* move on to VR for further treatment.”* P29 similarly noted that AR *“helps participants build up confidence in a familiar setting”* before progressing to a more emotionally demanding experience. P24 described the transition as personally significant, noting that prior AR exposure meant they *“brought the experience from AR into VR”* with reduced anxiety, suggesting that lower-intensity AR exposure functioned as preparation for the stronger emotional demands of VR.

Beyond the initial stage, participants widely endorsed a structured escalation in experience intensity from AR to VR. P21 outlined a specific sequence for virtual spider scenarios, suggesting that *“in AR*,* you can start with one small*,* static spider. Once the user is used to it*,* you can gradually introduce movement*,* and then shift to a VR environment with more immersion.”* P25 endorsed a similar approach, suggesting that AR allows participants to *“develop a better relationship with their fear”* in a familiar space, while VR offers the additional capability of constructing scenarios that produce a higher level of experience intensity, such as a greater number of spiders or a more extensively contaminated environment. P28 noted a practical distinction between the two platforms, suggesting that AR suits everyday scenarios such as a spider in a domestic room, while VR is better positioned to model more extreme or unfamiliar situations that would be difficult to reproduce in AR and that carry a correspondingly higher emotional impact.

## Discussion

The results showed a significant main effect of technology type and a significant interaction between technology type and stimulus type, indicating that the relative effectiveness of AR and VR in eliciting experience intensity varies across scenarios. Table 3 summarises the findings in relation to each research question. The following sections interpret these findings and consider their implications for the design and use of immersive systems for exposure-based therapy.

**Table 3:** Summary of Findings

Research Question	Outcome	Key finding
RQ1: Technology effect	VR stronger overall	VR produced higher experience intensity overall. (However see RQ2.)
RQ2: Technology × scenario interaction	Depends on scenario	VR’s advantage was seen in the contamination condition. AR performed comparably to VR for spider exposure and offered advantages in this scenario.

### RQ1: overall technology comparison

VR produced higher experience intensity than AR when averaged across both scenarios. However, this overall difference should not be read as a simple ranking of the two technologies, because of the significant interaction effect (discussed in Sect.  5.2), with VR’s advantage being mainly in the contamination condition, with no significant difference observed between the two technologies in the spider scenario. The following section examines each scenario in detail to explore the mechanisms behind this interaction.

### RQ2: technology–scenario interaction

When averaged across both technologies, the spider and contamination scenarios produced very similar levels of experience intensity. We would expect the experience intensity to depend on both the effectiveness of the stimulus (in vivo and virtual) and on the prevalence and severity of the corresponding phobia in the study population. Although participants self-reported similar levels of discomfort with spiders and contaminants (M = 4.16 vs. 4.71 on a 7-point scale) we have no objective assessment of this. We also have no baseline assessment of participants’ responses to corresponding in vivo stimuli. As such we cannot be sure whether the spider and contamination scenarios are equally effective in eliciting a response, or to what extent differences in response may be due to the choice of in-vivo stimulus (e.g. a small live/moving spider) versus the quality of the virtual model.

However, the within-subjects study design and the consistency of the AR and VR versions of each scenario mean that the observed differences between AR and VR versions of the same scenario still reflect significant differences in how each technology performed for these scenarios and participants. The following subsections discuss each scenario in turn.

#### Spider scenario: ecological congruence, bodily presence, and threat unpredictability

The absence of a significant intensity difference between AR and VR in the spider scenario suggests that the two technologies may achieve comparable levels of affective engagement albeit through different mechanisms. Rather than relying on full sensory immersion, AR may derive some of its effectiveness in this context from situating the virtual stimulus within a familiar physical environment, with the real-world background contributing to threat perception. Spiders are plausibly encountered on household surfaces, tables, and the body itself, which are real-world elements retained in the AR condition. Several participants described AR as blurring the boundary between the virtual and the real in a way that VR did not, and the presence of their own visible hands and arms in the scene appeared to strengthen the sense that the spider was acting on their actual body. The role of body visibility in AR has been noted in prior exposure research. Juan and Pérez (2010) found that participants considered it important to be able to see their own feet and body during AR exposure, and identified this as a distinguishing advantage of AR over VR. Jurcik et al. (2024) similarly observed that the appearance of virtual spiders within participants’ real surroundings, including in proximity to their own hands and body, was associated with effective anxiety reduction in an ET-AR intervention.

One finding concerns the effect of AR’s real-world optical rendering on moving spider stimuli. Participants reported that spiders appeared with natural motion blur under real-world lighting, and described this as making the spider appear more threatening. From a rendering fidelity perspective, this is counterintuitive, as blur is usually associated with lower image quality. Psychologically, however, it is coherent. This is consistent with Bretón-López et al. (2010), who found that moving virtual animals elicit greater anxiety than static ones. In everyday life, small creatures such as spiders are rarely observed with sustained attention. They are more often noticed through peripheral vision or brief glances, or in states of heightened arousal where attentional focus reduces peripheral clarity. Thus, in AR the spider is presented as it might appear in an authentic encounter (in motion, imperfectly seen) rather than as a static, high-resolution model available for sustained inspection. VR, by rendering the spider more precisely against a uniformly lit virtual background, may undermine these ecological threat cues. This suggests that rendering accuracy and emotional authenticity are not necessarily aligned, and that stimuli which appear technically inferior may, in some contexts, produce stronger psychological responses.

Beyond visual properties, the qualitative data also point to threat unpredictability as an additional factor shaping threat intensity. Participants reported that uncertainty about when and how the spider might move was a source of unease independent of visual quality. Stimuli that were temporally unpredictable, appearing able to move at any moment or arrive from an unknown location, encouraged a state of vigilance that static or predictable stimuli did not. This suggests that control of dynamics function as a complementary variable in ET-V alongside stimulus realism. The design of exposure scenarios should attend not only to how a stimulus looks, but also to the degree of predictability and the spatial congruence experienced by the participant during the encounter.

Furthermore, the correlation results offer an additional perspective on AR’s ecological validity in this scenario. A significant positive correlation was found between participants’ baseline spider discomfort and their experience intensity in the AR condition, whereas no such relationship emerged in the VR condition. This suggests that AR may align well with participants’ pre-existing, individualised fear sensitivities. As the AR spider appears within the user’s direct view of their personal space, the intensity of the experience seems to scale with their underlying baseline sensitivity. In contrast, the visually immersive nature of VR may produce a more generalized level of arousal that could obscure individual differences in aversive sensitivity.

#### Contamination scenario: stimulus-context congruence, spatial avoidance, and cross-modal perceptual filling

In the contamination scenario, VR produced significantly higher experience intensity than AR. The qualitative data indicate that this difference was primarily due to a mismatch between AR’s virtual contamination stimuli and the real-world environment in which they were presented. Placing dirty rubbish in a clean classroom produced a sense of incongruence that participants described as cognitively and emotionally unconvincing. The virtual objects felt implausible not because of their visual quality, but because they did not belong in the surrounding space. By contrast, VR’s fully virtual streetscape provided a visually coherent dirty environment in which the contamination stimuli were not isolated overlays but part of the whole scene.

A further mechanism contributing to VR’s stronger performance in the contamination scenario concerns spatial avoidance. In the AR condition, unpleasant virtual elements occupied only part of the visual field and could be partially disregarded by looking away or redirecting attention to the unaffected real-world surroundings. In VR, this option was not available. Participants remained enclosed within the dirty environment regardless of whether they chose to interact with any specific object, meaning the contamination stimulus was continuously present throughout the exposure. As reported in the Perceived Control theme, several participants described the experience in terms of inescapability. This suggests that spatial inescapability may function as a contributor to experience intensity in VR. Exposure to the contamination stimulus was not contingent on active engagement with individual objects, but was sustained by the environment itself. This is consistent with the broader principle that reducing a participant’s capacity for spatial avoidance increases the extent to which the fear structure is engaged during exposure (Foa and Kozak 1986).

This stimulus-context congruence had further perceptual consequences. Several participants in the VR condition reported sensory impressions that the prototype did not physically provide. The rubbish appeared to produce a smell, and contaminated surfaces felt sticky underfoot. These responses are consistent with the neurobiological model proposed by Bouchard et al. (2008), in which a sufficiently coherent virtual environment triggers instinctive emotional reactions that are subsequently integrated into conscious aversive responses and a heightened sense of presence. Under this account, cross-modal perceptual filling may arise from the brain generating sensory expectations consistent with the emotional context it has accepted as real. VR’s fully enclosed environment reduces competing real-world sensory input, making this integration more likely. AR retains real-world perceptual channels that may partially suppress such percepts, specifically when the surrounding real environment is contextually incompatible with the virtual stimuli. A related finding was that the VR environment also appeared to activate personal memories. Several participants associated the virtual dirty streetscape with specific past experiences, reporting that these associations amplified their emotional response. This suggests that a spatially coherent virtual environment may activate existing emotional associations, which may partly account for VR’s stronger performance in this scenario.

### A staged exposure approach?

The pattern of results across both scenarios, together with the technology preference data, suggests an exposure-based therapy model in which AR and VR are not competing alternatives but complementary choices or even stages in a graded exposure approach. Participants broadly framed the two technologies in these terms. While a majority preferred AR for the spider scenario and VR for the contamination scenario, many described a progression from AR to VR as potentially therapeutically appropriate. AR was seen as a more accessible starting point, providing initial contact with the fear stimulus in familiar, real-world surroundings at a manageable intensity. VR was considered suitable for later-stage exposure, where the goal is to produce greater environmental immersion and affective engagement.

At least for immersive or atmospheric stimuli such as contamination, participants felt that AR’s lower initial exposure intensity may reduce the risk of overwhelming anxiety and encourage compliance. VR’s capacity for full environmental control and context coherence may make it more appropriate for later-stage exposure where maximising affective engagement is the primary goal. Together, these findings suggest that technology choice in exposure-based therapy should not be treated as a fixed design decision but as a treatment variable to be adjusted according to stimulus type, and perhaps also exposure stage, and individual patient characteristics or preferences.

### Limitations

Several limitations should be noted when interpreting these findings. First, the scope of the present study should be noted. The study recruited a healthy non-clinical population and did not assess clinical outcomes. As discussed in Sect.  2.3, the mechanisms linking presence and emotional activation in virtual environments are not exclusive to clinical populations, and self-reported measures of experience intensity have been established as reliable indicators of affective engagement. This approach is therefore consistent with prior foundational work that has similarly used general population samples to establish the affective properties of virtual environments before clinical deployment. However, these measures do not assess physiological response, clinical treatment outcomes or long-term symptom reduction. The practical recommendations outlined in this study, including the possible AR-then-VR approach, should therefore be understood as preliminary insights informed by experiential data rather than clinical evidence. Whether the observed differences in experience intensity between AR and VR translate into differential therapeutic efficacy in clinical populations remains an open question.

Second, the study adopted a short-term, single-session exposure design, which does not reflect the gradual and repeated nature of standard exposure-based therapy. It also remains possible that initial evaluations were influenced by novelty effects associated with immersive technology. However, the within-subjects design, in which all participants experienced both AR and VR conditions using the same hardware across all scenarios, means that any novelty-related arousal should have applied equally to both technologies. The use of a single device (Meta Quest 3) for all conditions further ensures that hardware-related novelty was held constant, mitigating the risk that differences in experience intensity between AR and VR were driven by differential novelty responses. This is further supported by the correlation analysis, which revealed no significant association between prior familiarity with AR or VR and reported experience intensity across any condition. This suggests that the observed emotional and experiential responses were unlikely to be driven by technological novelty or prior exposure to the devices. Nonetheless, how the observed differences in intensity and preference might evolve across extended treatment periods remains unknown.

Third, the use of a specific device with particular affordances, including hand tracking and high-resolution colour passthrough, may affect the transferability of the findings to other AR and VR systems with different hardware capabilities. Findings may not generalise to platforms that rely on controller-based input or lower-fidelity passthrough, where the bodily presence and environmental coherence effects identified in this study may manifest differently.

Fourth, the absence of a neutral control condition limits the ability to distinguish stimulus-specific aversive responses from general arousal induced by immersive technology. While efforts were made to match AR and VR conditions in visual complexity and task structure, the stimuli were not pre-validated for equivalence in subjective discomfort. Some of the observed differences in experience intensity may therefore reflect unmeasured affective differences between stimulus sets rather than purely technological effects.

Finally, with respect to sample characteristics, although the within-subjects design (*N* = 31) provided sufficient statistical power to detect main and interaction effects, the sample size remains relatively small, limiting broader generalisability. In addition, as the demographic composition is skewed towards younger adults or university students, the findings may not fully generalise to other (e.g. older) populations, who may differ in technology acceptance or susceptibility to cybersickness.

### Future work

Future experimental designs could extend to longitudinal, multi-session trials. The single-session design adopted here does not reflect the repeated nature of standard exposure-based therapy, and it remains unclear how user preferences and the relative performance of AR and VR might change over time or once novelty effects diminish. One direction worth exploring would be to empirically test the AR-then-VR approach suggested by participants in this study, by examining whether a structured transition from AR to VR across sessions produces better outcomes or improved adherence compared to using either technology alone. Future studies might also consider incorporating objective physiological measures such as heart rate variability and skin conductance to complement self-reported data and provide a more comprehensive assessment of emotional responses across sessions. Expanding and diversifying participant samples to include a wider range of ages and educational backgrounds would also help improve the generalisability of the findings.

This study examined two stimulus conditions. Future work could extend this comparison to a wider range of exposure scenarios with different focal, dynamic, and contextual properties, to explore whether the patterns observed here generalise across other stimulus types. This would contribute to more informed guidelines for matching technology to stimulus type for use in exposure-based therapy. Similarly, including a baseline assessment of participants’ responses to equivalent non-virtual stimuli could help to tease apart the effects of stimulus choice from technology choice.

Building on the affective engagement data established in this study, future research could move towards assessing therapeutic outcomes more directly. This would involve incorporating pre- and post-intervention measures of symptom severity, comparing outcomes against traditional in vivo exposure-based therapy as a baseline, and recruiting clinical populations with relevant conditions. Such a progression would allow the present findings to be situated within a broader clinical validation framework, and would help determine whether the technology-stimulus-response patterns identified here hold when treatment efficacy, rather than experience intensity, is the primary outcome.

Further development of the prototype is another direction worth considering. Participants’ qualitative feedback (Sect.  4.4.3) indicated that the absence of haptic, olfactory, and spatial audio feedback was a consistently reported limitation on the intensity and realism of the experience. Future iterations could therefore explore the integration of multimodal sensory channels, such as vibrotactile feedback for spider interaction, spatial audio for contamination scenarios, and olfactory cues where feasible. Improvements to interface design, user guidance, and system stability would also be worth addressing in future versions. If the system were to be deployed in a clinical context, a multi-user version could allow the patient and therapist to share the exposure environment simultaneously, providing the therapist with greater insight into the patient’s experience and potentially enabling remote delivery of therapy.

## Conclusion

This study compared the experience intensity produced by VR and AR across two exposure scenarios using virtual stimuli (spider and contamination), in a within-subjects design with 31 participants. The results showed that VR produced significantly higher experience intensity than AR overall, and that this difference was not uniform across stimulus types: VR was significantly more effective in the contamination scenario, while the two technologies performed comparably for spider stimuli.

The qualitative data suggest that these differences were shaped by several factors, including the degree of fit between the virtual stimulus and its surrounding environment, the relationship between the participant’s body and the virtual stimulus, and the degree of spatial control afforded to the participant during the exposure. For contamination stimuli, VR’s fully enclosed environment provided a contextually coherent setting that AR, incorporating real-world surroundings, could not replicate. For spider stimuli, AR’s real-world backdrop and the visibility of the participant’s own body contributed to a level of ecological validity that appeared to compensate for its reduced sensory immersion.

These findings suggest that AR and VR are not interchangeable in exposure-based therapy applications, and that technology selection may need to be guided by the specific characteristics of the stimulus being modelled. The results also point towards a possible graduated approach for some phobias, in which AR might be used in earlier stages of exposure to provide a more accessible initial encounter with the stimulus, with a transition to VR as the exposure progresses. This study offers a basis for future investigation with clinical populations, as well as insights that might help other experience designers to make a better-informed choice between AR and VR.

## Supplementary Information

Below is the link to the electronic supplementary material.

Supplementary Material 1

Supplementary Material 2

Supplementary Material 3

Supplementary Material 4

Supplementary Material 5

## Data Availability

All data is provided within the manuscript or supplementary information files.

## Appendix A: Experience intensity questionnaire

### Initial Questions.

**Table:**

No.	Question Content	Rating/Choice Range
1	Participant ID	–
2	Age	–
3	Gender	Female, Male, Non-binary, Prefer not to say, Other
4	Familiarity with Augmented Reality (AR) technology	Familiar, Limited experience (used less than 5 times), Never used
5	Familiarity with Virtual Reality (VR) technology	Familiar, Limited experience (used less than 5 times), Never used
6	Which devices have you used for AR or VR?	Meta Quest, Microsoft HoloLens, Google Glass, etc.
7	Are you familiar with VR or AR exposure therapy?	Yes, I have heard of it Yes, I have experienced it No, I don’t know what it is, Other
8	Comfort level when encountering spiders	Scale from 1 (Completely comfortable) to 7 (Very scared)
9	Concern about contaminants (dirty things) in your environment	Scale from 1 (Not at all concerned) to 7 (Very concerned)

### Experience Intensity Questionnaire - Part 1: Virtual Spider.

**Table:**

No.	Question Content	AR	VR	Rating/Choice Range
10	To what extent did it feel like real spiders were actually present around you?			Scale from 1 (Not at all) to 7 (Extremely)
11	How realistic did the static spiders appear?			
12	How realistic did the spiders appear when they moved?			
13	To what extent did the interaction with the virtual spiders feel realistic?			
14	To what extent did you feel resistance or avoidance when asked to interact with virtual spiders?			
15	To what extent did you feel a sensation when the spiders made contact with your hand?			
16	To what extent did you have an emotional reaction to the virtual spiders?			

**Table:**

No.	Question Content	Rating/Choice Range
17	How did you feel about the realism of the virtual spider in VR compared to AR?	VR less/more realistic than AR, No difference, Other
18	Do you prefer VR or AR technology for exposure therapy for spider phobia?	VR, AR, No preference, Neither, Other

### Experience Intensity Questionnaire - Part 2: Contamination Simulation.

**Table:**

No.	Question Content	AR	VR	Rating/Choice Range
19	To what extent did you feel like you were in a dirty environment?			Scale from 1 (Not at all) to 7 (Extremely)
20	To what extent did you feel the dirtiness when observing rubbish on the ground?			
21	To what extent did you feel the dirtiness when grabbing and touching a banana peel?			
22	To what extent did you feel resistance or avoidance when asked to interact with dirty items?			
23	To what extent did you feel relieved when throwing away dirty things?			
24	To what extent did you feel uncomfortable when reaching into the bin to grab dirty items?			
25	To what extent did you feel like your hands are actually getting dirty when touching dirty items?			

**Table:**

No.	Question Content	Rating/Choice Range
26	How did you feel about the realism of the dirty things simulation in VR compared to AR?	VR less/more realistic than AR, No difference, Other
27	Do you prefer VR or AR technology for exposure therapy for contamination fears?	VR, AR, No preference, Neither, Other

## Appendix B: Semi-structured interview

**Table:**

No.	Question Content
1	Could you describe your main feelings while completing the tasks in VR compared to AR? How did the realism and immersion of the exposure scenarios in VR compare to AR?
2	Did you encounter any difficulties while interacting with the VR or AR environments? If so, what were they?
3	Did you feel a strong emotional response when performing any particular task? If so, which task and what was your response? Was it the same between two technologies?
4	Could you compare your feelings towards the two scenarios (spiders and contamination)? What are the main differences between them?
5	Do you think AR and VR are suitable tools for exposure therapy? Do they have any pros and cons?
6	In the preference question in the questionnaire, you chose AR/VR. What are your reasons? Is one better than the other?
7	Do you have any comments or suggestions (issues/improvements) for the prototype and the exposure experience process? What elements do you think would make it feel more realistic/immersive/effective?
8	Any additional feedback or anything else you would like to share about your experience?

## Footnotes

1
https://unity3d.com/.
