# Supplementary material for: Comparing experience intensity of AR and VR for contrasting phobia stimuli
Source: Virtual Real. 2026 Aug 11;30(4):205. doi: 10.1007/s10055-026-01450-y (PMC13748273; doi:10.1007/s10055-026-01450-y)
Supplement: Supplementary file 1 [file 10055_2026_1450_MOESM4_ESM.pdf]

General Linear Model

Notes

Contents		
Output Created		28-AUG-2024 02:09:07
Comments		
Input	Active Dataset	DataSet4
	Filter	<none>
	Weight	<none>
	Split File	<none>
	N of Rows in Working Data File	31
Missing Value Handling	Definition of Missing	User-defined missing values are treated as missing.
	Cases Used	Statistics are based on all cases with valid data for all variables in the model.
Syntax		GLM Spider_AR Spider_VR Contamination_AR Contamination_VR<br>/WSFACTOR=Situation 4 Polynomial<br>/METHOD=SSTYPE(3)<br>/CRITERIA=ALPHA(.05)<br>/WSDESIGN=Situation.
Resources	Processor Time	0 00:00:00.03
	Elapsed Time	0 00:00:00.00

[DataSet4]

Within-Subjects Factors

MEASURE_1

Situation	Dependent Variable
1	Spider_AR
2	Spider_VR
3	Contamination_AR
4	Contamination_VR

Multivariate Tests[a]

Effect		Value	F	Hypothesis df	Error df	Sig.
Situation	Pillai's Trace	.452	7.703[b]	3.000	28.000	.001
	Wilks' Lambda	.548	7.703[b]	3.000	28.000	.001
	Hotelling's Trace	.825	7.703[b]	3.000	28.000	.001
	Roy's Largest Root	.825	7.703[b]	3.000	28.000	.001

a. Design: Intercept
Within Subjects Design: Situation[a]

b. Exact statistic

Mauchly's Test of Sphericity[a]

MEASURE_1

					Epsilon[b]		
Within Subjects Effect	Mauchly's W	Approx. Chi-Square	df	Sig.	Greenhouse-Geisser	Huynh-Feldt	Lower-bound
Situation	.538	17.802	5	.003	.694	.746	.333

Tests the null hypothesis that the error covariance matrix of the orthonormalized transformed dependent variables is proportional to an identity matrix.

a. Design: Intercept
Within Subjects Design: Situation[a]

b. May be used to adjust the degrees of freedom for the averaged tests of significance. Corrected tests are displayed in the Tests of Within-Subjects Effects table.

Tests of Within-Subjects Effects

MEASURE_1

Source		Type III Sum of Squares	df	Mean Square	F	Sig.
Situation	Sphericity Assumed	785.895	3	261.965	4.581	.005
	Greenhouse-Geisser	785.895	2.081	377.684	4.581	.013
	Huynh-Feldt	785.895	2.239	351.033	4.581	.011
	Lower-bound	785.895	1.000	785.895	4.581	.041
Error(Situation)	Sphericity Assumed	5146.355	90	57.182		
	Greenhouse-Geisser	5146.355	62.425	82.441		
	Huynh-Feldt	5146.355	67.164	76.624		
	Lower-bound	5146.355	30.000	171.545		

Tests of Within-Subjects Contrasts

MEASURE_1

Source	Situation	Type III Sum of Squares	df	Mean Square	F	Sig.
Situation	Linear	51.679	1	51.679	.521	.476
	Quadratic	276.008	1	276.008	10.251	.003
	Cubic	458.208	1	458.208	10.104	.003
Error(Situation)	Linear	2978.071	30	99.269		
	Quadratic	807.742	30	26.925		
	Cubic	1360.542	30	45.351		

Tests of Between-Subjects Effects

MEASURE_1
Average

Source	Type III Sum of Squares	df	Mean Square	F	Sig.
Intercept	110940.976	1	110940.976	407.783	.000
Error	8161.774	30	272.059		

Explore

Notes

Contents		
Output Created		28-AUG-2024 12:53:50
Comments		
Input	Active Dataset	DataSet4
	Filter	<none>
	Weight	<none>
	Split File	<none>
	N of Rows in Working Data File	31
Missing Value Handling	Definition of Missing	User-defined missing values for dependent variables are treated as missing.
	Cases Used	Statistics are based on cases with no missing values for any dependent variable or factor used.

Contents		
Syntax		EXAMINE VARIABLES=Spider_AR Spider_VR Contamination_AR Contamination_VR<br>/PLOT BOXPLOT STEMLEAF NPPLOT<br>/COMPARE GROUPS<br>/STATISTICS DESCRIPTIVES<br>/CINTERVAL 95<br>/MISSING LISTWISE<br>/NOTOTAL.
Resources	Processor Time	0 00:00:02.58
	Elapsed Time	0 00:00:02.00

Case Processing Summary

	Cases					
	Valid		Missing		Total	
	N	Percent	N	Percent	N	Percent
Spider_AR	31	100.0%	0	.0%	31	100.0%
Spider_VR	31	100.0%	0	.0%	31	100.0%
Contamination_AR	31	100.0%	0	.0%	31	100.0%
Contamination_VR	31	100.0%	0	.0%	31	100.0%

Descriptives

			Statistic	Std. Error
Spider_AR	Mean		29.68	1.753
	95% Confidence Interval for Mean	Lower Bound	26.10	
		Upper Bound	33.26	
	5% Trimmed Mean		29.59	
	Median		30.00	
	Variance		95.226	
	Std. Deviation		9.758	
	Minimum		14	
	Maximum		48	
	Range		34	
	Interquartile Range		16	
	Skewness		-.011	.421
	Kurtosis		-.919	.821
Spider_VR	Mean		30.71	1.822
	95% Confidence Interval for Mean	Lower Bound	26.99	
		Upper Bound	34.43	
	5% Trimmed Mean		30.60	
	Median		32.00	
	Variance		102.880	
	Std. Deviation		10.143	
	Minimum		14	
	Maximum		49	
	Range		35	
	Interquartile Range		18	
	Skewness		.034	.421
	Kurtosis		-1.269	.821
Contamination_AR	Mean		26.13	1.965
	95% Confidence Interval for Mean	Lower Bound	22.12	
		Upper Bound	30.14	

			Statistic	Std. Error
	5% Trimmed Mean		26.25	
	Median		27.00	
	Variance		119.716	
	Std. Deviation		10.941	
	Minimum		8	
	Maximum		42	
	Range		34	
	Interquartile Range		20	
	Skewness		-.044	.421
	Kurtosis		-1.343	.821
Contamination_VR	Mean		33.13	2.014
	95% Confidence Interval for Mean	Lower Bound	29.02	
		Upper Bound	37.24	
	5% Trimmed Mean		33.55	
	Median		35.00	
	Variance		125.783	
	Std. Deviation		11.215	
	Minimum		9	
	Maximum		49	
	Range		40	
	Interquartile Range		19	
	Skewness		-.391	.421
	Kurtosis		-.919	.821

Tests of Normality

	Kolmogorov-Smirnov[a]			Shapiro-Wilk		
	Statistic	df	Sig.	Statistic	df	Sig.
Spider_AR	.078	31	.200*	.968	31	.459
Spider_VR	.131	31	.186	.945	31	.116
Contamination_AR	.151	31	.068	.927	31	.037
Contamination_VR	.149	31	.076	.943	31	.100

a. Lilliefors Significance Correction

*. This is a lower bound of the true significance.

Spider_AR

```
Spider_AR Stem-and-Leaf Plot

Frequency Stem & Leaf

3.00 1 . 444
3.00 1 . 689
5.00 2 . 02344
4.00 2 . 7889
6.00 3 . 022334
5.00 3 . 55899
3.00 4 . 124
2.00 4 . 58

Stem width: 10
Each leaf: 1 case(s)
```

graphs not yet implemented

graphs not yet implemented

graphs not yet implemented

Spider_VR

```
Spider_VR Stem-and-Leaf Plot

Frequency Stem & Leaf

1.00 1 . 4
5.00 1 . 77888
5.00 2 . 22333
4.00 2 . 5778
3.00 3 . 234
5.00 3 . 56889
6.00 4 . 001224
2.00 4 . 79

Stem width: 10
Each leaf: 1 case(s)
```

graphs not yet implemented

graphs not yet implemented

graphs not yet implemented

Contamination_AR

```
Contamination_AR Stem-and-Leaf Plot

Frequency Stem & Leaf

2.00 0 . 89
4.00 1 . 1134
5.00 1 . 68899
4.00 2 . 1133
3.00 2 . 789
4.00 3 . 0122
4.00 3 . 8899
5.00 4 . 00012

Stem width: 10
Each leaf: 1 case(s)
```

graphs not yet implemented

graphs not yet implemented

graphs not yet implemented

Contamination_VR

```
Contamination_VR Stem-and-Leaf Plot

Frequency Stem & Leaf

1.00 0 . 9
1.00 1 . 3
1.00 1 . 7
6.00 2 . 012333
4.00 2 . 5999
2.00 3 . 23
3.00 3 . 567
7.00 4 . 0011224
6.00 4 . 567779

Stem width: 10
Each leaf: 1 case(s)
```

graphs not yet implemented

graphs not yet implemented

graphs not yet implemented

General Linear Model

Notes

Contents	
Output Created	28-AUG-2024 13:04:00

Contents		
Comments		
Input	Active Dataset	DataSet4
	Filter	<none>
	Weight	<none>
	Split File	<none>
	N of Rows in Working Data File	31
Missing Value Handling	Definition of Missing	User-defined missing values are treated as missing.
	Cases Used	Statistics are based on all cases with valid data for all variables in the model.
Syntax		GLM Spider_AR Contamination_AR Spider_VR Contamination_VR<br>/WSFACTOR=Technology 2 Simple(1) Project 2 Simple(1)<br>/METHOD=SSTYPE(3)<br>/PRINT=DESCRIPTIVE ETASQ HOMOGENEITY<br>/CRITERIA=ALPHA(.05)<br>/WSDESIGN=Technology Project Technology*Project.
Resources	Processor Time	0 00:00:00.05
	Elapsed Time	0 00:00:00.00

Warnings

The HOMOGENEITY specification in the PRINT subcommand will be ignored because there are no between-subjects factors.

Within-Subjects Factors

MEASURE_1

Technology	Project	Dependent Variable
1	1	Spider_AR
	2	Contamination_AR
2	1	Spider_VR
	2	Contamination_VR

Descriptive Statistics

	Mean	Std. Deviation	N
Spider_AR	29.68	9.758	31
Contamination_AR	26.13	10.941	31
Spider_VR	30.71	10.143	31
Contamination_VR	33.13	11.215	31

Multivariate Tests[a]

Effect		Value	F	Hypothesis df	Error df	Sig.	Partial Eta Squared
Technology	Pillai's Trace	.324	14.358[b]	1.000	30.000	.001	.324
	Wilks' Lambda	.676	14.358[b]	1.000	30.000	.001	.324
	Hotelling's Trace	.479	14.358[b]	1.000	30.000	.001	.324
	Roy's Largest Root	.479	14.358[b]	1.000	30.000	.001	.324
Project	Pillai's Trace	.003	.090[b]	1.000	30.000	.766	.003
	Wilks' Lambda	.997	.090[b]	1.000	30.000	.766	.003
	Hotelling's Trace	.003	.090[b]	1.000	30.000	.766	.003
	Roy's Largest Root	.003	.090[b]	1.000	30.000	.766	.003
Technology *	Pillai's Trace	.255	10.251[b]	1.000	30.000	.003	.255

Effect		Value	F	Hypothesis df	Error df	Sig.	Partial Eta Squared
Project	Wilks' Lambda	.745	10.251[b]	1.000	30.000	.003	.255
	Hotelling's Trace	.342	10.251[b]	1.000	30.000	.003	.255
	Roy's Largest Root	.342	10.251[b]	1.000	30.000	.003	.255

a. Design: Intercept
Within Subjects Design: Technology + Project + Technology * Project[a]

b. Exact statistic

Mauchly's Test of Sphericity[a]

MEASURE_1

Within Subjects Effect	Mauchly's W	Approx. Chi-Square	df	Sig.	Epsilon[b] Greenhouse-Geisser	Huynh-Feldt	Lower-bound
Technology	1.000	.000	0	.	1.000	1.000	1.000
Project	1.000	.000	0	.	1.000	1.000	1.000
Technology * Project	1.000	.000	0	.	1.000	1.000	1.000

Tests the null hypothesis that the error covariance matrix of the orthonormalized transformed dependent variables is proportional to an identity matrix.

a. Design: Intercept
Within Subjects Design: Technology + Project + Technology * Project[a]

b. May be used to adjust the degrees of freedom for the averaged tests of significance. Corrected tests are displayed in the Tests of Within-Subjects Effects table.

Tests of Within-Subjects Effects

MEASURE_1

Source		Type III Sum of Squares	df	Mean Square	F	Sig.	Partial Eta Squared
Technology	Sphericity Assumed	500.008	1	500.008	14.358	.001	.324
	Greenhouse-Geisser	500.008	1.000	500.008	14.358	.001	.324
	Huynh-Feldt	500.008	1.000	500.008	14.358	.001	.324
	Lower-bound	500.008	1.000	500.008	14.358	.001	.324
Error(Technology)	Sphericity Assumed	1044.742	30	34.825			
	Greenhouse-Geisser	1044.742	30.000	34.825			
	Huynh-Feldt	1044.742	30.000	34.825			
	Lower-bound	1044.742	30.000	34.825			
Project	Sphericity Assumed	9.879	1	9.879	.090	.766	.003
	Greenhouse-Geisser	9.879	1.000	9.879	.090	.766	.003
	Huynh-Feldt	9.879	1.000	9.879	.090	.766	.003
	Lower-bound	9.879	1.000	9.879	.090	.766	.003
Error(Project)	Sphericity Assumed	3293.871	30	109.796			
	Greenhouse-Geisser	3293.871	30.000	109.796			
	Huynh-Feldt	3293.871	30.000	109.796			
	Lower-bound	3293.871	30.000	109.796			
Technology * Project	Sphericity Assumed	276.008	1	276.008	10.251	.003	.255

Source		Type III Sum of Squares	df	Mean Square	F	Sig.	Partial Eta Squared
	Greenhouse-Geisser	276.008	1.000	276.008	10.251	.003	.255
	Huynh-Feldt	276.008	1.000	276.008	10.251	.003	.255
	Lower-bound	276.008	1.000	276.008	10.251	.003	.255
Error(Technology*Project)	Sphericity Assumed	807.742	30	26.925			
	Greenhouse-Geisser	807.742	30.000	26.925			
	Huynh-Feldt	807.742	30.000	26.925			
	Lower-bound	807.742	30.000	26.925			

Tests of Within-Subjects Contrasts

MEASURE_1

Source	Technology	Project	Type III Sum of Squares	df	Mean Square	F	Sig.	Partial Eta Squared
Technology	Level 2 vs. Level 1		500.008	1	500.008	14.358	.001	.324
Error(Technology)	Level 2 vs. Level 1		1044.742	30	34.825			
Project		Level 2 vs. Level 1	9.879	1	9.879	.090	.766	.003
Error(Project)		Level 2 vs. Level 1	3293.871	30	109.796			
Technology * Project	Level 2 vs. Level 1	Level 2 vs. Level 1	1104.032	1	1104.032	10.251	.003	.255
Error(Technology*Project)	Level 2 vs. Level 1	Level 2 vs. Level 1	3230.968	30	107.699			

Tests of Between-Subjects Effects

MEASURE_1
Average

Source	Type III Sum of Squares	df	Mean Square	F	Sig.	Partial Eta Squared
Intercept	27735.244	1	27735.244	407.783	.000	.931
Error	2040.444	30	68.015			

T-Test

Notes

Contents		
Output Created		04-SEP-2024 17:52:00
Comments		
Input	Data	/Users/apple/Desktop/Raw Data.sav
	Active Dataset	DataSet2
	Filter	<none>
	Weight	<none>
	Split File	<none>
	N of Rows in Working Data File	31

Contents		
Missing Value Handling	Definition of Missing	User defined missing values are treated as missing.
	Cases Used	Statistics for each analysis are based on the cases with no missing or out-of-range data for any variable in the analysis.
Syntax		T-TEST PAIRS=Spider_AR Spider_VR WITH Contamination_AR Contamination_VR (PAIRED)<br>/ES DISPLAY(FALSE)<br>/CRITERIA=CI(.9500)<br>/MISSING=ANALYSIS.
Resources	Processor Time	0 00:00:00.02
	Elapsed Time	0 00:00:01.00

Paired Samples Statistics

		Mean	N	Std. Deviation	Std. Error Mean
Pair 1	Spider_AR	29.68	31	9.758	1.753
	Contamination_AR	26.13	31	10.941	1.965
Pair 2	Spider_VR	30.71	31	10.143	1.822
	Contamination_VR	33.13	31	11.215	2.014

Paired Samples Correlations

				Significance	
		N	Correlation	One-Sided p	Two-Sided p
Pair 1	Spider_AR & Contamination_AR	31	.304	.048	.096
Pair 2	Spider_VR & Contamination_VR	31	.462	.004	.009

Paired Samples Test

		Paired Differences						
					95% Confidence Interval of the Difference			
		Mean	Std. Deviation	Std. Error Mean	Lower	Upper	t	df
Pair 1	Spider_AR - Contamination_AR	3.548	12.244	2.199	-.943	8.040	1.614	30
Pair 2	Spider_VR - Contamination_VR	-2.419	11.114	1.996	-6.496	1.657	-1.212	30

		Significance	
		One-Sided p	Two-Sided p
Pair 1	Spider_AR - Contamination_AR	.059	.117
Pair 2	Spider_VR - Contamination_VR	.117	.235

General Linear Model

Notes

Contents		
Output Created		04-SEP-2024 18:06:20
Comments		
Input	Active Dataset	DataSet2

Contents		
	Filter	<none>
	Weight	<none>
	Split File	<none>
	N of Rows in Working Data File	31
Missing Value Handling	Definition of Missing	User-defined missing values are treated as missing.
	Cases Used	Statistics are based on all cases with valid data for all variables in the model.
Syntax		GLM Spider_AR Spider_VR Contamination_AR Contamination_VR<br>/WSFACTOR=Project 2 Polynomial Technology 2 Polynomial<br>/METHOD=SSTYPE(3)<br>/CRITERIA=ALPHA(.05)<br>/WSDESIGN=Project Technology Project*Technology.
Resources	Processor Time	0 00:00:00.05
	Elapsed Time	0 00:00:00.00

[DataSet2]

Within-Subjects Factors

MEASURE_1

Project	Technology	Dependent Variable
1	1	Spider_AR
	2	Spider_VR
2	1	Contamination_AR
	2	Contamination_VR

Multivariate Tests[a]

Effect		Value	F	Hypothesis df	Error df	Sig.
Project	Pillai's Trace	.003	.090[b]	1.000	30.000	.766
	Wilks' Lambda	.997	.090[b]	1.000	30.000	.766
	Hotelling's Trace	.003	.090[b]	1.000	30.000	.766
	Roy's Largest Root	.003	.090[b]	1.000	30.000	.766
Technology	Pillai's Trace	.324	14.358[b]	1.000	30.000	.001
	Wilks' Lambda	.676	14.358[b]	1.000	30.000	.001
	Hotelling's Trace	.479	14.358[b]	1.000	30.000	.001
	Roy's Largest Root	.479	14.358[b]	1.000	30.000	.001
Project * Technology	Pillai's Trace	.255	10.251[b]	1.000	30.000	.003
	Wilks' Lambda	.745	10.251[b]	1.000	30.000	.003
	Hotelling's Trace	.342	10.251[b]	1.000	30.000	.003
	Roy's Largest Root	.342	10.251[b]	1.000	30.000	.003

a. Design: Intercept
Within Subjects Design: Project + Technology + Project * Technology

b. Exact statistic

Mauchly's Test of Sphericity[a]

MEASURE_1

					Epsilon[b]		
Within Subjects Effect	Mauchly's W	Approx. Chi-Square	df	Sig.	Greenhouse-Geisser	Huynh-Feldt	Lower-bound
Project	1.000	.000	0	.	1.000	1.000	1.000

Within Subjects Effect	Mauchly's W	Approx. Chi-Square	df	Sig.	Epsilon[b] Greenhouse-Geisser	Huynh-Feldt	Lower-bound
Technology	1.000	.000	0	.	1.000	1.000	1.000
Project * Technology	1.000	.000	0	.	1.000	1.000	1.000

Tests the null hypothesis that the error covariance matrix of the orthonormalized transformed dependent variables is proportional to an identity matrix.

a. Design: Intercept
Within Subjects Design: Project + Technology + Project * Technology

b. May be used to adjust the degrees of freedom for the averaged tests of significance. Corrected tests are displayed in the Tests of Within-Subjects Effects table.

Tests of Within-Subjects Effects

MEASURE_1

Source		Type III Sum of Squares	df	Mean Square	F	Sig.
Project	Sphericity Assumed	9.879	1	9.879	.090	.766
	Greenhouse-Geisser	9.879	1.000	9.879	.090	.766
	Huynh-Feldt	9.879	1.000	9.879	.090	.766
	Lower-bound	9.879	1.000	9.879	.090	.766
Error(Project)	Sphericity Assumed	3293.871	30	109.796		
	Greenhouse-Geisser	3293.871	30.000	109.796		
	Huynh-Feldt	3293.871	30.000	109.796		
	Lower-bound	3293.871	30.000	109.796		
Technology	Sphericity Assumed	500.008	1	500.008	14.358	.001
	Greenhouse-Geisser	500.008	1.000	500.008	14.358	.001
	Huynh-Feldt	500.008	1.000	500.008	14.358	.001
	Lower-bound	500.008	1.000	500.008	14.358	.001
Error(Technology)	Sphericity Assumed	1044.742	30	34.825		
	Greenhouse-Geisser	1044.742	30.000	34.825		
	Huynh-Feldt	1044.742	30.000	34.825		
	Lower-bound	1044.742	30.000	34.825		
Project * Technology	Sphericity Assumed	276.008	1	276.008	10.251	.003
	Greenhouse-Geisser	276.008	1.000	276.008	10.251	.003
	Huynh-Feldt	276.008	1.000	276.008	10.251	.003
	Lower-bound	276.008	1.000	276.008	10.251	.003
Error(Project*Technology)	Sphericity Assumed	807.742	30	26.925		
	Greenhouse-Geisser	807.742	30.000	26.925		
	Huynh-Feldt	807.742	30.000	26.925		
	Lower-bound	807.742	30.000	26.925		

Tests of Within-Subjects Contrasts

MEASURE_1

Source	Project	Technology	Type III Sum of Squares	df	Mean Square	F	Sig.
Project	Linear		9.879	1	9.879	.090	.766
Error(Project)	Linear		3293.871	30	109.796		

Source	Project	Technology	Type III Sum of Squares	df	Mean Square	F	Sig.
Technology		Linear	500.008	1	500.008	14.358	.001
Error(Technology)		Linear	1044.742	30	34.825		
Project * Technology	Linear	Linear	276.008	1	276.008	10.251	.003
Error(Project*Technology)	Linear	Linear	807.742	30	26.925		

Tests of Between-Subjects Effects

MEASURE_1
Average

Source	Type III Sum of Squares	df	Mean Square	F	Sig.
Intercept	110940.976	1	110940.976	407.783	.000
Error	8161.774	30	272.059		

Proportions

Notes

Contents		
Output Created		04-SEP-2024 18:06:40
Comments		
Input	Active Dataset	DataSet2
	Filter	<none>
	Weight	<none>
	Split File	<none>
	N of Rows in Working Data File	31
Missing Value Handling	Definition of Missing	User-defined missing values are treated as missing.
	Cases Used	Each statistic is based on all valid data for the analysis variable(s) used in computing the statistic.
Weight Handling		not applicable
Syntax		PROPORTIONS<br>/PAIREDSAMPLES Spider_AR Contamination_AR WITH Spider_VR Contamination_VR (PAIRED) TESTTYPES=MIDP<br>MCNEMAR CITYPES=BONETT_PRICE NEWCOMBE WALD<br>/SUCCESS VALUE=LAST<br>/CRITERIA CILEVEL=95<br>/MISSING SCOPE=ANALYSIS USERMISSING=EXCLUDE.
Resources	Processor Time	0 00:00:00.04
	Elapsed Time	0 00:00:00.00

Paired-Samples Proportions Statistics

		Successes	Trials	Proportion	Asymptotic Standard Error
Pair 1	Spider_AR = 48	1	31	.032	.177
	Spider_VR = 49	1	31	.032	.177
Pair 2	Contamination_AR = 42	1	31	.032	.177
	Contamination_VR = 49	1	31	.032	.177

Paired-Samples Proportions Confidence Intervals

	Interval Type	Difference in Proportions	Asymptotic Standard Error	95% Confidence Interval of the Difference	
				Lower	Upper
Pair 1: Spider_AR - Spider_VR	Bonett-Price	.000	.046	-.119	.119
	Newcombe	.000	.046	-.133	.133

	Interval Type	Difference in Proportions	Asymptotic Standard Error	95% Confidence Interval of the Difference	
				Lower	Upper
Pair 2: Contamination_AR - Contamination_VR	Wald	.000	.046	-.089	.089
	Bonett-Price	.000	.046	-.119	.119
	Newcombe	.000	.046	-.133	.133
	Wald	.000	.046	-.089	.089

Paired-Samples Proportions Tests

	Test Type	Difference in Proportions	Asymptotic Standard Error	Z	Significance	
					One-Sided p	Two-Sided p
Pair 1: Spider_AR - Spider_VR	Mid-p Adjusted Binomial	.000	.046		.500	1.000
	McNemar	.000	.046	.000	.500	1.000
Pair 2: Contamination_AR - Contamination_VR	Mid-p Adjusted Binomial	.000	.046		.500	1.000
	McNemar	.000	.046	.000	.500	1.000

T-Test

Notes

Contents		
Output Created		04-SEP-2024 18:07:29
Comments		
Input	Active Dataset	DataSet2
	Filter	<none>
	Weight	<none>
	Split File	<none>
	N of Rows in Working Data File	31
Missing Value Handling	Definition of Missing	User defined missing values are treated as missing.
	Cases Used	Statistics for each analysis are based on the cases with no missing or out-of-range data for any variable in the analysis.
Syntax		T-TEST PAIRS=Spider_AR Contamination_AR WITH Spider_VR Contamination_VR (PAIRED)<br>/ES DISPLAY(TRUE) STANDARDIZER(SD)<br>/CRITERIA=CI(.9500)<br>/MISSING=ANALYSIS.
Resources	Processor Time	0 00:00:00.02
	Elapsed Time	0 00:00:00.00

Paired Samples Statistics

		Mean	N	Std. Deviation	Std. Error Mean
Pair 1	Spider_AR	29.68	31	9.758	1.753
	Spider_VR	30.71	31	10.143	1.822
Pair 2	Contamination_AR	26.13	31	10.941	1.965
	Contamination_VR	33.13	31	11.215	2.014

Paired Samples Correlations

		N	Correlation	Significance	
				One-Sided p	Two-Sided p
Pair 1	Spider_AR & Spider_VR	31	.688	.000	.000

		N	Correlation	Significance One-Sided p	Significance Two-Sided p
Pair 2	Contamination_AR & Contamination_VR	31	.750	.000	.000

Paired Samples Test

		Paired Differences Mean	Std. Deviation	Std. Error Mean	95% Confidence Interval of the Difference Lower	Upper	t	df
Pair 1	Spider_AR - Spider_VR	-1.032	7.872	1.414	-3.920	1.855	-.730	30
Pair 2	Contamination_AR - Contamination_VR	-7.000	7.844	1.409	-9.877	-4.123	-4.968	30

		Significance One-Sided p	Two-Sided p
Pair 1	Spider_AR - Spider_VR	.235	.471
Pair 2	Contamination_AR - Contamination_VR	.000	.000

Paired Samples Effect Sizes

			Standardizer[a]	Point Estimate	95% Confidence Interval Lower	Upper
Pair 1	Spider_AR - Spider_VR	Cohen's d	7.872	-.131	-.484	.224
		Hedges' correction	8.076	-.128	-.471	.218
Pair 2	Contamination_AR - Contamination_VR	Cohen's d	7.844	-.892	-1.305	-.469
		Hedges' correction	8.047	-.870	-1.272	-.458

a. The denominator used in estimating the effect sizes. \nCohen's d uses the sample standard deviation of the mean difference. \nHedges' correction uses the sample standard deviation of the mean difference, plus a correction factor.
