# Supplementary material for: Comparing experience intensity of AR and VR for contrasting phobia stimuli
Source: Virtual Real. 2026 Aug 11;30(4):205. doi: 10.1007/s10055-026-01450-y (PMC13748273; doi:10.1007/s10055-026-01450-y)
Supplement: Supplementary file 2 [file 10055_2026_1450_MOESM3_ESM.docx]

### Interview Transcripts

**Interviewee: Participant 1
Date: July 26, 2024, 1:33 PM**

**Interviewer:** Our first question is about your main feelings while completing a task in VR compared to AR. You can discuss the realism and immersion while doing the exposure tasks in both AR projects. You can talk about either project one or two and compare them, but mainly focus on the comparison between VR and AR.

**Participant 1:** I think with the spider, I felt more immersed. I was more engaged with the environment and the spider object because of the technology. In VR, I felt like I was in somewhere I didn’t belong. So when the spider appeared, like on the table in my real environment, I felt it was more real. I could look closer, maybe pinch it or put it on my hand. That made it feel immersive.

But I also feel the spider was more realistic in the AR environment. It's common for us to see insects in daily life, classrooms, bedrooms, so it feels natural. But in VR, the spider is just sitting on my bed or chair, static. It didn’t feel as real.

For project two, my feelings were a bit the opposite. The dustbin is less likely to appear in a classroom, so in AR, it didn’t feel very natural. But in the VR version, where the bin was placed somewhere like on a street, I felt more immersed. So, I think it depends on the objects and scenarios.

**Interviewer:** Did you encounter any difficulties while interacting with the two environments? These can be mental, emotional, or technical.

**Participant 1:** Yes. For me, I’m a bit scared of insects like cockroaches and especially spiders. In project one, I found it mentally hard to complete the tasks. Even though I knew it wasn’t real, I still didn’t want to pick up the spider or put it on my hand. I had a strong emotional response, especially because in AR the spider was moving. It felt scary.

In project two, the emotional reaction was not that strong. I don’t think a bin can hurt me, so I was more comfortable completing the task. There was a bit of technical lag, though, which was an issue. But overall, I felt more interested in the VR environment in project two. There was gravity, some interaction, it was enjoyable, even though it was just rubbish.

**Interviewer:** Do you think AR and VR are suitable tools for phobia therapy?

**Participant 1:** Yes, definitely. I wouldn’t want to deal with a real spider in real life. So whether it's AR or VR, I prefer using these technologies. They help reduce fear by repeated exposure. I think this method can be a good alternative to traditional therapy.

If I were a real patient, I’d prefer a gradual process. For example, I could start with a static spider that’s not so scary, then move on to a moving spider, then use AR where the spider appears in my surroundings. The final step could be interacting with a real spider. So, yes, it should be gradual.

**Interviewer:** What do you think about the prototype? Is there anything that would make it more realistic?

**Participant 1:** I think it’s quite perfect now. The interaction works well and feels complete. But maybe the spider could appear from somewhere more realistic, like suddenly appearing from a window, rather than just being on my bed.

For project two, maybe sound could be added. Like if the bin is broken or something, hearing some things or sound effects might make it feel more real.

**Interviewer:** Do you have any additional feedback?

**Participant 1:** Maybe just about the process. If I were a patient in a clinic, I’d wear a headset and start the therapy. I think it would be better if there was more explanation about what I’m doing and why. Especially in VR, I might feel disconnected from my physical therapist. In AR, it’s better because the therapist can be with me.

It would also help if the therapist could enter the same virtual or augmented space. That way, I wouldn’t feel alone. Also, the instructions should explain the benefits: why I’m doing this task, how it will help me. Maybe explain that there are 12 sessions, the first is about looking, then interacting, and so on. So I feel like I’m getting closer to overcoming my fear step-by-step.

**Interviewer:** Great. That’s a really good point. Thank you for your feedback and participation.

**Interviewee: Participant 2**

**Date: July 20, 2024, 2:32 PM**

**Interviewer:** Could you describe your feelings during the task experience in both the VR and AR projects? In particular, how did you perceive realism and immersion?

**Participant 2:** VR felt more realistic. Maybe it’s because, in AR, I was still able to see my actual classroom, so subconsciously I kept thinking that dirty things like that shouldn’t exist in a classroom. As for the spider, in VR, the immersion made it feel more frightening. I really felt like I was inside that closed environment. There was even a moment of being chased.

In AR, even though I could see my real hands and know it was me interacting with the spider, like picking it up or having it crawl on my hand, there was sometimes clipping, like the spider would pass through objects unnaturally. But in VR, that clipping issue wasn’t as noticeable because my hands weren’t fully visible. They were represented by transparent blue models. This actually enhanced the feeling of being present in that virtual space and allowed my imagination to take over more strongly.

**Interviewer:** Were there any difficulties during your interactions in the VR and AR environments, either psychological or technical?

**Participant 2:** I felt a sense of relief when I took off the headset, like I didn’t have to deal with those dirty things anymore. With the spider, I felt that the realism of the dirty objects was actually stronger than the realism of the spider. The spider didn’t have visible fur or a lifelike presence. So in AR, it wasn’t too scary, but in VR, the environment made the dirty things, like the trash bin, feel very real and disgusting.

**Interviewer:** Did these kinds of immersive tasks trigger strong emotional responses for you?

**Participant 2:** Looking at the dirty objects reminded me of times when I had to clean or throw stuff into a trash bin that gross feeling. In AR, I felt an urge to clean it all up. But in VR, the urge was more about escaping the environment.

**Interviewer:** Between the two technologies, which do you prefer for exposure therapy, and why?

**Participant 2:** Personally, I prefer VR for both projects. For something like dirty trash bins, placing them in a virtual yet realistic setting and using something like a backpack to interact with them felt effective. In AR, if something dirty appears in a clean classroom, it feels out of place and reduces the realism. So I think both tasks were more suitable for VR.

**Interviewer:** Do you have any personal reasons for preferring VR, or is it mainly about realism?

**Participant 2:** It’s mostly because I felt the environments in VR were more convincing. But I think if spider exposure therapy was done in the patient’s actual bedroom using AR, that might be more effective, because the spider appears in a real, familiar space. But for the dirty object therapy, I still think VR is better.

**Interviewer:** Do you have any feedback on the design or interaction of the environments?

**Participant 2:** If the space was larger, it might have improved things. Overall nothing major disrupted my experience.

**Interviewer:** Any final thoughts or feedback about the overall experience?

**Participant 2:** No, that’s all.

**Interviewer:** Great, thank you for your participation. The interview and experiment are now complete.

**Interviewee: Participant 3
Date: July 19, 2024, 1:16PM**

**Interviewer:** Could you describe your feelings during the task experience in both the VR and AR projects? In particular, how did you perceive realism and immersion?

**Participant 3:** In the AR spider task, even though I wasn’t afraid, I did feel psychological discomfort. When I picked up the spider, it genuinely felt like its antennae were tickling my fingers. I knew it wasn’t real, but the emotional and sensory response was still very strong. Especially when it crawled across the back of my hand, there was a definite tingling sensation. Even when the spider was completely static, I still sensed a certain level of threat from it, as if it could start moving at any moment. This feeling made me uneasy, as though it were real

In VR, the spider felt more naturally integrated into the environment. There wasn’t any visual mismatch, so it didn’t feel out of place. Compared to AR, I felt braver in VR. The fear was less intense. I still had that slight tingling sensation when the spider crawled on me, but overall, the psychological discomfort was lower.

I think part of the reduced fear in VR came from the clear awareness that I was in a virtual environment. It’s kind of like watching a horror movie. You know it’s fake, so your emotional response is controlled. In contrast, AR blurred the boundary between reality and virtual. There was always this subtle feeling that the spider might actually be real, which made the sensory stimulation much stronger. In that sense, AR felt more immersive, more psychologically impactful. So for spider phobia therapy, I think AR could be more effective for those who are looking for a more intense, reality-like experience. But for people who are less confident or more hesitant, VR might be a better starting point.

**Interviewer:** How about your experiences during the contamination-related tasks?

**Participant 3:** In VR, interacting with objects like cans or bottles didn’t cause much reaction. But things like fruit peels and apples gave me a stronger psychological sense of disgust. The red dots on them reminded me of flies.

The VR environment, kind of broken down and dirty, made everything feel more convincing. In contrast, doing the same task in a clean classroom with AR felt less effective. Seeing trash in a clean space doesn’t feel real. Even though I knew everything was virtual, picking up rubbish in VR felt more immersive.

Also, I didn’t feel much relief from throwing objects away. The uncomfortable part was already over by the time I picked them up. Maybe if the trash looked more rotten or had a slimy texture, I’d want to get rid of it faster. With the spider, it was different. Since it was moving, I had a stronger urge to shake it off.

**Interviewer:** Between AR and VR, which do you prefer for these types of exposure therapies?

**Participant 3:** I think it depends on the type of treatment. For spider-related exposure, I believe AR is more effective. But for the contamination task, I think VR works better. AR gives patients the feeling that a spider has truly appeared in their real environment. It makes them feel like the spider actually exists in front of them, which enhances the sense of realism. In contrast, for the contamination task, when it's presented in AR, in a clean environment like a classroom, it doesn't feel convincing. The dirty objects seem out of place, and I instinctively know they don't belong there. That weakens the emotional reaction.

So I think it’s important to make patients feel that the virtual elements truly belong in the space. The virtual objects need to match the physical environment to feel real. That’s why I think VR is more suitable for the contamination scenario. It allows for a controlled, convincingly dirty setting.

**Interviewer:** Did either of the scenarios trigger any strong emotional responses?

**Participant 3:** Yes, especially in the spider task using AR. That felt more emotionally difficult. The spider’s presence felt more real in AR than in VR. I think that added realism made it harder to ignore.

**Interviewer:** Is there anything else that contributed to your emotional reactions during the experience?

**Participant 3:** Yes. I think the lack of psychological preparation before the experiment played a part. I wasn’t told exactly what to expect, so the reactions felt stronger because I was entering the unknown. Also, imagining a spider appearing in my own bedroom — where I eat and sleep — would be even more disturbing than the lab setting. That would make the AR experience feel much more personal and intense.

**Interviewee: Participant 4
Date: July 19, 2024, 3:25 PM**

**Interviewer:** Could you describe your experience during the contamination task in both AR and VR?

**Participant 4:** In the AR environment, I found it easier to convince myself that the trash might actually be real, and I was more willing to engage with it. But in VR, I was very aware that everything was virtual. Psychologically, it didn’t feel as disgusting.

In AR, there was a clear contrast. A clean classroom with a filthy trash bin. That contrast created a kind of "illusionary smell" and a stronger sense of revulsion. In VR, it all just looked like virtual objects, and the same feeling didn’t come through.

When I was asked to interact with the contaminated items, there was a slight difference. Surprisingly, I was actually a bit more reluctant to touch things in VR. But overall, the emotional response was stronger in AR.

**Interviewer:** Overall, do you have a preference between AR and VR?

**Participant 4:** I generally prefer AR for both projects. As I play a lot of games, during the VR experience I was very aware that it was completely detached from reality. I approached it with a gamer's mindset, so I didn't focus as much on my feelings, but rather on my curiosity about the VR experience and how the game would progress.

If the experience is meant to be purposeful, I think AR is more suitable. In VR, there are too many distracting environmental factors, like walls or the room layout, which can pull attention away from the task.

Also, if AR is used in familiar environments like my apartment or dorm room, the sense of immersion and safety is stronger. The emotional impact is much higher compared to a virtual space that I don’t recognize. A swarm of spiders running at me in my bedroom would be a completely different experience than seeing them in a random virtual world.

That said, AR is more limited because it's very location-dependent. It works well only in suitable environments. VR doesn’t have this problem, but VR does require ensuring the space is physically safe. I’m always a little worried about tripping over something. AR feels less risky in that sense.

Another point is that AR gives a stronger sense of embodiment. When a spider crawls on me in AR, it feels like it’s crawling on my actual body. In VR, it's just a virtual hand, and the effect isn't as strong.

Today’s setup was actually quite suitable for AR. But if the lighting had been different, such as under harsh natural sunlight, the spider might have looked out of place. So choosing the right lighting is important.

AR uses real-world lighting and effects to re-render virtual models, so it seems like things, like a virtual spider, can get a bit blurry, which actually makes them look more real.

**Interviewer:** What are your thoughts on the design of the prototype and interaction methods?

**Participant 4:** Whether it's AR or VR, what matters is how real the experience feels to the user. For instance, a real spider doesn’t just drop straight down. It might crawl around your arm, which is pretty disgusting. Or if it’s garbage, stepping on it might make a squishy sound, or it might feel limp when you pick it up. The more realistic, the better. The goal is to keep users from realizing it’s fake to avoid that “I’m just playing a game” feeling.

I'd also like more interactive options. For example, if I slap an object, maybe it collapses into a ball or disintegrates. Right now, the grasping method isn’t very realistic like when using an open palm to grab the surface of an object. Improving this would help. Even with AR's immersion, the lack of tactile realism breaks the illusion.

Sound feedback is also important. If something is supposed to be dirty, I’d expect flies buzzing around or something that makes it more disgusting. That would motivate me to throw the trash away and close the lid, and I’d probably feel a sense of relief after doing it.

Right now, I still dislike the trash, and I would clean it up. But the motivation isn’t as strong as it could be.

**Interviewer:** Do you think the system feels too artificial or lacks certain sensory feedback?

**Participant 4:** Yes, it does feel a bit too virtual. I often found myself wanting to hear some kind of sound, because obviously we can’t have smell feedback, but I expected at least some audio. A little sound feedback would help the visuals and audio feel more connected. Like, when a plastic bottle hits the trash can, I should hear a light clunk. Or when I pick up a plastic bag, I should hear the crinkling sound. That kind of detail would make the experience more cohesive and believable.

**Interviewer:** How would you compare the two projects?

**Participant 4:** The spider task felt more passive. Even though the spider might crawl on my hand, once it starts moving, it kind of just does its thing. I don’t have much agency in that experience. Personally, I’d prefer something like a swarm of spiders rushing at me. It would feel more intense.

In contrast, the contamination task felt more active. I had a sense of control. I was the one picking up the trash. For someone with contamination anxiety or OCD tendencies, they probably don’t need a game to tell them to clean. They’d just do it on their own.

**Interviewee: Participant 5
Date: July 22, 2024, 1:37 PM**

**Interviewer:** So could you please describe your main feelings while completing the task in VR compared to AR?

**Participant 5:** I think if it's to do with items, AR would be better for living things. But if it's to do with contamination, it's about the environment. So I would say VR for that one would be a bit more suited.

**Interviewer:** OK, so for the preference, you choose AR for the spider project?

**Participant 5:** Yes, AR for spiders. And VR for contamination. Yes.

**Interviewer:** How did you like the realism and immersion?

**Participant 5:** Immersion was really good. I just think that version was really well done. In terms of my preference between the two, it's mainly because contamination fears are closely tied to the environment. How things are arranged and perceived around you. I feel that when you're forced to face a stimulus, like a dirty environment, it amplifies the emotional response and discomfort. That’s why the VR one felt more effective.

Whereas with spiders, if you see one in your actual living area, it feels much more real and threatening, more exposure-based. That’s why AR, with its ability to use the camera to recognize and place the spider in your real-world environment, made the experience feel more real in that scenario.

**Interviewer:** Did you encounter any difficulties when you did these experiments?

**Participant 5:** No, I'm all good. But I do have resistance or avoidance trying to do certain things, mental barriers and challenges, I would say. Because I do have fears of contamination and spiders, but it's not that extreme. I'm okay with performing all the tasks you asked me to. It's just like, having the spider crawl on my hands, I was a bit "Oh... I'm not sure about that." And also, picking things out of the bin. I wasn’t OK with that. But picking things like the trash outside of the bins and putting it in, I was okay with that.

**Interviewer:** So did you feel a strong emotional response?

**Participant 5:** Yes, more so in the contamination one than the spider one. The spider one I felt, although it was a fear. It was a bit more fun, because there was a lot of movement, getting to interact. In the contamination one, it was a bit more like “ugh,” because of the space and how the bins were. I feel like if you were to add more elements like flies and those kinds of things, it would make it feel a bit more real. I was a bit more reluctant to do those.

**Interviewer:** Could you compare your feelings between the two scenarios? Are there many differences?

**Participant 5:** So with the spider one, I think my fear wasn’t too bad in terms of interaction. But my fears are more to do with. Like, if it's bigger, if it crawls onto me. I’m not interacting with it, but I'm seeing it there and then it crawls toward me. I find that a bit more scary.

But in comparison to the contamination one, because it’s to do with your environment and you being in that situation, I would say the fear and the emotion was a bit higher in the contamination one than the spider one.

**Interviewer:** Do you have any suggestions for this prototype or the experiments?

**Participant 5:** Yeah. So I would definitely say a bit more visuals with the bins, like adding flies. Other sensory things like sounds. For example, when you're interacting with the bins, when you shut it, add those kinds of sounds. Or like when I grab a bag, it will make a sound.

Also, if you're able to, I'm not sure if you are, but with the contamination one, if you're able to grab it while you see it… For example, if I grab it, I pinch it here, it should look like I’m holding it like this, not like this.

**Interviewer:** On the surface, right?

**Participant 5:** Yes. So that would make it a bit more real.

With the spider one, I think it’s more to do with when people fear spiders. It’s not just about approaching them. It’s more like, what if the spider attacks you? So, how do you feel if you approach the spider, versus how do you feel if the spider approaches you?

I think that’s more scary. For people with very extreme arachnophobia, it might not be good, maybe too much, but it could be effective. So I think the interaction was great, it’s just the very minor details to improve.

**Interviewer:** Do you have any additional feedback you’d like to share?

**Participant 5:** All good. I think if you have different levels of exposure therapy, then maybe change up the setting, not just VR or AR, but within the setting itself.

For example, if you can change the way things look. Like, the contamination one was very blue, and that gives a cold sense of feeling. But what if you change it to be a bit lighter or warmer in colour? Then it might feel different.

**Interviewer:** Thank you so much for your time and insights.

**Interviewee: Participant 6**

**Date: July 25, 2024, 1:13PM**

**Interviewer:** When you were completing the task, how did you feel about the differences between AR and VR? Specifically, what about the realism and immersion?

**Participant 6:** The difference is quite big. VR completely beats AR. In VR, I could perceive subtle movements, but in AR, I overlooked them. AR had only one scene. VR felt more connected to daily life or at least more in tune with the intended setting. For me, in the second task (contamination), there was still some technical issue like vibration and delay happening constantly.

**Interviewer:** How was the immersion for you?

**Participant 6:** Since I was wearing the headset, I subconsciously kept telling myself it’s fake. My mind kept saying “this is fake,” so I felt that whatever happened, any kind of behavior or state, couldn't really affect me.

There were various technical and hardware limitations that made it feel less real. But if you couldn’t see the external environment, it would feel more real and less mentally distracting. Oh, and it (spider) didn’t feel like real-life movement. Whether it was movement speed or appearance, it just didn’t feel natural.

**Interviewer:** And the second one?

**Participant 6:** The second VR environment’s ground was done really well. But overall, the VR spider scene had a much larger area than the trash bin VR scene.

So it might depend on the person. People who feel discomfort in small, enclosed spaces might find it harder to adjust. For me, it wasn’t really the “dirtiness,” but more the physical sensation of movement in 3D space. That made me feel uncomfortable.

**Interviewer:** Did you have any strong emotional reactions during the process?

**Participant 6:** The spider was actually really cute. I didn’t feel scared. In the second VR environment, it was a smaller space, with dirt all over the floor. VR gave a much stronger feeling of dirtiness than AR.

**Interviewer:** How would you compare the spider and the trash bin experiences? Any emotional differences?

**Participant 6:** The spider could move, which gave me more of a reaction and made it more interesting. The trash bin task was just picking things up…not very exciting. In AR, such dirty objects wouldn’t normally appear in a classroom. But in VR, they were placed realistically on the floor. That VR environment felt like a real trash station. I even felt like I was dirty myself. Even though I wasn’t physically dirtied, and I didn’t touch other parts of the environment, it still felt like I was surrounded by filth. The atmosphere itself made me feel that way.

**Interviewer:** Do you think AR and VR are suitable tools for exposure therapy?

**Participant 6:** AR and VR can be useful tools for exposure therapy, as long as the person isn’t affected by motion sickness or vision-related issues.

**Interviewer:** Any difference?

**Participant 6:** I think they were about the same for the first task. I clearly remember that the second scene with the trash bin didn’t feel as real. The virtual bed and toothbrush didn’t feel convincing.

**Interviewer:** So was it more about the scene setting?

**Participant 6:** Yes, the environmental realism made the difference. In both cases, the tasks were just grabbing spiders or trash. But if I had actually been lying in a real bed, grabbing a spider would have felt more immersive. That’s when I would’ve felt like the VR “seeped into” reality.

**Interviewer:** Do you have any suggestions for improving the environment?

**Participant 6:** Maybe add more dirty elements in the second scene, make it feel even filthier. Things that enhance that kind of sensory reaction. The ceiling was low. If it were more enclosed, the feeling would have been stronger. Right now, the focus isn’t really on the ceiling. But if it had something like tomato sauce splattered and dripping down. Especially if it happened right when I looked up. That would have made it feel more real.

**Interviewees: Participant 7 & Participant 8**

**Date: July 25, 2024, 4:41 PM**

**Interviewer:** Based on what we experienced earlier, is there anything you’d like to add, especially regarding the differences between AR and VR when completing the tasks? Any comparisons about realism or immersion?

**Participant 7:** It’s not about the virtual model itself, but how it's placed in the environment. If AR lacks that surrounding environmental modeling, it can actually make the object stand out more but in a way that breaks the illusion. So I feel that in both projects, VR gave a stronger sense of immersion and realism.

**Participant 8:** Yes. When you're in a modeled world like VR, everything feels cohesive. But with AR, because you're comparing the virtual object to the real environment, it makes the object stand out more. It feels more obviously like a model. In VR, it's easier to convince yourself that everything belongs to the same dimension or operates on the same baseline, which makes the experience feel more consistent.

**Interviewer:** Did you experience any strong emotional reactions?

**Participant 7:** I would say yes, particularly in VR. I personally dislike desaturated or grayish tones. The environment gave me a stronger sense of discomfort. And…the spider was dynamic, so the experience felt more vivid. Its movement made it feel more real and the interaction more intense. The trash items were static, which made the sense of interaction feel weaker.

**Participant 8:** I think part of it is that I personally don’t like the appearance of trash. This reminds me of the corner of the narrow space beneath my accommodation, where there was a huge, dirty, and cluttered rubbish bin. So during that scene, I felt a stronger emotional reaction. Also, in VR, you mentally position yourself within that dimension or environment. So it ends up feeling more real.

**Interviewer:** Do you think both AR and VR are suitable tools for exposure therapy? Or do you think one is more appropriate than the other depending on the condition?

**Participant 7:** I think both are suitable, but it depends on the context. For the spider task, AR could work better — especially if the exposure happens in the patient's own room, like a familiar everyday environment. That might make the experience feel more real.

**Participant 8:** Yes, and for contamination, VR might be more effective. The environment can be made to feel more consistently dirty, whereas AR is more limited by the real physical setting.

**Interviewer:** Thank you both. That was very helpful.

**Interviewee: Participant 9
Date: July 26, 2024, 10:27 AM**

**Interviewer:** Could you describe your feelings when completing the spider-related tasks in both VR and AR?

**Participant 9:** In the VR environment, I was fully placed into a closed virtual space with no real-world elements around me. The VR simulation had a lot of detail like the texture of the desk and the spider’s movements which enhanced the realism of the task. As the spider approached my hand, I almost felt as if I could physically feel it, and the environment became increasingly realistic.

In AR, although the spider also looked very real, I could still see the actual surroundings like parts of the classroom. That connection to the real environment slightly reduced immersion and fear, because I knew I was still in a familiar space. The AR experience was more relaxed overall, as it allowed me to interact with both virtual and physical elements at the same time.

**Interviewer:** And what about the contamination task?

**Participant 9:** In VR, the immersion was extremely strong. It really felt like I was standing in a dirty, trash-filled place. That realism triggered a strong emotional reaction. I felt very uncomfortable and wanted to leave. The visuals in the VR trash task were so realistic that I could almost smell the garbage. While the smell was only in my mind, it heightened my discomfort.

In AR, the experience was quite different. I could still see the real classroom, along with the virtual trash and trash bin. That gave me a kind of "safe distance," because I knew I wasn’t actually in a filthy space. While this reduced the realism, it also reduced the psychological burden.

**Interviewer:** Did you find the two environments technically difficult to use?

**Participant 9:** I didn’t encounter any major difficulties in either environment. Neither was particularly scary or technically challenging for me. Even in VR, where the garbage made me very uncomfortable, the controls and interaction were straightforward. Compared to the trash task, the spider task felt simpler.

I do have some level of mysophobia, and I really don’t like handling dirty-looking trash bins. But my biggest fear is actually snakes. If the task involved snakes, I think I would have panicked much more.

**Interviewer:** Were there any emotional responses you’d like to highlight?

**Participant 9:** The VR task, especially the trash one, gave me a very strong emotional response. I felt an urgent need to exit the environment. But with the spider task, I gradually relaxed. The spiders weren’t real, and they couldn’t bite me. That helped clear up some misconceptions I had and made the experience easier to manage.

**Interviewer:** Which version did you prefer overall?

**Participant 9:** I preferred VR in both tasks. The immersion was much stronger, especially for the garbage task. It made the experience feel more realistic. Over the course of the 30-minute session, I felt I made some progress in handling spiders. I haven’t fully overcome the fear, but I’ve started adapting to it.

**Interviewer:** Do you have any final thoughts on the VR and AR experiences?

**Participant 9:** This was a valuable experience. It helped me understand the strengths and weaknesses of both technologies. VR has a clear advantage in creating fully immersive environments, but it can also bring psychological pressure if the setting is too uncomfortable. The good thing is if it becomes too much you can just take off the headset and return to reality. That’s a unique advantage of VR when helping people face their fears.

**Interviewee: Participant 10
Date: July 26, 2024, 4:18PM**

**Interviewer:** Could you describe your emotional experiences in both the VR and AR experiments?

**Participant 10:** I found VR to be more immersive because you feel like you are really in that environment. For the spider task, I truly felt like I was in a room that was not very pleasant. There was a dirty bed with a spider on it and spider webs in the corners of the room. I literally felt immersed in that environment. The spider felt more detailed in VR. Maybe not more realistic overall, but I could clearly see more visual detail on the spider in VR compared to AR.

**Interviewer:** And in the second experiment?

**Participant 10:** Again, I found VR to be very immersive. It felt like I was actually in a dirty environment, with old walls and graffiti all around. In AR, I was aware that I was in a classroom. The bin did not belong there, and I could tell that. That awareness reduced the emotional impact of the experience in AR for the second project.

**Interviewer:** Did you encounter any difficulties during the tasks, either technical or emotional?

**Participant 10:** Technically, the interface with the card and the radio buttons was not very intuitive. Pinching or clicking was a bit confusing at first. But after learning how to interact with the objects, like using the pinch-and-pick gesture, it became easier.

Mentally, there was a bit of hesitation at the beginning. I had never picked up objects in VR before. I had only explored VR environments. But once I understood how it worked, it was fine. There was a learning curve, but it got easier with use.

**Interviewer:** Did you feel any strong emotional reactions?

**Participant 10:** Yes, I felt strong emotions in both tasks. With the spider, although I am not usually scared of spiders in real life, picking one up was scary. You could see its legs moving. Even though I could not feel the touch, just seeing it crawl in my hand was unsettling.

With the bin, I felt like I should not be touching or opening the lid or putting my hand inside. Even though I knew it was not real, it still felt wrong to do that.

**Interviewer:** How would you compare your feelings between the spider and contamination scenarios?

**Participant 10:** They are quite different scenarios, but the emotional responses were similar. With the spider, I was fine as long as it was moving on its own. But picking it up made me hesitate. With the bin, it was the same. Picking up dirty items or putting my hand inside made me feel uncomfortable. Even though I knew both were virtual, I still did not want to do it.

**Interviewer:** Do you think VR and AR are suitable for exposure therapy?

**Participant 10:** Yes, I think VR can be a great tool for helping people overcome fear. It gives a very strong sense of being in a different environment. AR feels more detached because you are still physically in your real environment.

It depends on the context. For example, I remember from a lab session, there was a VR game where you had to walk on a rope at the top of a mountain. Even though I knew I was in a room, I still felt fear. That showed me how powerful VR can be in creating emotional responses, even for things like fear of heights.

So VR has great potential for therapy. AR might work well for something like spider phobia. You could use your own room as the setting. But for other types of exposure, I think VR is more effective.

**Interviewer:** Any final feedback?

**Participant 10:** Overall, it was a different and meaningful experience. The experiment’s goal came through clearly. Especially in the VR environment, I felt much more immersed. The AR experience was also fine, but in the second experiment I was very aware that I was still in a classroom. So when I saw the dustbin, I knew it did not belong there. That made it feel less real. For the spider task, I would say the experiences were quite similar between AR and VR. Both technologies have their own potential. One may work better for certain scenarios, and the other for different ones.

**Interviewee: Participant 11**

**Date: July 28, 2024, 11:50 AM**

**Interviewer:** Could you describe your main feelings when completing tasks in AR and VR? In particular, how did immersion and realism differ between the two?

**Participant 11:** For the spider task, I didn’t feel a big difference between AR and VR. The spider’s presence felt reasonable in both environments. The room in VR made sense, and in AR, the spider being on a table or bed also felt natural. Since the spider was small and there weren’t too many of them, the difference wasn’t very strong.

But for the garbage task, VR felt much more realistic. It matched the dirty environment better, and I had a stronger reaction. I felt that if I touched something, my hands would become dirty. Picking up a banana peel, for example, made me uncomfortable.

**Interviewer:** Did you encounter any difficulties while interacting with the environments? This can include psychological discomfort or technical challenges.

**Participant 11:** I hadn’t really used this kind of technology before. So, when I was still trying to understand the interface and something suddenly popped up on the screen, I felt a bit disconnected.

In terms of psychological resistance, yes, when I was asked to pick up garbage or interact with it, I really didn’t want to. I still felt like if I touched it, my hand would become dirty. That was hard for me to accept.

**Interviewer:** And how did that compare to the spider task?

**Participant 11:** The emotional impact from the spider was not as strong as the trash. Maybe because I am more used to the idea of spiders. Also, since there’s no actual touch sensation in the headset, it didn’t feel very real.

In real life, when a bug falls on me, I can feel it crawling. But here, even if a spider appears on my hand, it doesn’t give me that same feeling. So it wasn’t very frightening.

**Interviewer:** So between the two scenarios, the garbage task had a bigger emotional impact?

**Participant 11:** Yes, definitely. The trash environment gave me a stronger sense of discomfort. The dirty and oppressive atmosphere of the VR environment made me want to leave immediately.

**Interviewer:** Do you think AR and VR are suitable technologies for exposure therapy?

**Participant 11:** I think it depends on the individual’s tolerance. For someone like me, VR is fine. But for people who are extremely afraid of spiders, they might prefer AR because in AR you can still see another person next to you. That might bring comfort and a sense of safety.

On the other hand, being alone in a VR space might increase immersion and potentially make the therapy more effective. But if the fear is too strong, it could be overwhelming. So it really depends on the person.

**Interviewer:** Do you think these technologies have any specific advantages or disadvantages?

**Participant 11:** One main drawback is the lack of real tactile sensation. For example, when a spider is crawling on your hand, you don’t feel it. That makes the experience less scary.

Also, I think it would be better if there were more types of spiders. In real life, we don’t just encounter one kind. If you only use one model repeatedly, it becomes predictable. But if you have different kinds, it keeps the fear active. If someone gets used to one spider model, they might stop feeling afraid, but seeing a new one might trigger their fear again.

**Interviewee: Participant 12 
Date: July 28, 2024, 1:19 PM**

**Interviewer:** Can you describe your overall feelings during the interactions? This can include immersion, realism, and how VR and AR compared for you.

**Participant 12:** The spider felt very real, although I knew it couldn't be. But if it were to move, I think I would really be scared. Especially if it gets close, I start to feel a kind of trypophobia like goosebumps.

For the spider phobia scenario, I think using AR would make the fear stronger. When it appears in the real environment and you have to grab it, the realism increases. In VR, grabbing a spider feels more like playing a game. But in AR, because spiders are real animals and part of your world, the fear is more intense.

For the garbage task, I didn’t feel much in either AR or VR. That task lacked a strong sensory presence for me. Garbage in real life has smell, not just visuals or touch. Without smell, it just felt like another object. It was not very special.

**Interviewer:** Aside from the brief black screen issue, did you encounter any difficulties during the interaction? These could be emotional or technical.

**Participant 12:** In the spider scenario, the act of picking it up gave me a bit of fear. If there were a lot of spiders, like hundreds of them, that would have been really terrifying.

I think the spider gave me a stronger emotional impact, especially when I had to grab it and its legs were moving. That moment felt very real in AR. In VR, the image quality was less sharp. The spider was not as clear, maybe due to resolution or pixel limitations.

**Interviewer:** Compared to the garbage scenario, would you say the spider task caused a stronger emotional reaction?

**Participant 12:** Yes, much stronger. The garbage task didn’t make me feel much overall.

**Interviewer:** In the preference question, you chose AR for the spider task and VR for the garbage task. Can you explain why?

**Participant 12:** For the garbage task, VR gave me a very dirty environment. It felt like a trash site. But in AR, since we were in a library, seeing garbage there felt wrong. My subconscious rejected it because it did not belong in that setting.

So VR was more convincing for garbage. But for the spider, AR was more effective because it placed the spider in a more realistic and believable context.

**Interviewer:** Do you think AR and VR are suitable tools for virtual exposure therapy? Do they have specific strengths or weaknesses?

**Participant 12:** I think it depends on the treatment goal.

For something like spider phobia that happens in everyday environments, AR is better. It puts the feared object into your real surroundings. But if the fear is related to extreme environments, like volcanoes or cliffs, then VR is more suitable because you can create those environments virtually.

So if it’s a fear rooted in daily life, AR might be more useful. If it’s a fear that requires a special setting, VR works better.

**Interviewer:** In terms of contamination or OCD-related exposure, do you think AR would work well?

**Participant 12:** For me personally, neither worked very well for that. With spiders, yes. But for contamination, I think the sense of smell plays a much bigger role. That was missing. Maybe if the AR scenario used dirty clothes or more relatable dirty things, it would feel more effective. But otherwise, I did not feel very affected.

**Interviewer:** Is there anything you would suggest to improve the prototypes?

**Participant 12:** I think the garbage environment in VR was done quite well. There were piles of trash in the corners and the design made it feel a little dirty. But in the AR version, especially in the library, it didn’t feel dirty at all. So environment really matters.

If technically possible, adding smell or more physical sensations would help. For example, if the spider could be paired with vibration feedback when grabbed, that would make it more real. Right now, when I picked up the spider, there was no feedback. But if the controller vibrated, like in a gamepad, that would feel more convincing.

Also, for longer interactions, it would help if the spider felt like it was crawling or moving continuously. That would make it feel even more realistic.

**Interviewer:** Would you prefer controller-based interaction or hand tracking for this kind of therapy?

**Participant 12:** Since this is exposure therapy, I think hand tracking is better. Even though you lose some of the tactile feedback, it feels more natural. It fits the goal of helping someone face their fear more directly.

**Interviewee: Participant 13
Date: July 31, 2024, 1:05 PM**

**Interviewer:** Can you describe your overall experience when performing tasks in AR and VR? In particular, how did you feel in terms of immersion, realism, and presence?

**Participant 13:** VR felt more realistic overall. In AR, I experienced a kind of disconnection or mismatch between the virtual elements and the real world.

For the spider task, I think the feeling in VR was more intense. It is a small thing in real life, and in that kind of simulated environment, even though I knew it was just a model, it felt more convincing than in VR. In contrast, for the garbage task, VR felt more real than AR.

**Interviewer:** Did you encounter any difficulties during the interaction process, either technical or psychological?

**Participant 13:** Technically, sometimes the objects were difficult to grab. There was some stuttering or delay when I tried to move them.

Psychologically, I had no fear during the spider task because I’m not afraid of spiders in general. But in the garbage task, when you set two trash bins and had garbage scattered all over the floor, I did feel a strong urge to clean it up. That impulse was present in both AR and VR, but it wasn’t about realism. It just felt like a game mechanic… something to complete.

**Interviewer:** Did you have any noticeable emotional reactions?

**Participant 13:** Maybe a small one with the spider, but I didn’t consciously notice it. With the garbage, it was more obvious. The VR version made me feel more resistant to interacting with it. Possibly because it felt more real, I didn’t want to clean it up.

**Interviewer:** Do you remember your preferences in the questionnaire?

**Participant 13:** I selected VR for both tasks. But now that I think about it, I realize I said earlier that AR felt more real for the spider task. So I may have been inconsistent. After reflecting, I think my feelings have shifted a bit since the experiment.

**Interviewer:** Would you say VR and AR are suitable tools for exposure therapy? Do they have any specific advantages or limitations?

**Participant 13:** I think it depends on the situation. For something like spider phobia, which is closer to daily life and more likely to happen in familiar environments, AR might be more appropriate. Because with AR, you can see your real surroundings, and if something like a spider fits into that setting, it makes the fear more believable.

For more complex or extreme environments, like a garbage dump or other less familiar places, VR is probably more suitable. If the virtual content doesn’t match the physical space, like placing trash in a classroom, it just feels fake. So the match between the virtual object and the real setting matters a lot.

**Interviewer:** So overall, you think both AR and VR have potential for exposure therapy, depending on the type of fear?

**Participant 13:** Yes, definitely. While reviewing literature, I came across a study on fear of heights. VR was used in that case because you obviously can’t simulate height realistically in AR. You can’t actually put someone at the top of a mountain, but you can create that experience in VR, and it feels convincing.

So for extreme or impossible-to-replicate scenarios, VR works better. But for everyday fears things that might really appear in your environment. AR could be more effective.

**Interviewer:** Do you have any feedback or suggestions for improving the prototype or experiment design?

**Participant 13:** I think the rendering quality could be better, though I understand time is limited. Also, the soda bottles were way too large almost comically oversized. The spider’s crawling movement was very well done, though. That part felt quite convincing. The VR room scene like the home interior could be more realistic overall.

For the contaminated task in VR, it would have felt more immersive if the setting had really looked like a dump. If the models had been placed next to real-looking trash bins in a believable location, that would have improved immersion. Putting them in a clean classroom made them feel artificial and out of place. The mismatch between the object and the environment reduces the realism.

**Interviewee: Participant 14
Date: July 31, 2024, 2:02PM**

**Interviewer:** Could you briefly describe your overall feelings when completing the tasks in AR and VR, particularly in terms of realism and immersion?

**Participant 14:** When I first saw the spider in AR, it felt like it was actually in the room. But I think if the spider looked even more natural, it would have been better. When I touched my desk in AR and a spider popped out, I could feel something happening. It was like I felt something in my hand even though there was no real tactile sensation. I couldn't describe it exactly, but it wasn’t like pinching.

In VR, I felt more like there were lots of spiders around me. It felt like they could crawl on my legs or on me. That’s what made me feel afraid. I had this itchy feeling, like they were really crawling on me. That’s why VR felt more real to me. When the spiders were scattered in the virtual room, I didn’t know where they came from, and that uncertainty added to the realism.

**Interviewer**: What about the contamination task?

**Participant 14:** I felt more pressure and stress in VR for that one. The room was closed off and I had no space to move. It felt like a small area filled with trash. It looked very real. Even when I threw some garbage into the bin, the place didn’t feel cleaner. It made me uncomfortable. In AR, when I picked something up, it felt more like relief like I was done and cleaning was starting. So it felt lighter.

**Interviewer:** Did you encounter any difficulties, either mental or physical?

**Participant 14:** Physically, I didn’t feel any discomfort or difficulty. But mentally, I definitely felt stress, especially in VR. It gave me a strong emotional reaction.

**Interviewer:** How would you compare the spider and contamination scenarios? Did they trigger different kinds of feelings?

**Participant 14:** Yes, they were very different. The contamination task affected me mentally. I felt a lot of pressure. The spider task was more of a physical feeling like itchiness, something on my skin. That was the difference.

**Interviewer:** Do you think AR and VR are suitable tools for exposure therapy?

**Participant 14:** Yes, I think both can work well. For me, VR was more immersive. It felt more competitive in the sense that it challenged me emotionally.

**Interviewer:** In the preference questions, you selected VR for both tasks. Could you explain why?

**Participant 14:** I think VR just felt more real overall. In AR, I could touch the table, but in VR, when I tried to touch a chair, there wasn’t actually a chair there. I feel like if the chair had been real, it would have felt more convincing. Otherwise, you know it’s not completely real.

**Interviewer:** Do you have any additional feedback about the prototype or the overall process?

**Participant 14:** I think the process was smooth. I liked the way we switched between VR and AR and then back again. It really let me feel the differences. The only thing was in VR, when I couldn’t touch the virtual chair, it left me unsure about the realism. But besides that, it was a good experience.

**Interviewee: Participant 15
Date: July 31, 2024, 3:11 PM**

**Interviewer:** Can you describe your overall feelings in the AR and VR environments, focusing on immersion, realism, or general experience?

**Participant 15:** I think the technology was really good overall. I especially liked the spider project because it’s actually something I encounter in daily life. Living in the UK, I feel like learning to coexist with spiders is necessary.

Through AR, the spider seemed less frightening, and although I would probably still avoid physically touching it in real life, this helped reduce my fear. For people who have psychological trauma around spiders, this would definitely help.

With the contamination project, I didn't really feel strong emotions. I think it’s a good concept, maybe more helpful for encouraging people to maintain cleanliness habits. But for me personally, I didn’t feel a therapeutic effect because I don’t have OCD. Maybe for people who are extremely sensitive to dirt in their home, it would be more useful.

**Interviewer:** Did you experience any difficulties when interacting with the environments, either mental or physical?

**Participant 15:** In VR, it was easy to bump into furniture. That was a bit inconvenient. Sometimes, when I tried to pick things up, the object positioning felt a bit off, which made the movement feel strange. Mentally, I felt discomfort in the contaminated VR environment. There were moments when I felt overwhelmed and thought, this is just too much. Like I couldn't fix the mess.

**Interviewer:** Did you feel strong emotional reactions during the tasks?

**Participant 15:** Yes, particularly in the VR trash scene. I felt a strong emotional reaction, like a sense of helplessness.

**Interviewer:** How would you compare the emotional impact of the spider versus the contamination scenario?

**Participant 15:** Both were uncomfortable, but the spider felt a bit cuter and more manageable. The trash felt more unsafe. You feel a bit unsafe, especially with the graffiti-covered walls. It feels like I’m trapped in a strange place. It gave a sense of insecurity.

**Interviewer:** What are the respective strengths and weaknesses of VR and AR in your opinion?

**Participant 15:** VR is great for building immersive environments. But because everything is virtual, it can feel separate from real life. AR is more grounded in reality. But that also means the virtual objects need to be extremely detailed. If the spider model isn’t realistic enough, it will feel fake because the rest of the environment is real. That mismatch breaks immersion. In AR, if the virtual object doesn’t visually or behaviorally match the real setting, the illusion falls apart. But in VR, because everything is virtual, it stays consistent.

**Interviewer:** Do you think VR and AR are suitable tools for exposure therapy based on your experience?

**Participant 15:** Yes, I think they are suitable. I personally didn’t feel very afraid of the spider, so it was manageable. The contamination scene felt a little exaggerated for me. I don’t have OCD, but even I found it a bit overwhelming. For someone who really has severe OCD, that environment might be too intense and uncomfortable.

But overall, I think this kind of exposure therapy through immersive environments is promising. It’s about helping people gradually face what they fear.

**Interviewee: Participant 16**

**Date: August 1, 2024, 11:42**

**Interviewer:** Can you describe your overall experience when interacting with the AR and VR environments? You can focus on aspects like immersion, realism, and how you felt during the interaction.

**Participant 16:** In terms of overall experience and realism, I didn’t feel a huge difference between AR and VR. But in AR, because you’re still seeing the real-world environment and then overlaying virtual objects, psychologically and visually it felt more uncomfortable. For example, in the garbage task, I felt more resistant to the trash when it was placed in my familiar surroundings.

In VR, being placed in a closed, fully virtual space created a stronger sense of immersion and increased the intensity of the emotional experience.

**Interviewer:** Between the two projects, did one of them give you a stronger emotional reaction?

**Participant 16:** Yes, the garbage environment in VR had a stronger emotional impact. The sense of discomfort was higher in that setting. I’m not really scared of spiders, but I am more sensitive to dirty things, so the garbage task made a stronger impression on me.

**Interviewer:** Did you encounter any difficulties during interaction? These can be mental or physical, such as feeling resistant to performing tasks or any issues with the controls.

**Participant 16:** I didn’t experience psychological resistance to doing the tasks themselves. But being in that kind of enclosed space in the VR garbage scene gave me a strong sense of discomfort. It felt like everything around me was dirty, and that environment really shaped my emotional state.

**Interviewer:** Based on your experience, do you think AR and VR are suitable tools for exposure therapy?

**Participant 16:** Yes, I think they can work well. The idea is to gradually expose the user to more intense stimuli so that they slowly become desensitized. If I saw a spider that big in the real world, I would still be afraid. But seeing it in a virtual environment makes it easier to face.

With the garbage task, the visual design and color of the space contributed a lot to the emotional impact. I felt like the atmosphere was heavy, like I was sealed inside a dirty place.

**Interviewer:** Do you think AR and VR each have particular strengths or limitations when used for virtual exposure therapy?

**Participant 16:** I think both are useful. But if you could add more sensory feedback, like smell or touch, that would make the experience much more complete. That kind of multi-sensory stimulation would make it feel more like a real situation and increase the effectiveness of the therapy.

**Interviewee: Participant 17**

**Date: August 2, 2024, 9:49 AM**

**Interviewer:** Could you describe your main feelings during the experience, particularly focusing on immersion, realism, and how AR and VR compared for you?

**Participant 17:** AR felt like a warm-up phase. Before entering the AR or VR experience, I didn’t know what the spider would look like. The AR segment helped me mentally prepare by giving me a preview of the spider.

At first, I felt a little scared, but then I started to observe it and evaluate whether it looked real. I realized it was manageable. When the spider moved, it triggered stronger discomfort than when it stayed still, but it was still within a tolerable range.

In VR, the environment felt very close to real life. The sudden appearance of spiders in such a peaceful and familiar space created a sharp emotional contrast. It felt disruptive and made me want to get rid of them.

The spider on the bed seemed less threatening, maybe because it looked smaller or because I’m used to the idea of seeing bugs on a bed. But when it appeared on a chair, it felt visually more shocking. The model was large, and perhaps because the chair was narrow, the spider felt oversized and more intense.

**Interviewer:** How about the garbage task?

**Participant 17:** The graffiti walls in the VR environment made the garbage look much worse. When surrounded by trash, everything felt dirty. In cleaner environments, the same trash didn’t seem as bad. I was more willing to handle the trash in a tidy space. But in the VR setting, I felt more reluctant, especially when reaching into the bin.

**Interviewer:** Did you experience any difficulties, either mentally or physically?

**Participant 17:** I wouldn’t say there were severe difficulties. Psychologically, I didn’t want to deal with the dirty tasks, but it was still manageable. Physically, no serious obstacles. The system recognized my gestures well and gave immediate feedback, which helped maintain the sense of presence.

**Interviewer:** Did you experience any strong emotional responses during the tasks?

**Participant 17:** Yes, especially when the spider appeared in familiar spaces. If it was just sitting on a table with no environmental context, I didn’t care as much. But when it appeared in a space that looked like my own home, like a desk I use every day, it triggered stronger discomfort.

The garbage scene was also emotionally impactful. In a dirty environment, I felt overwhelmed, like I couldn’t clean everything. It felt like being trapped in a place I couldn’t fix.

**Interviewer:** Between the two projects — spider and contamination — which one do you think is more emotionally stimulating?

**Participant 17:** I think the contamination task is more effective for exposure therapy. It’s easier to design full sensory immersion for trash than for spiders. With spiders, it’s harder to simulate realistic touch or crawling sensations. But visual disgust works well with garbage. The emotional reaction was stronger in the trash environment.

**Interviewer:** In the preference questions, you selected VR for both tasks. Can you explain your reasoning?

**Participant 17:** I think VR is closer to real life in terms of how it creates immersive environments. In AR, I always feel like I’m doing an experiment. The virtual object is overlaid on my real surroundings, and my subconscious keeps reminding me that it’s not real. But in VR, the entire environment supports the illusion. I feel more immersed, like I’m part of the space. That makes the emotional experience stronger.

**Interviewer:** What are the strengths and weaknesses of VR and AR for exposure therapy, based on your experience?

**Participant 17:** VR’s strength is that it provides a complete and believable environment. The weakness is that the user may still recognize it’s a game, especially if the environment doesn’t connect with their personal life. AR feels more experimental. The virtual objects need to be extremely realistic to avoid breaking the illusion. If the models don’t match the real-world setting, it becomes obvious that they’re fake. That makes it harder to build a deep emotional response. However, VR environments can simulate unfamiliar or exaggerated scenarios, which may make users feel less personally responsible or less emotionally attached. If something appears in my personal space, like my desk at home, I feel more responsible to remove it. But if it’s in a virtual alleyway, I can just walk away. The psychological weight differs depending on how familiar the environment feels.

**Interviewer:** Do you think these technologies are suitable for use in exposure therapy?

**Participant 17:** Yes, definitely. The safety aspect is very important. Because it’s not real, users can mentally prepare and gradually face their fears. Even if they’re extremely afraid, the experience can be adjusted in stages. It works especially well as an early phase of therapy, offering visual and behavioral exposure. Later stages could use more tactile or physical elements. This approach makes it easier for people to start the therapy without being overwhelmed from the beginning.

**Interviewee: Participant 18 & Participant 19**

**Date: August 2, 2024, 9:49 AM**

**Interviewer:** Could you describe your main feelings during the experience, particularly regarding the differences between AR and VR in terms of immersion, realism, or overall comfort?

**Participant 18:** I felt more comfortable in the VR scenario, especially with the spiders. They were crawling on the bed and the chair, and that made it feel like their natural habitat. It was immersive and somehow made me feel calmer. I could interact with them, pick them up, and I got used to it faster. In AR, they felt more out of place — like they didn’t belong on the real-world table, so that was more unsettling at first.

**Participant 19:** For me, it was the opposite. In AR, I didn’t feel much because I knew the spiders weren’t real and I could see the real room. But in VR, I felt uneasy.

**Interviewer:** Did you encounter any difficulties while interacting with either environment?

**Participant 18:** Not really. I found it quite intuitive. Maybe at first I didn’t know where to go, but otherwise it was easy to get used to.

**Participant 19:** I did feel some VR sickness, especially in the second scenario. I had to crouch and then stand up repeatedly, and I think that made me feel dizzy. It wasn’t unbearable, but I definitely noticed it.

**Interviewer:** Did you feel any strong emotional responses during the tasks?

**Participant 18:** Yes, especially during the contamination scene in VR. It triggered a strong feeling of discomfort, like being in a really dirty environment. I didn’t want to touch anything. The spider scene was fine, even a bit fun once I got used to it.

**Participant 19:** The contamination room in VR made me feel like I didn’t want to be there. It looked too familiar, like a place I avoid in real life. With the spiders, I didn’t have a strong reaction — they didn’t scare me, maybe because I couldn’t feel them physically.

**Interviewer:** How would you compare your feelings toward the two scenarios — spiders versus contamination?

**Participant 18:** The spider one felt lighter. I could play with them. Contamination was much heavier emotionally, especially in VR, it was a bit overwhelming.

**Participant 19:** Spiders were manageable. The contamination scene, especially in VR, was just uncomfortable. I didn’t want to interact with the trash.

**Interviewer:** Do you think AR and VR are suitable tools for exposure therapy? Any pros or cons?

**Participant 18:** VR works well. Even though I don’t love wearing headsets, I preferred VR in both scenarios because it felt more complete. The environment and stimuli matched better. There was more stimulus, and it felt more effective even though I wasn’t comfortable.

**Participant 19:** I think both are useful, but it’s better to start with AR. Jumping straight into VR might be too intense. AR can serve as a gentler introduction.

**Interviewer:** Do you have any suggestions for improving the prototype or making it feel more immersive or realistic?

**Participant 18:** Definitely more sensory inputs. For example, the garbage should have a smell. The banana peel looked too perfect — it should be rotting. And it would help to actually feel the spider on your hand.

**Interviewer:** Anything else you'd like to share?

**Participant 18:** I really enjoyed the spider part. It reminded me of cultural beliefs — in India, if a ladybug lands on you, it means good luck. Same with grasshoppers. I once had one land on my shoulder and soon after I got a job. So I try to see insects as positive.

**Interviewer:** Thank you both.

**Interviewee: Participant 20**

**Date: August 5, 2024, 11:22**

**Interviewer:** There will be a brief follow-up interview to cover some aspects we haven’t discussed yet. Could you describe your main feelings during the task experience in both VR and AR? You can focus on realism and immersion.

**Participant 20:** Do I need to talk about both?

**Interviewer:** Yes, both would be helpful.

**Participant 20:** I feel like with the spider scenario, it felt more like a video game. It was more of a gameplay experience. But with AR, it felt more like actual exposure therapy. That probably has to do with my personal concerns. In general, I’m more bothered by dirt and bacteria than by spiders. Spiders can be avoided. If you get close to them, they usually run away. But dirt just stays there. I also associate dirt with bad smells. Spiders are just spiders. I don’t blame them for existing. If anything, seeing a spider means I’ve entered their space. With rubbish, the feeling is different. It makes me feel frustrated with people who don’t bother putting it into the bin. I think the emotional context is quite different for spiders versus contamination

**Interviewer:** Did you feel a difference between VR and AR in terms of realism?

**Participant 20:** Yes, I think it was more realistic in AR. In VR, to make it feel truly realistic, the environment would have to be very detailed and resource-intensive. Otherwise, everything feels artificial — not just the background, but the spider or rubbish too. When everything is obviously computer-generated, the main object ends up feeling more fake as well. With moving objects like spiders, they feel more natural because you don’t focus on the detail. It’s like spiders appeared somewhat blurry in AR. This blurriness reduced its artificial quality, making it seem more threatening. But if the whole environment is artificial, that affects your perception.

**Interviewer:** Did you encounter any difficulties, either mentally or physically?

**Participant 20:** With AR, sometimes objects get stuck in textures. For example, a spider might run through your hand. It’s not exactly difficult, but it feels like an uncanny valley effect. In VR, you can only see your hands, not your arms. If your hand drifts outside the view, you have to move it quickly to make it visible again. Because the hand is visually detached from the arm, it’s harder to understand its spatial position. In AR, you can see your whole arm, so it’s easier to control.

**Interviewer:** Did you have any strong emotional responses during the tasks?

**Participant 20:** Yes. The dirty environment triggered an emotional reaction. I didn’t feel much with the spiders, probably because I’ve gotten used to cartoon-style spiders from playing video games. Last year, I played Hogwarts Legacy, and there were lots of spiders. That game even has an arachnophobia mode, where if you choose it, the spiders wear roller skates on their legs and look more silly. It’s kind of like exposure therapy through gameplay. I’ve seen so many animated spiders that they don’t affect me as much now.

**Interviewer:** Do you think AR and VR are suitable technologies for exposure therapy?

**Participant 20:** I think AR would be preferable. You can see the objects in environments that you’re already familiar with. That makes it more effective. If you can recreate situations that actually occur in your daily life, it feels more relevant. Also, AR tends to have fewer issues with motion sickness. In VR, everything is shaking slightly, and that makes it more noticeable. In AR, small glitches may happen too, but they’re easier to ignore.

**Interviewer:** That’s everything. Thank you very much for your participation.

**Interviewee: Participant 21**

**Date: August 7, 2024, 11:58 AM**

**Interviewer:** Could you describe your main feelings while completing the tasks, especially focusing on immersion, realism, or your overall experience in AR and VR?

**Participant 21:** In the first experiment, especially from the scale of interaction, I felt the AR version was very well done. For example, tapping to generate the spider felt very realistic. When the spider started to move, it gave me a strong sense of realism, like there were truly two spiders crawling in front of me.

In the VR scene, although immersive, the environment felt slightly less real. That could be due to the material textures. It was easy to distinguish from real-life settings, but it did help pull me into a more immersive atmosphere. The spider animation in VR was a bit repetitive, and sometimes it clipped through objects, which reduced the realism. In contrast, AR allowed me to see the spider crawling on the table more clearly and intuitively, so I’d say AR had an advantage in simulating spiders.

**Interviewer:** And how about the second experiment?

**Participant 21:** The AR environment initially gave me a stronger impact because it felt novel. Although I often clean and take out trash in real life, seeing polluted liquids on the floor so close felt very real. Still, I didn’t strongly resist cleaning up the trash.

However, in this second experiment, I thought the VR scene did a better job. The dirty floor and the environment created a strong immersive effect. The integration of environment and task had a one-plus-one-greater-than-two effect. So, overall, I think VR worked better for the contamination scene.

**Interviewer:** Did you experience any difficulties during the interactions, whether mentally or physically?

**Participant 21:** Personally, I didn’t face much difficulty. I’m not afraid of spiders, so I actually found it an interesting experience. But for people who do have spider phobia, I think it could be very helpful and effective. For the contamination task, I had no physical or mental resistance. I’m the kind of person who doesn’t mind cleaning things up, so I didn’t have any strong avoidance response. But I do recognize that the VR environment

gave a more intense emotional feedback.

**Interviewer:** Based on your experience, do you think AR and VR are suitable tools for exposure therapy? Any pros or cons?

**Participant 21:** I think they’re definitely suitable. In real life, you can’t just put a patient with spider phobia into a spider-filled room, and plush toys aren’t the same. This technology offers a very effective alternative. For contamination, you’d have to worry about actual hygiene and safety in real environments, so using VR or AR is a great way to bypass those issues while still achieving the emotional impact.

However, there are a few concerns. First, VR can cause motion sickness. Second, individual patients have different psychological tolerances. For example, I’m not sure if your system has a limit on how many spiders can be spawned. If someone keeps clicking and spiders pile up, that could overwhelm them instead of helping them gradually desensitize.

So that should be considered, especially for people with lower tolerance. I also think adding more interactivity or tactile feedback would help enhance realism. Overall, it’s a very promising direction.

**Interviewer:** Do you think AR is more suitable for some treatments, and VR for others?

**Participant 21:** Yes, it depends. People have different preferences and physical capabilities. Some may find AR more accessible — everyone has a phone, but not everyone owns a VR headset. So from a practical and commercial perspective, AR might be more universal.

VR, on the other hand, gives stronger environmental control. If you’re targeting exposure to dirty environments, VR can simulate those more effectively.

For spiders, AR might be better in the early stages. It’s easier to accept one small spider on a real table than dozens in a VR room. So ideally, start with AR and gradually move to VR as part of a step-by-step process.

**Interviewer:** So, for something like spider phobia, you’d start with AR and move to VR?

**Participant 21:** Yes, especially since spiders can move unpredictably. That uncertainty causes a lot of fear. In AR, you can start with one small, static spider. Once the patient is used to it, you can gradually introduce movement, and then shift to a VR environment with more immersion.

This would mirror a progressive desensitization approach. For contamination, however, the visual impact of a dirty environment matters more, so VR might be more effective from the beginning.

**Interviewer:** Thank you. That was incredibly detailed and insightful.

**Interviewee: Participant 22
Date: August 8, 2024, 9:02AM**

**Interviewer:** Could you describe your main feelings during this experience, especially focusing on realism and immersion? You can compare AR and VR or Project One and Project Two.

**Participant 22:** I think there’s a slight difference between realism and immersion. In my mind, AR always feels more realistic because the environment is real — I can feel the objects and get tactile feedback. But in terms of immersion, sometimes VR feels more immersive, especially in the contamination condition. The VR environment felt more appropriate, like a real scene for rubbish. In contrast, seeing two rubbish bins in a classroom felt odd and less believable.

**Interviewer:** That makes sense. Could you also explain your answer to the question about feeling relief when throwing away rubbish?

**Participant 22:** Sure. Even though the VR condition felt more realistic and immersive, I actually felt more relief in the AR condition. In AR, since I was still in the real classroom, the rubbish felt out of place — so throwing it away gave me a sense of cleansing. In the VR scene, rubbish belonged there. It was a natural part of that environment, so throwing it away didn’t feel like much of a change.

**Interviewer:** Did you encounter any difficulties while interacting with either environment, mentally or physically?

**Participant 22:** Not really. In the spider condition, I focused mostly on the spiders in both AR and VR. Maybe because they were the only objects around. I tried interacting with other things in VR but quickly lost interest. In the contamination scenario, I felt more freedom to explore.

**Interviewer:** Did you experience any strong emotional response during either task?

**Participant 22:** Not particularly. Maybe because I don’t have strong feelings about spiders or rubbish. But the spiders did give me a bit of a reaction, especially when they were moving. I think that response would have been stronger if the visual fidelity were higher. If I could see the small details, like their eyes and body textures, it might have felt more real and maybe more unsettling.

**Interviewer:** Do you think AR and VR are suitable tools for exposure therapy?

**Participant 22:** I think so, but I’m not completely sure. For therapy, I assume emotional responses matter, and I didn’t feel very strong ones myself. Maybe because I’m not that fearful of spiders or dirt. But I did a project once for mixed reality, where I wanted to simulate public speaking fear. It’s a similar idea — placing someone in an audience situation in VR to simulate performance anxiety. The challenge was making people look and react realistically. That’s the key: if people or spiders don’t look real, it won’t work as well. But the idea has potential, especially if gradually introducing elements like real audience members or reactive environments.

**Interviewee: Participant 23
Date: August 9, 2024, 8:54AM**

**Interviewer:** Could you share your main feelings about the experience? You can focus on VR, talk about the differences between the two projects, and focus on realism and immersion — just your overall feelings.

**Participant 23:** To my observation, I found the AR version of the spider project more preferable. It gave me the most realistic feeling. In AR, I can see my own hands, which makes it easier to believe that I am actually holding a spider, rather than just controlling a virtual object. This interaction made me feel more afraid because it felt closer to a real-life experience.

For the contamination task, I couldn’t really feel anything in either AR or VR. As I explained before, I was expecting a texture, something that made the rubbish feel dirty when I touched it. That was missing when I was grabbing rubbish or touching the bin lid. In a real dirty environment, more senses are involved. There should be a funky smell, a tactile feeling that makes you not want to touch things, and the sense that your hands might get dirty. These aspects were missing, so I couldn’t feel either environment was realistic in the contamination project.

**Interviewer:** Did you encounter any difficulties during the process, mentally or physically?

**Participant 23:** In the second project, I felt very dizzy — especially in the VR environment. It was a bit better in AR, but the dizziness was still there, and I’m still feeling it now. I’m not sure what caused it. In the first project, I didn’t feel as dizzy. Maybe it was due to the size of the environment or the visual attention required. Other than that, there wasn’t much physical strain or difficulty.

**Interviewer:** Do you think AR and VR are suitable tools for exposure therapy?

**Participant 23:** Yes, but I would suggest combining both. For spiders, I prefer AR. As I mentioned before, when I can see something in my familiar environment, it makes it feel more real. That’s why I would choose AR for spider phobia therapy. For contamination, I’m not so sure — maybe neither version felt real enough due to the lack of sensory elements.

**Interviewer:** So if we were to develop an immersive experience for exposure therapy, you would prefer AR over VR?

**Participant 23:** Yes, I would.

**Participant 23:** For VR, maybe adding more sensory elements could help — like what the professor did in his fire evacuation project. He added a smell component by burning something, which made the environment feel more real. I’m not saying to burn things here, but maybe simulating that kind of sensation could help in VR. That’s about it.

**Interviewer:** That’s great, thank you for your participation!

**Interviewee: Participant 24**

**Date: August 8, 2024, 3:53PM**

**Interviewer:** Could you describe your main feelings when interacting with the AR and VR environments? You can focus on immersion, realism, or your general experience. Feel free to discuss both the spider and contamination tasks.

**Participant 24:** When I first interacted with the spider, I was a bit hesitant and scared. When I tried to touch it and saw that it moved, I really didn’t want to touch it and wanted to throw it away quickly. Then, in the more realistic VR version, visually it felt more real, but since there was no physical contact, the tactile feeling remained the same. However, visually and psychologically, the feeling changed. As for the contamination task, regardless of the environment, it all felt the same to me — dirty is dirty, no matter where it is.

**Interviewer:** In the preference question, you chose VR for both spiders and contamination. Could you explain why?

**Participant 24:** VR felt more immersive and realistic. It simulates a whole new environment. In AR, you’re reminded that this is not real, since your real-world surroundings are still there. It’s less immersive, while VR is more convincing.

**Interviewer:** Did you encounter any difficulties during the experience, either mentally or physically?

**Participant 24:** Not really. Since the first environment I experienced was AR, it reminded me that it’s not real, which helped me mentally prepare. If I had started directly with VR, I think it would’ve been more difficult. The gradual transition helped me ease into it. I brought the experience from AR into VR, and I wasn’t as emotionally overwhelmed in VR.

**Interviewer:** So you felt stronger emotional responses in VR?

**Participant 24:** Yes. VR felt closer to real life. I could associate it with past experiences, like being at home or next to a real trash bin. Visually, it triggered memories and imagination. But other senses like touch were missing. If the liquid had a feeling, or the trash had weight, it would feel more realistic.

**Interviewer:** Between spiders and contamination, which one gave you a stronger reaction?

**Participant 24:** Spiders in real life come in many shapes and sizes. But in the VR, they all looked the same, so I felt like they were fake. The trash in the contamination scene was more varied and realistic. That made the contamination scene more convincing overall.

**Interviewer:** Based on your experience, do you think AR and VR are suitable tools for virtual exposure therapy?

**Participant 24:** Yes, I think they are. But it depends on the person. Some might be too scared, so it’s important to talk to them first and understand their personality. Then we can decide whether to start with VR or begin gradually with AR.

**Interviewee: Participant 25**

**Date: August 9, 2024, 12:12PM**

**Interviewer:** Could you describe your main feelings about the experience? You can compare AR and VR, or compare project one and two. You may focus on realism, immersion, or any differences between the two technologies.

**Participant 25:** For the spider task, I felt that the AR aspect was more realistic because I could see the room we were in and see the spiders interacting with that environment. For example, seeing the spider respawn on the table and fall off made it feel very realistic and immersive. In the VR space, the VR room didn’t really match this room. I was still spawning spiders on the table, but in VR it appeared on the chairs. That made me feel less connected to the VR room. If, in the VR room, the bed was aligned with the physical table and I could touch it as if it were the table, that would have made the experience more immersive.

From a phobia therapy perspective, I think AR would be better because I get to interact with the spiders in a safe space — my real room. That allows me to develop a better relationship with my fear. VR can also be helpful since it creates a safe, controlled environment, but I’m not sure how effective it would be emotionally.

For the contamination task, VR was nice. If there had been audio or smell, it would have been more immersive. The AR version was not as immersive, because the interaction with the room itself was lacking. In the spider scenario, the web was a nice touch, but in the contamination one, the room didn’t add much to the AR experience. I think the environment did not contribute enough to the AR contamination experience.

**Interviewer:** Did you encounter any difficulties during the process, mentally or physically?

**Participant 25:** No, not really.

**Interviewer:** Did you experience any strong emotional responses?

**Participant 25:** Yes, with the spider. When it came toward me, I instinctively stepped back. I didn’t feel that with the contamination task, since it was all static, dirt just lying around. It didn’t evoke emotion. But the spider was moving, interacting. It wasn’t just a static 3D model. The motion added emotional engagement.

**Interviewer:** Based on your experience, do you think AR and VR are suitable for this kind of therapy?

**Participant 25:** Yes, I see potential, especially for spiders. I’m not so sure about the contamination part, but the spider experience was very effective. The interaction was important. Spiders crawling on my hand, falling off, walking around. That made it engaging.

**Interviewer:** Do you think these technologies have any pros or cons?

**Participant 25:** For AR, the advantage is that it allows me to face my fear in a safe space — my own room. That helps build a secure understanding of the fear. In that scenario, I’m not sure VR contributes as well. But VR has potential if you want to create a situation that doesn’t exist in real life. For example, a haunted house with big spiders jumping out from corners. I can’t experience that in daily life. VR can offer a setting to confront a more intense level of fear. That’s something AR cannot easily simulate.

**Interviewer:** Do you have any suggestions for improvement?

**Participant 25:** I think adding more environmental elements like sound or smell could help make the experience more realistic. Especially in VR, creating those kinds of details could be very effective.

**Interviewer:** Thank you very much.

**Interviewee: Participant 26**

**Date: August 9, 2024, 1:34PM**

**Interviewer:** Could you describe your main feelings about this experience? You can talk about the technologies or the differences between project one and two. You can also focus on realism or immersion.

**Participant 26:** I think it's kind of interesting, to be honest. I never thought exposure therapy could be done using virtual technology because I always thought it had to involve real spiders. I have spider phobia, and I would never try real exposure therapy — that would be terrifying. I can’t even stand the idea of spiders crawling on my body. So in terms of the spider project, it still felt realistic. But for the second one — the contamination task — I didn't feel much emotionally. It looked fake, and because I knew it was fake, it didn’t trigger any strong feelings. Compared to the spider scenario, the emotional response was very different.

**Interviewer:** How did you feel about the two technologies, especially in the spider task?

**Participant 26:** When you're actually in the room using AR, it kind of levels up the terror. You’re in your own real environment, and then the spider shows up — that feels more personal. I remember when you asked me to touch it, I just wanted to get a lid or a fan to push it away. I felt like when I was really in the simulation, it became more realistic.

**Interviewer:** Do you think AR and VR are suitable tools for exposure therapy?

**Participant 26:** I think it depends on how severe the phobia is. For me, I feel my spider phobia is quite serious, so starting with something easier like AR makes sense. Once I get used to it, I can move on to VR. I don’t think it’s about choosing between AR or VR — it’s about gradually leveling up. Start with AR, then move on to VR for further treatment.

**Interviewer:** Do you see pros and cons in using these two technologies for virtual exposure?

**Participant 26:** Yes, I think it’s a good method. Instead of touching a real spider, you’re training your brain to get used to the fear. Even though I knew it was fake, I was still terrified. So imagine if it were a real spider — I wouldn’t be able to handle that. This kind of setup is a very responsive and controlled way to do therapy.

**Interviewer:** Were there any difficulties?

**Participant 26:** I felt a bit dizzy during the second task. The visual quality wasn’t very high, so it made me uncomfortable. I think these technologies still need improvement, especially in terms of quality and realism, but overall I see their value in exposure therapy.

**Interviewee: Participant 27
Date: August 9, 2024, 2:13PM**

**Interviewer:** Could you describe your main feelings throughout this experience? You can talk about the differences between AR and VR, or between the spider and contamination scenarios. You can also focus on the realism and immersion.

**Participant 27:** If I had to summarize directly, I’d say VR felt more suitable and more effective in achieving the intended therapeutic goals — for both scenarios.

**Interviewer:** Both the spider and contamination tasks?

**Participant 27:** Yes, for both. I personally think VR works better because when it comes to psychological therapy, entering a fully virtual environment tends to be more effective. AR still relies on integrating with the real-world environment, while VR allows you to immerse in a completely fabricated space. That creates more room for psychological influence.

**Interviewer:** So you're saying VR creates a stronger sense of immersion and therefore has a stronger emotional impact?

**Participant 27:** Yes, exactly.

**Interviewer:** Did you encounter any difficulties during the experience? Either mentally or physically?

**Participant 27:** Not really, no. Emotionally I was fine. Physically, I had been sitting and looking at my laptop for a long time earlier, so I was already feeling a bit tired. But not related to the experiment itself.

**Interviewer:** Did you feel any emotional responses during the experience? For example, surprise, discomfort, or fear?

**Participant 27:**
Yes, I felt it more clearly in VR. My emotional engagement was stronger in VR than in AR.

**Interviewer:** Why do you think that is?

**Participant 27:** Well, mostly because of the immersive feeling. Maybe other reasons too, but I can’t think of them right now.

**Interviewer:** That’s fine. So do you think both AR and VR can be suitable for exposure therapy?

**Participant 27:** Yes, but ideally they should be used in a staged or progressive way. If I had to pick just one, I think VR is more practical and effective for treatment.

**Interviewer:** What would you say are some strengths or weaknesses of AR and VR in this therapy context?

**Participant 27:** Compared to seeing a real spider or being in a genuinely dirty place, both AR and VR can serve as effective transitional tools — like a bridge. I think your concept is definitely feasible.

**Interviewer:** Between the two technologies, are there certain advantages VR holds over AR, or vice versa?

**Participant 27:** Honestly, I feel like the spider in the simulation wasn’t scary enough. The issue wasn’t the technology itself, but the scene design. It didn’t feel intense enough to trigger a strong reaction.

**Interviewer:** So the limitation was more in the content than the tech?

**Participant 27:** Yes. I’m probably also a more realism-driven participant. I know it's a simulation, so that awareness reduces my emotional response.

**Interviewer:** Do you have any suggestions or feedback for improving the prototype?

**Participant 27:** The spider could be designed to feel more threatening. Recently, I’ve been playing some horror games, and they really know how to create fear. For example, even just black silhouettes with sharp, jagged shapes can be very effective.

**Interviewer:** So you think adding horror elements or combining different emotional triggers like in horror games could increase the impact?

**Participant 27:** Definitely. Horror games often build tension through atmosphere, not just visuals. It’s the unknown that makes things scary — like darkness, or subtle signs that something bad is coming. If we could borrow from that to build up the fear response here, it would be more emotionally powerful.

**Interviewee: Participant 28
Date: August 11, 2024, 11:43AM**

**Interviewer:** Could you briefly describe your main feelings about this experience? You can talk about the technologies, the difference between project one and two and focus on things like realism or immersion.

**Participant 28:** For Project One — the spider task — it felt like a bigger trigger for me. I think for people like me, the fear of spiders isn’t really about the act of touching them, but more about how they look. Just staring at them or watching them move already freaks me out. So for the spider project, I had a stronger fear reaction.
For Project Two — the contamination task — it was a lot easier for me. What usually bothers me about dirty environments is the smell, but since that wasn’t present here, I didn’t feel much. I knew it wasn’t real, so grabbing things didn’t really matter to me emotionally.

**Interviewer:** Did you feel any difference between AR and VR in each project?

**Participant 28:** Yes. For both projects, I felt that AR was a bit more intense. Because it’s happening in your actual environment — for example, imagine there’s a spider right in front of you on your own floor — it just feels more real and scarier. It gives a stronger emotional reaction.

**Interviewer:** Did you encounter any difficulties during the process — mentally or physically?

**Participant 28:** Yes, only during the spider project. That was mentally difficult. I felt a strong emotional response.

**Interviewer:** Do you think AR and VR are suitable tools for exposure therapy?

**Participant 28:** Yes, definitely. If you want to simulate these kinds of environments in real life, it would be difficult and expensive. Plus, if someone is really afraid of these things, they could react too strongly — even dangerously — in a real setting. But in AR or VR, you can just take off the headset if something feels overwhelming, so it's safer.

**Interviewer:** Do you think each technology has its own advantages for different scenarios?

**Participant 28:** Yes. AR depends a lot on your real environment — you need to choose an appropriate space. VR just needs an empty room, and you can create any environment. But for VR, modeling the environment really well is important. If it’s not realistic, it loses immersion.

For AR, it’s easier to make things feel real. You don’t need to design the entire environment — the walls, the floor — just make the items look convincing. But if the assets are too large or detailed, it can become buggy or laggy.

**Interviewer:** So would you say AR is better for everyday scenarios like spiders or contamination, and VR better for larger, fantasy-based ones?

**Participant 28:** Exactly. AR works well when the scenario is part of daily life — like a spider in your room. But VR is better for experiences like a haunted house, or being in total darkness. The challenge with VR is performance — if the scene is too big or detailed, it’s harder to optimize and run smoothly.

**Interviewer:** Any additional feedback?

**Participant 28:** Yes — maybe you could add more physical sensation. For example, if you could feel the spider crawling on you, that would make it way more realistic. A lot of people who are scared of bugs imagine them crawling on their skin, so simulating that would make it more effective.

**Interviewer:** That’s very helpful. Thank you for participating!

**Interviewee: Participant 29
Date: August 11, 2024, 1:10PM**

P29 stated, "In VR, I approached the spider more with a gaming mindset, feeling like it was more of a challenge than an actual threat."

**Interviewer:** Now I have some follow-up questions. First, could you briefly describe your main feelings during the experience? You can talk about the difference between AR and VR, or the difference between Project One and Two. Feel free to focus on aspects like immersion or realism.

**Participant 29:** Okay. For the first project, which involved spiders, I actually preferred the AR version a bit more. I think for treatments involving psychological conditions like depression or specific phobias, which are serious topics, it’s better to have someone guiding the experience. In the AR setting, I could clearly see where the facilitator was, so I didn’t feel overly scared.

I don’t have this phobia myself, so I wasn’t really terrified, but I imagine that for someone who does, being in a closed-off, fully immersive VR space might be too much.

In terms of realism, both technologies were good. But I preferred AR for the spider scenario. As for technical details, the AR seemed to have higher resolution and clarity. Overall, I think it could be helpful for patients to try AR first.

Since I knew the spiders weren’t real, I was more willing to try interacting with them. So I think AR can help users take the first step in desensitization.

**Interviewer:** What about the second scenario?

**Participant 29:** For that one, I preferred VR. Even though there were some issues like frame rate or resolution, the immersive experience felt stronger. The surrounding trash and mess in the VR environment felt more like an actual polluted space than the scattered trash in the AR version. The benefit of AR is that you can still see your real surroundings, which reduces nervousness. But in terms of immersive quality, I’d say VR was more powerful. I think AR might have more potential in terms of future technology, but currently, VR offers a deeper sense of immersion, especially when the environment is richly rendered.

**Interviewer:** Did you encounter any difficulties during the process?

**Participant 29:** Not really. Basic interactions like clicking or touching worked fine. Maybe some UI elements like buttons could be slightly larger, but I don’t think it’s a real issue.

At the start of the spider scenario, it felt very realistic. I was surprised and a little tense. But since I knew it was virtual, the experience overall was still good. But in the contaminated VR environment, I actually felt that it was dirty and unpleasant—because of the stronger immersion.

**Interviewer:** Based on your experience, do you think AR and VR are suitable for virtual exposure therapy?

**Participant 29:** Yes, both can work. AR has the advantage of being safer and allowing for real-time supervision. In VR, participants might move around or lose awareness of the real environment, which could be risky without supervision. VR offers stronger immersion, which might be more effective but also needs careful monitoring. I think VR is better for treatment that needs more safety and guidance, especially for people with strong phobias.

**Interviewer:** Do you think AR and VR are better suited for different stages of therapy?

**Participant 29:** Maybe there’s no fixed rule, but based on today’s test, transitioning from AR to VR might be a good approach. AR helps participants build up confidence in a familiar setting, and then they can move on to VR for a more immersive challenge.

**Interviewer:** Do you have any feedback or suggestions for improving the prototype?

**Participant 29:** Overall, it was well done. Maybe adding sound effects or improving weight simulation, like making the trash or spiders feel heavier when they fall, would enhance realism. But I also don’t think it needs to be 100% realistic. If the spider becomes *too* real, it might be counterproductive. I think the current level of realism is appropriate. For the spider, maybe it could react more dynamically. Right now it just struggles in a loop when picked up. It could jump away or act more unexpectedly. But overall, the experience was smooth. I felt comfortable with the design and was able to attempt interactions with the spider, so that was good.

**Interviewee: Participant 30
Date: August 11, 2024, 1:52PM**

**Interviewer:** A brief interview. I’ll just ask some additional questions. First of all, could you describe your main feelings about this experience? You could talk about AR and VR technologies, or the differences between Project One and Two — the spider and contamination environments. You can focus on realism or immersion.

**Participant 30:** Sure. In most of the experiment, it was the spiders, and in the AR environment when they were on the table, I didn’t feel scared to touch them. It felt more like a game, so it didn’t feel real to me. But in the VR environment, when there was more context around me, it felt much more real like the spiders were in my room. At that point, I did feel a bit skeptical about interacting with them. But in AR, I was just picking them up and playing with them like any toy. It didn’t feel real.

For the dirty environment, that one really triggered me. I’m not comfortable with dirty places. In VR, it felt much more real. The ground looked dirtier. I felt more immersed in the space. When the contaminated liquid was under my feet, it felt incredibly real, as if I were stepping on something sticky. I became much more conscious of where I was stepping. When I reached out to pick something up, I felt like I was grabbing actual rubbish. So the VR environment was much more impactful.

**Interviewer:** So you felt differently between Project One and Two — the spider and contamination environments?

**Participant 30:** Yes. Project Two (the contamination one) felt very real in both AR and VR, but VR was much more immersive. AR was also immersive, but not as strong.

**Interviewer:** Okay. So for the preference questions in the questionnaire, you chose VR for both the spider and contamination projects, right?

**Participant 30:** Yes.

**Interviewer:** Why did you prefer VR for the contamination environment?

**Participant 30:** Because in VR, there were objects all around me, and it really felt like I was in a messy space. In AR, I always knew I was just in this room. In the back of my mind, I knew it wasn’t real. But in VR, it felt completely real.

**Interviewer:** Did you encounter any difficulties during the process — mentally or physically?

**Participant 30:** Yes — in the VR contamination scene, when I was moving around, it felt a bit shaky. That was a bit off.

In the VR spider experience, I wasn’t sure where I was going. I was worried I might crash into something, so that made me a little hesitant.

**Interviewer:** Based on your experience, do you think AR and VR are suitable tools for exposure therapy?

**Participant 30:** Yes, I think both are suitable. But for spiders, AR didn’t work for me at all. For garbage, AR also didn’t work well — VR was much better for both.

**Interviewer:** Do you think these technologies might be suitable for other types of therapy?

**Participant 30:** Yes. I think AR might be suitable for food-related therapies. There are lots of food disorders, and with AR you still get the context of the environment you’re in — like where you’re eating, which can matter. So I think AR could be better for that kind of therapy.

**Interviewer:** Great. Do you have any additional feedback or suggestions?

**Participant 30:** I don’t know if this is related to the experiment, but maybe if you increase the size of the spiders — make them a bit bigger or more diverse — it would feel much more real in AR. Right now the spiders felt really small to me. But overall, I think this was a very fun and interesting experience.

**Interviewer:** Thank you!

**Interviewee: Participant 31
Date: August 12, 2024, 11:42AM**

**Interviewer:** So first, could you describe your main feelings about the experience? You could talk about the differences between AR and VR, or between Project One and Two — like the spider and contamination scenes. You can focus on aspects such as realism or immersion.

**Participant 31:** In terms of interaction with the objects themselves, I didn’t feel much difference between AR and VR. The manipulation was quite similar.

But for the overall environment, I think VR had a higher threshold for realism. From my own experience I’ve studied some environmental modelling, I think realism is very important. Right now, the VR environments felt a bit too cartoonish, which weakened my sense of realism.

Also, in AR, I could see my full limbs, so I felt more present in the environment. In VR, I could only see my hands, not my legs. When I was looking straight ahead, it felt real. But when I was told to look down to interact with the garbage on the ground, I suddenly felt like I didn’t exist in the space — because I couldn’t see my feet. That broke the immersion for me. When I bent down to pick up trash, it felt quite strange.

**Interviewer:** And did you feel any difference between the spider and garbage scenes?

**Participant 31:** Yes. The spider scene felt more real to me, especially when I picked up the spider and saw its legs moving in front of me. It gave me the illusion of touch, like I was actually holding the spider. Especially when its legs started twitching quickly just like a real insect when you grab it. That made it feel very authentic. For the garbage scene, the most realistic moment was when I picked up the banana peel. Due to tracking issues, it fell from my hand, but that actually made it feel more like a real object. The way it slipped from my grip when I rotated my hand gave me a sense of physical realism. But overall, the spider interaction felt more authentic than the garbage one.

**Interviewer:** Did you encounter any difficulties during the process, mentally or physically?

**Participant 31:** I bumped my head into a real-world object because the system didn’t detect it — that was one issue. Another problem was hand tracking — when I generated the spider, I moved my hand down too quickly and ended up slapping it. It dropped immediately. So I hope hand recognition can be more accurate. Also, in VR, the virtual walls didn’t align with the real walls, so I wasn’t aware of my physical boundaries. Unlike AR, where I could clearly see where things were and felt safer. In VR, I had to be more cautious, and I actually bumped into something when throwing trash.

**Interviewer:** In the preference questions, which technology did you choose for each project?

**Participant 31:** I chose AR for the spider scene and VR for the garbage scene.

**Interviewer:** Can you explain why?

**Participant 31:** For the garbage scene, VR made the surroundings feel much dirtier — everything was messy, so it felt like a consistent environment. In AR, the space around me was clean, with organized furniture, and only one area had garbage. That broke the realism. For the spider scene, I don’t know exactly why, but maybe it was the order of the experiences. The AR version felt more real. When I picked up the spider and saw it move in the real environment, the sense of presence was stronger.

**Interviewer:** Lastly, do you have any feedback or suggestions for the prototype?

**Participant:** I hope the rendering and modeling proportions can be improved. These strongly affect my perception of realism. One reason I preferred the AR spider scene was that the surrounding furniture was more accurately scaled. But in the VR scene, the chair was comically large, which made it feel unrealistic and disrupted my judgment of the environment.

**Interviewer:** Got it. Thank you so much!
